# Oncolytic Virus‐Engineered Extracellular Vesicles Rewire Glioblastoma Immunity

**DOI:** 10.1002/jev2.70389

**Published:** 2026-10-21

**Authors:** Klaudia Kiel, Arpita Balakrishnan, Aleksandra Cabaj, Luiza Stanaszek, Maciej Przybylski, Dawid Stępnik, Marta Małuszek, Christopher Jannotta, Chiara Zoe Linke, Joanna Suszyńska‐Zajczyk, Monika Knaur, Agnieszka Szczypiorowska, Szymon Hryhorowicz, Bianka Świderska, Katarzyna Piwocka, Alexander Lee Ling, E. Antonio Chiocca, Agnieszka Bronisz, Jakub Godlewski

**Affiliations:** ^1^ Tumor Microenvironment Laboratory Mossakowski Medical Research Institute Polish Academy of Sciences Warsaw Poland; ^2^ Translational Medicine Doctoral School Center of Postgraduate Medical Education Warsaw Poland; ^3^ Laboratory of Sequencing, Nencki Institute of Experimental Biology Polish Academy of Sciences Warsaw Poland; ^4^ NeuroRepair Department, Mossakowski Medical Research Institute Polish Academy of Sciences Warsaw Poland; ^5^ Department of Medical Microbiology Medical University of Warsaw Warsaw Poland; ^6^ The Laboratory of Cytometry Nencki Institute of Experimental Biology Polish Academy of Sciences Warsaw Poland; ^7^ Department of Neurosurgery Brigham and Women's Hospital, Harvard Medical School Boston Massachusetts USA; ^8^ Institute of Human Genetics Polish Academy of Sciences Poznań Poland; ^9^ Department of Biochemistry and Biotechnology Poznań University of Life Sciences Poznań Poland; ^10^ Mass Spectrometry Laboratory Institute of Biochemistry and Biophysics, Polish Academy of Sciences Warsaw Poland; ^11^ Department of NeuroOncology Mossakowski Medical Research Institute, Polish Academy of Sciences Warsaw Poland

**Keywords:** extracellular vesicles (EVs), glioblastoma (GBM), immunotherapy, Oncolytic Herpes simplex Virus (oHSV‐1), PBMC‐engrafted mouse model, PBMC‐GSC organoid

## Abstract

Immunotherapy with oncolytic *Herpes simplex* virus type 1 (oHSV‐1) shows promise for glioblastoma; however, clinical benefit remains uncommon due to tumour‐ and host‐mediated immunosuppression, which limits immune targeting of residual glioblastoma stem‐like cells (GSCs). To address this barrier, extracellular vesicles (EVs) harvested from oHSV‐1‐infected GSCs (EVOV) were evaluated for their immunomodulatory capacity by priming peripheral blood mononuclear cells (PBMCs) *ex vivo* and *in vivo*. In GSC‐PBMC organoids analysed by single‐cell RNA sequencing, IFN‐response and antigen‐presentation programs were induced in both immune and tumour compartments, despite the GSC state. EVOV organoid transcriptome associated with patient tumour antigen‐processing programs linked to improved clinical outcome, and EVOV immunopeptidomics identified candidate biomarker peptides that were also detected in patient immunopeptidomes from the clinical‐trial cohort. In PBMC‐engrafted NOD *scid* gamma mice bearing intracranial GSC xenografts, EVOV delivery increased circulating interferon (IFN) levels and enhanced systemic activation of T‐cell subsets, while spatial transcriptomics revealed recruitment of peripheral immune cells to the tumour, accompanied by reprogramming of GSC states. Blocking vesicle‐associated major histocompatibility complex I (MHC‐I) attenuated EVOV‐driven PBMC cytotoxicity against GSCs, supporting an MHC‐I peptide‐dependent mechanism of immune priming and a vaccine‐ or adjuvant‐like, non‐infectious, vesicle‐based immunotherapeutic strategy.

## Graphical abstract

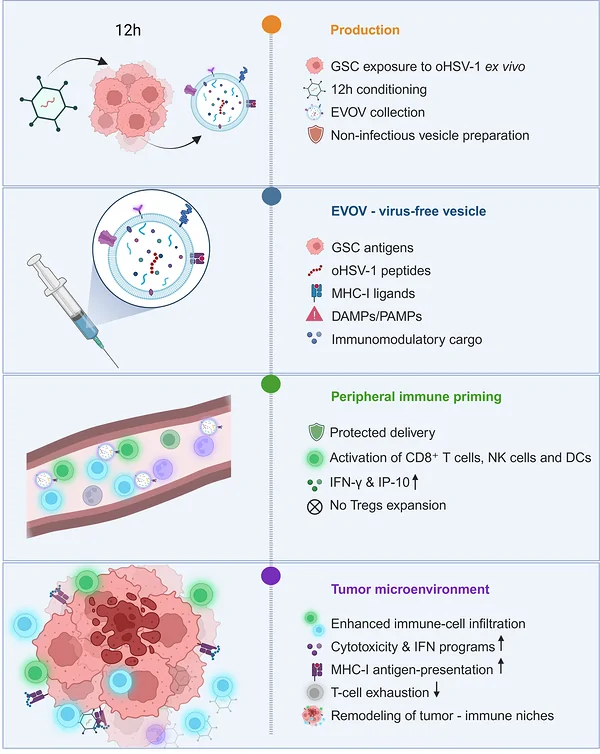

## 1 Introduction

Glioblastoma (GBM), a WHO grade 4 malignancy (Louis et al. 2021), stands as one of the most immune‐resistant solid tumours, evading therapeutic control despite decades of intensive effort. Standard‐of‐care surgical resection followed by radiotherapy and temozolomide offers only temporary respite, with nearly universal recurrence and a median survival of approximately 14–16 months (Stupp et al. 2005). The failure of immune checkpoint blockade and other immunotherapies highlights the profound immune inadequacy within the GBM microenvironment, characterized by the prevalence of immunosuppressive myeloid and regulatory T cells (Tregs), dysfunctional antigen‐presenting cells (APC), and a scarcity of cytotoxic T cells (Hambardzumyan et al. 2016, Lim et al. 2018). Key contributors to this dire immune landscape are GBM stem‐like cells (GSCs), which exhibit dynamic transcriptional plasticity and often adopt a mesenchymal (MES) or hybrid state (Patel et al. 2014, Neftel et al. 2019). These are associated with the activation of inflammatory pathways, promoting immune evasion through increased expression of immune checkpoints such as PD‐L1, and resistance to T cell‐mediated cytotoxicity (Cloughesy et al. 2019, Richards et al. 2021). The persistence of GSCs despite standard‐of‐care drives tumour recurrence, highlighting the urgent need for immunotherapeutic and vaccine‐like strategies to eliminate these resistant cells and prevent relapse (Schäfer et al. 2019).

Oncolytic viruses (OVs) are promising therapeutic agents that combine direct tumour cell lysis with immune activation (Peruzzi and Chiocca 2018). Engineered oncolytic *Herpes simplex* virus type 1 (oHSV‐1) (Ling et al. 2023, Todo et al. 2022) selectively replicates in cancer cells and induces immunogenic cell death, promoting infiltration of memory and cytotoxic T cells and reducing Tregs (Todo et al. 2025). A recent Phase I trial in recurrent GBM (rGBM; NCT03152318) demonstrated that patients with the longest survival were oHSV‐1 seropositive, showed increased changes in tumour‐infiltrating CD8+/CD4+ T cells and T cell clonotypes with greater diversity, and exhibited tumour transcriptomic signatures of immune activation (Ling et al. 2023). Despite these advances, the efficacy of oHSV‐1 can still be improved (Patel et al. 2014). Mechanistically, exposure to oHSV‐1 reprograms the cancer secretome, altering not only soluble factors but also the composition of extracellular vesicles (EVs). These vesicles encapsulate viral and tumour antigens, damage‐associated molecular patterns (DAMPs), and immune modulators, trafficking them through the endosomal system during the early pre‐replication stage of viral infection (Godlewski et al. 2021, Nakashima et al. 2015), thus offering a naturally compartmentalized and safer route for delivering immunogenic cues.

Importantly, this EV‐mediated component of oncolytic virotherapy is not restricted to GBM or oHSV‐1. Recent studies in non‐CNS tumour models have shown that OV infection can alter EV release, vesicle composition, and intercellular immune communication. In melanoma, thoracic cancer, ovarian cancer, and reovirus‐based tumour models, OV‐conditioned EVs have been reported to carry viral material, tumour‐associated antigens, DAMP‐related signals, and immune‐regulatory molecules, thereby supporting innate immune activation, myeloid cell remodelling, and anti‐tumour responses (Hirigoyen et al. 2024, Shuwari et al. 2024, Jennings et al. 2024). In addition, EVs have been explored as delivery vehicles for oncolytic adenoviruses, improving viral protection, tumour targeting, and therapeutic activity (Saari et al. 2020, Garofalo et al. 2019). These studies support the concept that EVs can contribute to OV‐induced immunogenicity by transferring viral and tumour‐associated signals to the tumour microenvironment (TME), providing a broader rationale for developing cell‐free, OV‐conditioned vesicle‐based immunotherapeutic strategies (Hirigoyen et al. 2024, Shuwari et al. 2024, Jennings et al. 2024, Saari et al. 2020, Garofalo et al. 2019, Safarzadeh et al. 2025).

The current study developed EVs from GSCs infected with oHSV‐1, termed EVOV. These vesicles were harvested *ex vivo* during the early phase of oHSV‐1 infection to assess their impact on immunophenotype.

To evaluate the immune properties of vesicles from multiple patient‐derived GSCs, EV and EVOV were used to prime peripheral blood mononuclear cells (PBMCs) *ex vivo*. Downstream analysis by MPFC showed that EVOVs increased the frequency of activated cytotoxic subsets, including CD8^+^ and CD4^+^ T cells, NK cells, and dendritic cells (DCs). Subsequently, vesicle‐primed PBMCs were used to develop a GSCs‐PBMC organoid model, followed by multiplex imaging (MPI), MPFC, and single‐cell transcriptomics (scRNA‐seq), and the activity of PBMCs against parental GSCs was characterized. In addition to the cytotoxic effect of immune cells on GSCs, EVOV‐primed PBMCs reprogrammed GSC subpopulations, promoting IFN signalling and upregulating MHC‐I antigen presentation pathways, while altering inter‐subpopulation ratios. These subpopulations correspond to the core transcriptional states identified in GSCs: neural progenitor‐like (NPC), oligodendrocyte progenitor‐like (OPC), and mesenchymal‐like (MES), which exhibit plasticity and are linked to immune evasion and treatment resistance (Neftel et al. 2019).

Intracranial GSC xenografts were established in NOD *scid* gamma (NSG) mice engrafted with human PBMCs, followed by intraperitoneal administration of EVs or EVOVs. This model allows analysis of systemic and intratumourally immunophenotypes. Multiparameter flow cytometry (MPFC) demonstrated that immune cells responded systemically to EVOV treatment, resulting in T cell activation and elevated circulating interferon (IFN)‐γ levels. Additionally, exploratory analysis of VisiumHD spatial transcriptomics of brain tissue revealed immune cell infiltration within the TME in mice.

Next, ultra‐deep data‐independent acquisition mass spectrometry (DIA‐MS) was applied to characterize the EVOV peptidome, revealing that EVOV exhibits a remodelled, more immunogenic MHC‐I‐associated peptide landscape, enriched in posttranslational modifications (PTM)‐bearing peptides, including those detected directly from serial tumour biopsies of GBM patients treated intratumourally with oHSV (CAN‐3110) (Ling et al. 2025). Blocking MHC‐I on vesicles reduces PBMC cytotoxicity and restores sphere‐forming capacity, supporting an MHC‐I–dependent mechanism of PBMC anti‐tumour activity. Importantly, EVOV‐induced immune and antigen‐presentation programs were associated with improved survival in the CAN‐3110 trial cohort, supporting clinically relevant potential of EVOV.

In summary, EVOVs encapsulate patient‐specific tumour antigens and innate adjuvants, enhancing cytotoxic phenotypes among immune cells. When delivered systemically, vesicle dosing was well tolerated, supporting their feasibility as non‐injurious immunomodulatory inputs in PBMC‐engrafted NSG mice.

## 2 Methods

### 2.1 GSCs

GSCs, isocitrate dehydrogenase (IDH)‐ wild‐type with subtype classification (Machura et al. 2015, Tognetti et al. 2022), were obtained from surgical specimens at Brigham and Women's Hospital under institutional review board (IRB)‐approved protocols (2013P001236). Informed consent was obtained from all patients before tissue collection. Mycoplasma contamination testing was performed regularly, and all cell lines tested negative.

### 2.2 PBMCs

PBMCs were isolated from buffy coats obtained from healthy donors (Regional Blood Center, Warsaw) in accordance with institutional and national regulations under protocols approved by the Ethics Committee of the Center of Postgraduate Medical Education (WAW/111/2021).

### 2.3 NSG Mice

Five‐week‐old female NSG (NOD.Cg‐Prkdc^scid Il2rg^tm1Wjl/SzJ) mice were obtained from AnimaLab and housed in individually ventilated cages under specific pathogen‐free conditions at the institutional animal facility. Animals were group‐housed (4‐5 per cage) with ad libitum access to water and irradiated rodent chow. Temperature (22 ± 2°C), humidity (55 ± 10%), and 12‐h light/dark cycles were maintained. All procedures were performed in accordance with institutional and national guidelines, with prior approval from the Local Ethics Committee for Animal Experiments (WAW/138/2023). Allocation to treatment groups was randomized.

### 2.4 GSC Culture

GSCs were cultured under serum‐free conditions in Neurobasal medium supplemented with B‐27 without Vitamin A, GlutaMAX, recombinant human basic fibroblast growth factor (bFGF), and recombinant human epidermal growth factor (EGF). Cultures were maintained at 37°C in a humidified atmosphere with 5% CO_2_, using ultra‐low attachment plates to promote neurosphere formation.

### 2.5 PBMC Isolation and Culture

PBMCs were isolated by density gradient centrifugation using Lymphoprep according to the manufacturer's instructions. Following isolation, cells were cultured in RPMI‐1640 medium supplemented with 10% foetal bovine serum (FBS) and 1% penicillin‐streptomycin. Cultures were maintained at 37°C in a humidified incubator with 5% CO_2_. PBMCs were used fresh for downstream applications. Cell viability was routinely assessed by trypan blue exclusion before experimental use.

### 2.6 PBMC‐Engrafted NSG Mice with Intracranial GSCs Xenograft

On day 0, 20 × 10^6^ human PBMCs were injected intravenously *via* the lateral tail vein in 0.2 mL of PBS using a 1 mL tuberculin syringe and a 25G × ⅝‐in. needle. Each injection was completed within 30–60 s, followed by monitoring for injection‐related complications.

On day 1, mice were anesthetized *via* intraperitoneal injection of a ketamine‐Domitor mixture and stereotactically implanted with 1 × 10^4^ viable GSC‐G62 cells transduced with pCMV‐Fluc. Tumour cells were delivered intracranially at coordinates 2 mm right lateral, 1 mm anterior to bregma, and 3 mm depth. Cell number and viability were assessed using the Muse Cell Analyzer before implantation. Post‐surgery, mice received antibiotic treatment (Enrocin) and analgesics (Tolfine) administered *via* intraperitoneal injection. After the procedure, anaesthesia was reversed using the drug Alzane, administered intraperitoneally to facilitate recovery.

On day 2, mice received a single intraperitoneal dose of EV or EVOV treatment at 120 µg total EV/EVOV protein per animal. No additional EV or EVOV doses were administered during the experiment. Tumour progression was monitored by intraperitoneal injection of D‐luciferin, followed by bioluminescence imaging (BLI) with the *In Vivo* Imaging System (IVIS) Spectrum. Mice were euthanized on day 15, and brains were collected for Formalin‐Fixed Paraffin‐Embedded (FFPE) embedding or cryosectioning.

At the experimental endpoint, mice were euthanized *via* intraperitoneal injection of a ketamine and Domitor mixture. Blood was collected from the right ventricle, incubated with lysis buffer to remove erythrocytes, and centrifuged at 200 × g for 10 min. Recovered cells were used for downstream MPFC analysis.

### 2.7 OHSV‐1 Production

The recombinant oHSV‐1 vector rQNestin34.5v1, engineered to express GFP, was used under authorization from the Polish Ministry of Climate and Environment (Decision No. 26/2023). This vector retains the viral neurovirulence *ICP34.5* gene, transcribed by a nestin promoter to confer preferential tumour replication—a strategy used in clinical trials with CAN‐3110, an oHSV‐1 engineered for GBM therapy (NCT03152318) (Ling et al. 2023). The virus was obtained from Brigham and Women's Hospital and transferred to MMRI PAS under a Material Transfer Agreement. The replication‐competent recombinant *Herpes simplex* virus type 1 (oHSV‐1) rQNestin34.5v1, engineered to express GFP, was amplified in Vero cells (African green monkey kidney epithelial cells) obtained from Brigham and Women's Hospital. Vero cells were cultured in Dulbecco's Modified Eagle Medium (DMEM) supplemented with 2% heat‐inactivated FBS and 1% penicillin‐streptomycin, and maintained at 37°C in a humidified incubator with 5% CO_2_. For virus amplification, cells were seeded in T175 flasks and infected with oHSV‐1 at a multiplicity of infection (MOI) of 0.01. Infection progressed for 48–72 h until complete cytopathic effect (CPE) was observed, as monitored by GFP fluorescence. Infected cells underwent three freeze‐thaw cycles and were vortexed for 45 min to release viral particles. Lysates were clarified by centrifugation at 2000 × *g* for 10 min at 4°C, filtered through a 0.8 µm syringe filter, and concentrated by centrifugation at 15,000 × *g* for 60 min in PBS supplemented with 0.45 M NaCl and 100 µg/mL dextran sulphate. Viral titters were determined by plaque assay on Vero cells and by the Reed‐Muench endpoint dilution method based on CPE and GFP expression. Final preparations were aliquoted and stored at ‐80°C until use.

### 2.8 OHSV‐1 Infection of GSCs

GSCs were maintained in serum‐free Neurobasal medium supplemented with B27, GlutaMAX, basic bFGF, and EGF and cultured on ultra‐low‐attachment plates to support neurosphere formation. For viral infection, GSCs were seeded in suspension and exposed to oHSV‐1 at an MOI of 0.1 for 12 h in serum‐free Neurobasal medium. Following infection, cells were washed and further processed for downstream applications.

### 2.9 EV and EVOV Isolation

Conditioned medium from infected or uninfected GSC cultures was collected and subjected to sequential centrifugation at 300 × g for 10 min and 2000 × g for 10 min at 4°C to remove cells and debris. The supernatant was filtered through a 0.22 µm syringe filter and ultracentrifuged at 100,000 × g for 70 min at 4°C. The resulting EV or EVOV pellet was resuspended in plain RPMI medium without additional washing steps and used immediately for analysis. To evaluate potential co‐isolated soluble factors, preparations were profiled by ultradeep DIA‐MS, which did not detect cytokines/chemokines above the method's detection threshold and confirmed enrichment of EV‐associated proteins.

### 2.10 EV and EVOV Characterization

EVs were characterized using nanoparticle tracking analysis (NTA) performed on a NanoSight NS300 system. Vesicle morphology was assessed by transmission electron microscopy (TEM) using a Tecnai T12 microscope. Total protein content of EV and EVOV preparations was measured using the BCA Protein Assay Kit according to the manufacturer's protocol. All measurements were conducted on freshly isolated vesicles.

### 2.11 TEM

Ten microliters of EVs and EVOVs were spotted onto copper grids (400 mesh) coated with Formvar (Ted Pella) and carbon for 1 min. The grids were then stained with 2% (w/v) uranyl acetate (SPI Supplies) for 40 s. After air‐drying, samples were imaged using a transmission electron microscope (JEM‐1400, JEOL Co.) equipped with an 11‐megapixel TEM camera (MORADA G2, EMSIS GmbH). The microscopic analysis was repeated multiple times, and representative images are presented.

GSC pellets were fixed in a solution containing 2.5% glutaraldehyde and 2% paraformaldehyde in 0.1 M sodium cacodylate (NaCac) buffer (pH 7.4) at 4°C for 24 h. Post‐fixation was performed with 2% osmium tetroxide (OsO_4_) in NaCac buffer for 1 h, followed by en bloc staining with 2% aqueous uranyl acetate. After dehydration in a graded acetone series, samples were embedded in Epon resin and polymerized for 2 days at 60°C. Ultrathin sections (∼60 nm) were cut using a diamond knife on an Ultracut R ultramicrotome (Leica Microsystems, Vienna, Austria), collected on copper grids, and stained with Reynolds’ lead citrate for 2 min. Imaging was performed using a JEM‐1400 transmission electron microscope (JEOL Co., Japan, 2008) operated at 80 kV and equipped with an 11‐megapixel TEM camera (MORADA G2, EMSIS GmbH, Germany).

### 2.12 Quantitative Detection of oHSV‐1 DNA in EVs

EVs or EVOVs were isolated from 0.9‐2.4 × 10^6^ GSCs treated with mock or oHSV‐1. DNA was extracted from purified EVs/EVOVs using the NucleoSpin Tissue Kit, following the manufacturer's protocol, with a modified lysis step shortened to 15 min to account for the sample's high lysis efficiency. Quantitative detection of oHSV‐1 DNA was performed using two independent qPCR approaches to ensure methodological robustness. The first approach used a commercial HSV‐1/2 R‐Gene assay, performed according to the manufacturer's instructions. The second approach was an in‐house qPCR method previously described (Machura et al. 2015), using the SensiFAST Probe No‐ROX Kit for amplification.

All qPCR reactions were carried out on a CFX96 Real‐Time PCR Detection System. Both methods targeted conserved HSV‐1 genomic regions, and quantification was performed using standard curves generated from known viral DNA concentrations. Technical replicates and negative controls were included in each run.

### 2.13 RNA Isolation, cDNA Synthesis, and Quantitative qPCR

Total RNA was extracted from GSCs (*n* = 6) using TRIzol reagent according to the manufacturer's protocol. Briefly, cells were washed with ice‐cold PBS and lysed in 1 mL of TRIzol per well. After phase separation with chloroform, RNA was precipitated with isopropanol, washed with 75% ethanol, and resuspended in RNase‐free water. RNA concentration and purity were measured spectrophotometrically using NanoDrop.

cDNA was synthesized using the High‐Capacity cDNA Reverse Transcription Kit with one µg total RNA per reaction. Synthesized cDNA was stored at −80°C until further use.

qPCR was performed using PowerUp SYBR Green Master Mix on a StepOnePlus Real‐Time PCR System. Each 20 µL reaction contained 3 µL of cDNA (30 ng/reaction). Amplification specificity was verified by melt curve analysis. Relative gene expression was calculated using the ΔΔCt method and normalized to GAPDH.

### 2.14 Protein Quantification (BCA Assay)

Protein concentration in EV/EVOV preparations and GSC lysates was measured using the BCA Protein Assay Kit, following the manufacturer's protocol. Samples and BSA standards were loaded in duplicate onto a 96‐well plate and incubated at 37°C for 30 min with shaking. Absorbance was measured at 562 nm using a plate reader.

To ensure consistency, all standards were prepared in RIPA buffer. EV/EVOV samples were sonicated before assay to ensure complete vesicle lysis, whereas GSC lysates were processed without sonication.

### 2.15 Western Blot

EV/EVOV and GSC lysates were prepared in RIPA buffer supplemented with protease inhibitors and quantified by BCA assay. Equal amounts of protein were separated by SDS‐PAGE using 4%–12% gradient gels and transferred onto nitrocellulose membranes.

Membranes were blocked with 5% non‐fat dry milk in TBS‐T and incubated overnight at 4°C with primary antibodies targeting EV and GSC markers. After washing, membranes were incubated with HRP‐conjugated secondary antibodies (1:10,000 dilution) for 1 h at room temperature. Signal detection was performed with an enhanced chemiluminescence (ECL) substrate, and blots were visualized with a ChemiDoc imaging system. Blot images were cropped to highlight proteins of interest; full, uncropped scans are available from the Lead Contact upon request.

### 2.16 Dia‐Ms

EV and EVOV samples were shipped frozen and processed by Biognosys AG in accordance with its internal standard operating procedure. Protein lysates were reduced, alkylated, and digested with trypsin at a 1:50 protease‐to‐protein ratio overnight at 37°C. Peptides were desalted using HLB µElution plates, dried in a SpeedVac, and resuspended in 1% acetonitrile and 0.1% formic acid containing iRT calibration peptides.

Peptide concentrations were determined using a microBCA assay. For DIA LC‐MS/MS acquisition, 2 µg of peptide per sample was loaded onto an in‐house‐packed reversed‐phase analytical column coupled to a Thermo Scientific NeoVanquish UHPLC system and an Orbitrap Exploris 480 mass spectrometer equipped with a Nanospray Flex ion source and FAIMS Pro interface.

A nonlinear gradient from 1% to 50% solvent B (80% acetonitrile, 0.1% formic acid) was applied over 172 min, followed by a 5‐min wash at 90% B and re‐equilibration at 1% B. The flow rate was adjusted from 500 to 250 nL/min during the gradient. DIA was performed using a FAIMS interface with full MS1 scans and 34 DIA segments per compensation voltage, as previously described (Tognetti et al. 2022, Bruderer et al. 2017).

Raw data were processed using Spectronaut v18.4 with a directDIA+ in‐house generated spectral library. Searches were conducted against the UniProt human reference proteome (2023‐07‐01) and oHSV‐1 proteome (UP000009294, 2023‐10‐16). Two missed cleavages and variable modifications (N‐terminal acetylation, methionine oxidation, phosphorylation on serine/threonine/tyrosine, and lysine glycation [Hex(K)]) were allowed. Global peptide and protein FDR thresholds were set to 1%. No imputation was applied. Cross‐run normalization was performed using local regression (Callister et al. 2006).

To identify HLA‐presented peptides, data were searched against a composite immunopeptidome reference database compiled from the HLA Ligand Atlas (Marcu et al. 2021), MHC Motif Atlas (Tadros et al. 2023), and Immune Epitope Database (IEDB) (Vita et al. 2025). Peptide‐spectrum matches were filtered at 1% FDR and manually verified. Functional annotation of identified peptides was performed using Gene Ontology enrichment analysis.

### 2.17 PBMC Priming with EVs or EVOVs

PBMCs from healthy donors (*n* = 8) were primed with EVs or EVOVs at a single dose of 20 µg total EV/EVOV protein per 3.5 × 10^5^ cells. For dose‐escalation viability analyses, PBMCs were additionally primed in monoculture with 50 µg total EV/EVOV protein per 3.5 × 10^5^ cells under the same culture conditions. Each donor was primed with EVs or EVOVs derived from three distinct GSC lines (*n* = 6 total). Donors were divided into two independent experimental sets, each receiving vesicles from a unique GSC subset to minimize inter‐donor and inter‐vesicle variability.

PBMCs were seeded at a density of 1 × 10^6^ cells/mL in 6‐well plates in RPMI‐1640 medium supplemented with 10% FBS and 1% penicillin‐streptomycin. Priming was performed in a final volume of 1 mL per well without medium change and maintained for 72 h at 37°C in a humidified incubator with 5% CO_2_. After priming, PBMCs were washed with PBS and either processed for MPFC or used in downstream PBMC‐GSC organoid assays.

### 2.18 MPFC

PBMCs were stained using a 17‐color MPFC spectral flow cytometry panel (Kiel et al. 2026) designed to assess major immune cell subsets, activation, and exhaustion markers. The antibody panel included CD3 (2.5:100), CD4 (1.25:100), CD8 (2.5:100), CD25 (2:100), CD69 (3.5:100), PD‐1 (3.5:100), TIM‐3 (3.5:100), FOXP3 (1:100), CD14 (5:100), CD16 (2.5:100), CD11c (2:100), CD56 (2.5:100), HLA‐DR (2.5:100), CD303a (2.5:100), CD83 (5:100), and CD45 (4:100). Viability was assessed using a fixable viability dye (LIVE/DEAD Fixable Dead Cell Stain) (1:1000) at room temperature/ 30 min. Cells were incubated with the extracellular antibody cocktail in Super Bright Complete Staining Buffer at 2–8°C for 30 min, fixed and permeabilized using the Foxp3 Fixation/Permeabilization kit, and stained for intracellular markers at room temperature for 30 min. Samples were resuspended in Flow Cytometry Staining Buffer (PBS + 2% BSA) and acquired on a Cytek Aurora spectral flow cytometer. Spectral unmixing was performed using Cytek SpectroFlo 3.2.1 software.

Flow cytometry data were analysed using FlowJo v10.10 (BD Life Sciences). Flow Cytometry Standard (FCS) 3.0 files were cleaned using the FlowAI algorithm (Monaco et al. 2016) and compensated. Data were preprocessed by removing damaged cells and doublets. Live, undamaged CD45^+^ CD3^+^ (CD3^+^ cell subset) and CD45^+^ CD3^−^ (CD3^−^ cell subset) were then selected. Subsets were normalized using the gaussNorm method from flowStats (version 4.14.1) in R 4.3.1 if batch effects were identified. Further analyses were performed using Bioconductor libraries: CATALYST (version 1.26.1) (Nowicka et al. 2017) and diffcyt (version 1.23.1) (Weber et al. 2019). Marker expression was arcsinh‐transformed, with marker‐specific cofactors, to make the distributions more symmetric and map them to a comparable expression range. Cell population identification was performed through unsupervised clustering using the FlowSOM (version 2.1.0) (Van Gassen et al. 2015) algorithm (*K* = 30 for CD3^+^ and *K* = 40 for CD3^−^). FlowSOM was trained on all acquired samples for each cell subset. Clusters with similar distributions were then merged.

In the CD3^+^ compartment, CD4 T cells were defined by CD3^+^CD4^+^CD8^−^ profiles, CD8 T cells by CD3^+^CD8^+^CD4^−^ profiles, Tregs by CD3^+^CD4^+^FoxP3^+^CD25^+^ profiles, NKT‐like cells by combined CD3 and CD56 expression, and double‐negative or double‐positive T cells by the absence or co‐expression of CD4 and CD8, respectively. Activation‐associated phenotypes were annotated by increased expression of CD69, CD25, CD83, and/or HLA‐DR, whereas checkpoint/exhaustion‐associated phenotypes were annotated by increased PD‐1 and/or TIM‐3 expression. Additional unconventional T‐cell clusters were annotated when T‐cell lineage markers co‐occurred with monocyte‐ or dendritic‐cell‐associated markers, such as CD14, CD16, or CD11c.

In the CD3^−^ compartment, NK‐cell clusters were defined by CD56 and/or CD16 expression in the absence of CD3; monocyte clusters by CD14 and/or CD16 expression; classical monocytes by CD14^+^CD16^−^ profiles; intermediate monocytes by CD14^+^CD16^+^ profiles; and non‐classical monocytes by CD14^−^CD16^+^ profiles. Macrophage‐like clusters were annotated based on monocyte‐lineage marker expression together with increased HLA‐DR and activation/checkpoint‐associated markers such as CD83, CD25, and/or PD‐1. HLA‐DR defined DC‐like clusters with dendritic‐cell‐associated markers, including CD11c and/or CD83, whereas pDC‐like clusters were defined by CLEC4C/CD303a expression. Labels such as ‘classical‐MO switching to DCs’, ‘intermediate‐MO switching to DCs’, ‘intermediate‐MO switching to pDCs’, and ‘CD25^+^ switching to pDCs’, denote FlowSOM‐defined transitional phenotypes with combined monocyte‐associated and dendritic‐cell‐associated marker expression, primarily involving CD14/CD16 together with HLA‐DR, CD11c, CD83, CD25, CD56, and/or CLEC4C/CD303a. Data were split into subsets as indicated in the Figure legend. 2D visual representation was generated using Uniform Manifold Approximation and Projection (UMAP) on 10,000 cells per sample.

### 2.19 PBMC‐GSC Organoid Assay

PBMCs from healthy donors, primed with a single dose of EV or EVOV, were co‐cultured with non‐infected parental GSC spheroids (*n* = 3) at a 10:1 PBMC:GSC ratio or infected with oHSV‐1 24 h before adding PBMCs. GSC spheroids were seeded in 12‐well Nunclon Sphera plates in serum‐free conditions at 1 × 10^5^ cells/well and grown for 72 h. Primed PBMCs were added to the GSC spheroids after 72 h of pre‐incubation with 20 µg EV or EVOV protein per 3.5 × 10^5^ cells. Organoid cultures were maintained in RPMI‐1640 medium supplemented with 10% FBS and 1% penicillin‐streptomycin for 72 h at 37°C in a humidified 5% CO_2_ atmosphere. At the endpoint, PBMC‐GSC organoids were harvested for MPFC, scRNA‐seq, and MPI.

For MPFC analysis, organoids were generated using four GSC lines (*n* = 4) with PBMCs from four donors (*n* = 4). For scRNA‐seq, organoids were generated from three selected GSC lines (*n* = 3) and one PBMC donor (*n* = 1). GSC lines G157 and G816 were excluded from co‐culture experiments as GFP expression interferes with MPFC.

### 2.20 MHC‐I Blockage Assay

EVs or EVOVs isolated from both mock‐ and oHSV‐1‐infected GSCs (*n* = 3) were subjected to MHC‐I blockade using the HLA‐ABC monoclonal antibody at a concentration of 5 µg/mL. The samples were incubated at 4°C with gentle agitation for 1 h, followed by the priming of freshly isolated PBMCs (*n* = 2) for 3 days. The cells were then processed for MPFC analysis.

### 2.21 Ip Mhc‐I

HLA‐I‐associated peptides were isolated by immunoaffinity purification and acidic elution from bead‐bound HLA‐I complexes as previously described (Purcell et al. 2019, Sturm et al. 2021). Eluted peptides were reduced with 10 mM tris(2‐carboxyethyl)phosphine, alkylated with 30 mM chloroacetamide, and purified by the SP3 protocol using a 1:1 mixture of hydrophilic and hydrophobic Sera‐Mag SpeedBeads. Peptides were bound in 95% acetonitrile containing 0.1% formic acid, washed with 85% ethanol, then acetonitrile, and eluted in water.

Half of each sample was loaded onto Evotip Pure precolumns according to the manufacturer's instructions. LC‐MS/MS was performed using an Evosep One system coupled to an Orbitrap Astral mass spectrometer equipped with a FAIMS Pro interface. Peptides were separated using the 88‐min Evosep method. FAIMS Pro was operated in Standard Resolution mode with compensation voltages of −40 and −60 V. Full MS scans were acquired in the Orbitrap at a resolution of 120,000 over an m/z range of 350–800 with an AGC target of 3 × 10^6^ and a maximum injection time of 100 ms. Precursor ions with charge states 2–4 were isolated using a 2 m/z window and fragmented by HCD with a normalized collision energy of 29%. MS/MS spectra were acquired in the Astral analyzer over an m/z range of 110–1500 using a 0.6‐s cycle time. Dynamic exclusion was set to 15 s.

Raw LC‐MS/MS data were processed using Proteome Discoverer 3.2. MS/MS spectra were searched using Sequest HT with INFERYS Rescoring against a combined database comprising the UniProtKB Homo sapiens canonical and isoform reference proteome (105,274 sequences; release 2025‐09‐08) and the HSV‐1 reference proteome (73 sequences; UniProt proteome UP000009294; release 2025‐04‐26). Searches were performed with no enzyme specificity, a precursor mass tolerance of 8 ppm, a fragment mass tolerance of 0.05 Da, peptide lengths of 7–20 amino acids, and carbamidomethylation of cysteine as a fixed modification. Peptide‐spectrum matches were validated with Percolator using a target‐decoy strategy and filtered at a 1% false discovery rate. Only high‐confidence peptide identifications were retained for downstream analyses.

### 2.22 Bioinformatic Analysis of the EV and EVOV HLA‐I Immunopeptidome

HLA‐I‐associated peptides were compared between matched EV and EVOV samples. Detected peptides were analysed using paired two‐tailed *t*‐tests and log_2_ fold change (EVOV/EV). Peptides with *p* < 0.05 and log_2_ FC >2 were classified as EVOV‐upregulated, whereas peptides with *p* < 0.05 and log_2_ FC <−2 were classified as EVOV‐downregulated. Peptides detected exclusively in EVOV or EV samples were classified as EVOV‐specific or EV‐specific, respectively. Remaining peptides were classified as unchanged.

Peptide source proteins were mapped to unique gene symbols for downstream analyses. Gene Ontology and pathway enrichment analyses were performed separately for the EVOV‐upregulated, EVOV‐downregulated, and unchanged source gene sets using ShinyGO (v0.77, (Ge et al. 2020)) with *Homo sapiens* annotation. The complete HLA‐I immunopeptidome‐derived source gene set was used as the background to account for the inherent functional bias of HLA‐I‐associated proteins. Consequently, enrichment reflects relative differences within the HLA‐I immunopeptidome rather than enrichment against the whole human genome.

Peptide‐source genes were matched to proteins identified in the global EV/EVOV DIA‐MS dataset, following gene‐symbol harmonization, to assess overlap between the HLA‐I immunopeptidome and the whole‐vesicle proteome. Gene symbols were further projected onto the paired PBMC–GBM stem cell organoid single‐cell RNA sequencing dataset to evaluate expression across annotated tumour and immune cell populations and calculate pathway‐specific module scores.

To complement pathway enrichment analysis, source genes were manually curated into functional categories based on UniProt and Gene Ontology annotations, including interferon and antiviral responses, inflammatory and myeloid signalling, antigen processing and proteostasis, oxidative stress and redox regulation, mitochondrial metabolism, vesicle trafficking, RNA processing, and GBM‐associated signalling. Genes represented by peptides assigned to multiple regulation groups were interpreted as evidence of peptide‐level remodelling of the HLA‐I immunopeptidome rather than uniform regulation of the corresponding source proteins.

### 2.23 Extreme Limiting Dilution Analysis (ELDA)

Single‐cell suspensions of GSCs were seeded into ultra‐low attachment 96‐well plates at 1, 10, 25, 50, and 100 cells per well, with 8–26 technical replicates per dose. A total of 1 × 10^4 primed PBMCs, along with PBS, EVs, EVs with MHC‐I blockade, EVOVs, and EVOVs with MHC‐I blockade, were added to each well. The co‐culture was maintained for 8 days. After this period, each well was examined for neurosphere formation. The percentage of wells without neurospheres was calculated for each initial cell density and plotted against the number of seeded cells to assess sphere‐formation efficiency. Sphere formation was monitored using a ZEISS microscope in fluorescence or contrast‐phase mode.

### 2.24 MPI

Organoids were fixed in 4% paraformaldehyde at 4°C for 48 h, transferred to 70% ethanol, and processed through sequential dehydration steps in 96% and 100% ethanol, followed by xylene incubation and paraffin embedding. FFPE blocks were sectioned at 5 µm and mounted onto Superfrost Plus slides. Sections were deparaffinized, and antigen retrieval was performed using the EZ‐Retriever system. Autofluorescence was quenched by UV exposure on a light box.

Slides were blocked using Image‐iT FX Signal Enhancer and stained with a validated antibody panel targeting CD3, CD4, CD8, CD11c, FoxP3, PD‐1, PD‐L1, TBX21, Ki67, HLA‐ABC, and GrB. Coverslips were mounted with ArgoFluor Mounting Medium and cured overnight at room temperature. Imaging was performed using the Orion MPI platform at 20× magnification. Following image acquisition, coverslips were removed, and H&E staining was performed. Slides were rescanned, and all image data were exported for downstream analysis using Rarecyte software.

### 2.25 ScRNA‐seq

Cell fixation was performed using the ScaleBio Sample Fixation Kit according to the manufacturer's protocol. Briefly, cells were washed with ice‐cold PBS and centrifuged at 500 × g for 5 min at 4°C. After resuspension in 500 µL PBS, cells were fixed with 2 mL of freshly prepared Complete Cell Fixation Solution containing 100% methanol and DEPC, which was added dropwise while vortexing to prevent clumping. Fixation proceeded for 15 min on ice. Cells were washed with 5 mL Wash Buffer, centrifuged, and resuspended in residual buffer. Following a second wash and centrifugation, fixed cells were resuspended in 100 µL Wash Buffer, counted, and either used immediately or stored at ‐80°C for up to one month.

scRNA‐seq libraries were prepared using the ScaleBio single‐cell whole‐transcriptome platform (Scale Biosciences, San Diego, CA), which employs a combinatorial indexing strategy based on the Split&Pool method. Cells derived from 6 experimental samples (across two conditions) were distributed into the wells of a 96‐well indexed RT plate, then successively transferred through a 384‐well ligation plate and a final indexed PCR plate. This enabled unique molecular labelling of cDNA per cell and subsequent library generation using standard RNA‐seq workflows. Cells were enzymatically lysed before PCR amplification. Libraries were quantified using both Nanodrop spectrophotometry and the Quantus Fluorometer (Promega) with the QuantiFluor dsDNA System. Library quality was assessed using the Agilent 2100 Bioanalyzer with the High Sensitivity DNA Kit (Agilent Technologies). Final libraries were sequenced on the Illumina NovaSeq 6000 platform (San Diego, CA).

### 2.26 ScRNA‐seq Data Processing and Analysis

Sample demultiplexing, alignment to the GRCh38.p14 human genome, and generation of count matrices were performed using the ScaleRNA pipeline v1.6.2 (https://github.com/ScaleBio/ScaleRna) with default settings, except for the cellFinder option, which was set to ‘true’. The library structure libV1.1 was used.

Downstream analysis was performed in R v4.2.2 using Seurat v5.0.2. Cells with fewer than 200 or more than 12,000 detected features, or more than 5% mitochondrial content, were excluded. Data were normalized using the NormalizeData function (LogNormalize method) and scaled using the ScaleData function. PCA was computed on all variable features, and 22 principal components (PCs) were retained for downstream steps. Data integration was performed using IntegrateLayer with the CCAIntegration method. Cell clustering was performed using FindNeighbors (dimensions = 22) and FindClusters (resolutio*n* = 0.8), and dimensionality reduction was performed using RunUMAP.

Cluster annotation of PBMCs was assessed using ScType, SingleR, and CIBERSORTx, and GSCs clusters were analysed using published GBM marker lists (Neftel et al. 2019), but final labels were determined manually. Cluster markers were identified using FindAllMarkers. Pseudobulk differential expression was computed using AggregateExpression and DESeq2 v1.38.3. Functional enrichment analysis was carried out using enrichPathway() from the ReactomePA (Yu and He 2016) package (v1.42.0). Data visualization used ggplot2 v3.3.6, enrichplot v1.18.4, and scrna‐recom (https://github.com/WXlab‐NJMU/scrna‐recom). Analysis of cell‐to‐cell interaction was performed in R using the cellchat package v. 2.1.2 (https://www.nature.com/articles/s41596‐024‐01045‐4).

### 2.27 ScRNA‐seq Pseudobulk Reproducibility and Effect‐Size Analysis

For scRNA‐seq differential expression analysis, raw gene counts were aggregated at the sample level within each annotated cell subpopulation to generate pseudobulk profiles. Differential expression was then performed on pseudobulk counts, preserving biological replicate structure and avoiding treatment of individual cells from the same sample as independent observations. Replicate‐level expression summaries for selected IFN/MHC‐I‐related genes were visualized across individual biological replicates and GSC subpopulations using aggregated normalized expression values.

To estimate the magnitude of EVOV‐associated transcriptional changes, Cohen's d effect sizes were calculated for significantly altered IFN/MHC‐I‐related genes across annotated GSC subpopulations. Effect sizes were used as standardized descriptive estimates of the direction and magnitude of EVOV–EV transcriptional differences across biological replicates.

### 2.28 ELISA

Plasma collected from mice treated with EV or EVOV was analysed for human IFN‐γ by sandwich ELISA using a high‐binding 96‐well plate and the manufacturer's buffers (kit‐supplied). Plates were coated with capture antibody (2 µg/mL in PBS, 50 µL per well) and incubated overnight at room temperature. Plates were washed 3× with PBS + 0.05% Tween‐20 (≈200 µL/well) and blocked with 150 µL Reagent Diluent for 1 h at RT. Samples were diluted in PBS + 0.1% BSA. A standard curve spanning 0–2400 pg/mL IFN‐γ was prepared by serial dilution according to kit instructions. Samples and standards (50 µL per well) were added and incubated for 1.5 h at RT; plates were then washed 3× as above. Detection antibody (200 ng/mL in Reagent Diluent, 50 µL per well) was applied for 2 h at RT, followed by Streptavidin‐HRP (1:40, 50 µL per well) for 20 min at RT protected from light; plates were washed 3× between steps. Colour was developed with 50 µL per well of substrate solution (1:1 mix of Colour Reagent A and B) for 20 min in the dark, and the reaction was stopped with 25 µL per well of Stop Solution. Absorbance was read immediately at 450 nm with wavelength correction at 540/570 nm.

### 2.29 Spatial Transcriptomics (VisiumHD)

Brains were carefully extracted, rinsed in PBS, and snap‐frozen in methylpentane pre‐cooled in liquid nitrogen. For cryoembedding, Optimal Cutting Temperature (OCT) was added to the labelled cryomolds (at 1/3 volume). Once the OCT was partially frozen on dry ice, the brain was properly positioned and covered with additional OCT. The spleen was also collected and processed for downstream analysis. Frozen tissues were stored at ‐80°C until further processing.

Tissue samples were collected from *n* = 4 fresh‐frozen (FF) mouse brains, representing two experimental groups: EV and EVOV. Cryosections (10 µm thick) were prepared using a Leica CM1860 UV cryostat and mounted on Superfrost Plus slides for quality control, imaging, and section selection. Haematoxylin and eosin (H&E) staining and imaging were performed in accordance with the VisiumHD FF Tissue Preparation Handbook (CG000763). Selected samples were processed and sequenced in accordance with the VisiumHD Spatial Gene Expression Reagent Kits User Guide (CG000685). Libraries were sequenced on an Illumina NovaSeq X Plus system using a 1.5B flow cell, with a 43‐10‐10‐50 nucleotide configuration (Read 1 ‐ i7 ‐ i5 ‐ Read 2) and a 1% PhiX spike‐in, in accordance with the VisiumHD protocol.

### 2.30 Spatial Transcriptomics (VisiumHD) Data Processing and Analysis

Raw FASTQ files from VisiumHD libraries were processed using Space Ranger v3.0 (10x Genomics) with alignment to the human GRCh38 reference genome. In addition to the native 2 µm feature size, Space Ranger generated spatially binned datasets at 8 and 16 µm resolution. Unless otherwise stated, downstream analyses used 8 µm binning, which yields ∼16‐fold higher UMI counts per bin than 2 µm features while maintaining near single‐cell resolution and compatibility with third‐party tools (VisiumHD Spatial Gene Expression Performance Technical Note, CG000686, revision A). T cell spatial domains (core, border, and periphery) were defined using histomorphological context, complemented by spatial gradients in transcript capture (mean nCount/UMI) and continuity of the malignant signal, and served as the primary stratification framework for downstream spatial comparisons.

Data were imported into Seurat v5.0.2 (R v4.3.0) for preprocessing. Spots with fewer than 70 UMIs were excluded. For each section, expression matrices were processed with NormalizeData, FindVariableFeatures, ScaleData, RunPCA, RunUMAP, FindNeighbours, and FindClusters. Mouse cells formed discrete clusters within the first eight PCs and were removed. Plasma cells were clearly separated in each sample. T cells, which clustered poorly, were isolated based on canonical markers (*CD3D, LCK, CD4, CD8A, PRF1, GZMB, GZMK, GZMA, FOXP3, IL2RA*). In the humanized mouse model, no other immune lineages were detected; the remaining cells were annotated as tumour. Given the sparse and dropout‐prone nature of spot‐ and bin‐level VisiumHD expression profiles and the close transcriptional similarity among functional T cell states, T cell analyses were structured around anatomical domains rather than de novo functional subclustering, and functional phenotypes were quantified using predefined marker programs/module scores. To prioritize platform‐native, reproducible inference, immune readouts were derived from direct marker evidence and gene program scoring within spatial domains, rather than reference‐dependent deconvolution approaches that introduce additional modelling assumptions and parameter sensitivity.

For tumour, plasma, and T cell datasets, UMI count matrices from four sections were merged. To reduce computational load, sketch‐based analysis (https://satijalab.org/seurat/articles/seurat5_sketch_analysis) using leverage‐score sketching was applied to a 15% cell sample to preserve rare populations. Integration was performed using reciprocal PCA (rPCA) with 8 PCs for tumour, 6 PCs for T cells, and 4 PCs for plasma cells (k.anchor = 40). Variable features were identified, data were scaled, PCA was rerun, and clustering was performed using the same number of PCs as in integration, with resolutions of 0.3 (tumour), 0.4 (T cells), and 0.1 (plasma cells).

Differentially expressed genes (DEGs) were identified using Seurat's Wilcoxon rank‐sum test (adjusted p‐value ≤ 0.05). For gene set enrichment analysis (GSEA), DEGs were identified with Presto, ranked by AUC, and analysed with fgsea against the MSigDB Gene Ontology Biological Process and Molecular Function collections, with significance defined as an adjusted p‐value ≤ 0.1. VisiumHD analyses were performed on *n* = 2 biological replicates per condition and are therefore presented as exploratory, hypothesis‐generating spatial case studies. Spot/bin‐level statistical tests were used for descriptive feature prioritization and spatial mapping; because spot‐level sampling can inflate statistical significance due to pseudo‐replication, treatment‐associated patterns were interpreted primarily based on effect sizes and concordant directionality across biological replicates, and statistical significance derived from spot‐level testing was interpreted cautiously, with VisiumHD results presented as supporting spatial evidence rather than standalone inferential claims.

Cell cycle phase was inferred using CellCycleScoring, and proliferative states were annotated as quiescent (<10% PCNA/MCM/CDC20/TOP2A‐positive), G1/S, G2/M, or mixed. Additional gene signature scores were calculated using Seurat's AddModuleScore function. Exhaustion was scored using *PDCD1, LAG3, HAVCR2*, and *TIGIT*, and hypoxia was scored using *ENO1, ENO2, DDIT3, PLOD2, PIM1, HIF1A‐AS2, HIF1AN, HIF1A, VEGFA, SLC2A1, ADM, NDRG1, HK2*, and *PFKP*. The T cell cytotoxicity score was defined as a composite transcriptional score based on canonical cytotoxic effector genes *PRF1, GZMB, GNLY, NKG7*, and *IFNG*. For each annotated T cell spatial domain, gene expression values were scaled and centered, and the final cytotoxicity score was calculated as the average z‐score across the five‐gene signature. Malignant cluster annotation followed a three‐criterion framework: (1) lineage state scoring (NPC, OPC, AC, MES1, MES2) from the Neftel atlas (Neftel et al. 2019) using AddModuleScore; (2) spatial compartment (tumour‐stroma‐ border vs hypoxic core) inferred from histopathology and mean nCount; and (3) dominant cell‐cycle phase. This yielded five malignant clusters with distinct anatomical niches and transcriptional programs:

MES1_border ‐ECM‐remodelling/angiogenic‐edge program (*COL6A3, SERPINE1, LOXL1, FBLN1, IGFBP4, IL11*)

MES1_border_ANG—angiogenic/transitional (*PROCR, NR4A2, COL8A1, SYNE1*)

MES1_border_IFN—ISG‐high (*GBP5, GBP4, GBP1, CD74, IRF1, TNFRSF14*)

MES2_core—hypoxic/glycolytic (*VEGFA, HK2, SLC2A1, HMOX1, DDIT4, ADM*)

MES2_core_hypoxic—stress/glycolytic hypoxia (*PFKFB4, NDRG1, HILPDA, BNIP3L, DEPP1, ALDOC*)

### 2.31 Spatial Compartment Quantification, Pseudobulk Reproducibility, and Effect‐size Analysis

Annotated tumour‐cell states and T‐cell spatial regions were summarized as within‐sample proportions for each biological replicate. Because the analysed sections were not anatomically identical, absolute bin counts were not compared between samples. EVOV–EV differences were summarized as replicate‐level percentage‐point contrasts in within‐sample proportions. To assess reproducibility across biological replicates, raw spatial UMI counts were aggregated into sample‐level pseudobulk profiles for each annotated compartment, then normalized to library size and log‐transformed. Replicate concordance was evaluated within treatment groups by comparing pseudobulk expression profiles between biological replicates using Pearson correlation. For selected spatial module scores, values were first averaged within each biological replicate and annotated compartment. Standardized effect sizes were then calculated using Hedges’ g, which incorporates a small‐sample correction and provides a descriptive estimate of EVOV‐associated effects. Because VisiumHD profiling was performed with two biological replicates per condition, effect‐size estimates were interpreted descriptively rather than as formal inferential statistics.

### 2.32 Immunofluorescence Staining and Confocal Microscopy

Brains were embedded in OCT, cryosectioned at 10 µm thickness, and mounted onto glass slides. Sections were fixed with 4% paraformaldehyde for 20 min at room temperature, permeabilized and blocked in PBS containing 0.5% Triton X‐100, 10% normal goat serum, and 5% BSA for 1 h. Sections were incubated with primary antibodies overnight at 4°c, followed by Alexa Fluor 555‐conjugated secondary antibodies (1:500) for 1 h at room temperature in the dark. Double immunofluorescence staining was performed using the following antibody combinations: Ki67/CD45, cCASP3/MHC‐I, CD45/MHC‐I, and Granzyme B (GrB)/CD45. Images were acquired using a Zeiss LSM 780 confocal microscope (Axio Observer Z1) and analysed using Zeiss ZEN 3.7 software.

Images were quantified using a semi‐automated object‐based analysis of separated fluorescence channels. One field per section was divided into four quadrants, and double‐positive objects were expressed per 100 denominator‐positive objects. Statistical comparisons were performed on animal‐level means, obtained by averaging four quadrants per animal, using two‐sided Mann–Whitney tests.

### 2.33 Clinical Bulk RNA‐seq Cohorts and Survival Modelling

Bulk RNA‐seq datasets From three human GBM cohorts (TCGA‐GBM, CGGA, and CAN‐3110 Arm A/B) were analysed to assess survival associations of Reactome pathway signatures upregulated in EVOV‐primed GSC–PBMC organoids by scRNA‐seq. For TCGA (*n* = 239; IDH‐WT, WHO grade IV), TPM expression and clinical data were downloaded From the GDC portal and restricted to protein‐coding genes; batch effects driven by sample processing site were corrected using sva (v3.58.0) while preserving age, sex, WHO grade, IDH status, and MGMT promoter methylation status. The analysis was performed for the whole TCGA‐GBM cohort and After stratification by transcriptional subtype, including MES, classical, proneural, and neural tumours. For CGGA (*n* = 104; IDH‐WT, WHO grade IV), raw counts and clinical data were downloaded, restricted to protein‐coding genes, and converted to TPM. For CAN‐3110 Arm A/B (Ling et al. 2025), protein‐coding TPM matrices were batch‐corrected by sequencing group After PCA assessment and restricted to IDH‐WT, WHO grade IV samples (14 pre‐treatment, 16 post‐treatment). Reactome pathways significantly upregulated in EVOV organoids were converted Into ssGSEA signatures by extracting pathway gene sets and computing per‐sample signature scores using the ssGSEA approach described for CAN‐3110. Survival analyses were performed in R (v4.4.3) using survival (v3.8.3), survminer (v0.5.1), ReactomePA (v1.50.0), and ggplot2 (v4.4.0). In TCGA and CGGA, multivariable Cox proportional hazards models tested associations Between signature scores and overall survival, adjusting for age, sex, and MGMT promoter methylation status. In CAN‐3110, univariate Cox models were used due to cohort size; HRs, 95% CI, and p‐values were summarized in forest plots for pre‐ and post‐treatment samples, including all IDH‐WT Arm A/B patients (*n* = 30) and the HSV1‐seropositive post‐treatment subset (*n* = 24). Multiple testing correction was performed using Benjamini–Hochberg FDR.

### 2.34 Quantification and Statistical Analysis

All experiments used randomized allocation; investigators performing data acquisition and analysis were blinded to group assignments. Independent blinded investigators performed downstream measurements where applicable.

All statistical analyses were performed using GraphPad Prism v9.0 (GraphPad Software RRID: SCR_002798) and R v4.3.0. Unless otherwise stated, all tests were two‐tailed with α = 0.05. Assumptions of normality and homogeneity of variance were checked before applying parametric tests. For each test, exact p‐values, test statistics, degrees of freedom, and effect sizes (Cohen's d or log_2_ fold change with 95% CI) were calculated where appropriate. PCA was performed using ClustVis (Metsalu and Vilo 2015). Hierarchical clustering (1‐ Pearson correlation) analysis of gene expression was performed using the Morpheus matrix visualization and analysis software (https://software.broadinstitute.org/morpheus).

Paired, two‐tailed *t*‐tests were used when comparing matched conditions (e.g., EV vs. EVOV from the same donor). Unpaired *t*‐tests (two‐tailed) were applied when comparing independent groups. One‐tailed paired *t*‐tests were used in scRNA‐seq comparisons when directional hypotheses were tested (Figure 5C). Data are presented as individual values with means ± standard error of the mean (SEM). Statistical significance is denoted as follows: (*p ≤ 0.05; **p ≤ 0.01; ***p ≤ 0.001).

ScRNA‐seq data were processed using Seurat v5.0.2. Cells with fewer than 200 or more than 12,000 detected genes, or with more than 5% mitochondrial content, were excluded. Data were normalized using the LogNormalize method, and PCA was computed on the resulting variables. Clustering was performed using the CCAIntegration method, FindNeighbors, and FindClusters (resolution = 0.8), followed by dimensionality reduction with RunUMAP. The DEGs were identified using the pseudobulk approach with the AggregateExpression function. Differential expression in scRNA‐seq pseudobulk analysis was performed using DESeq2 v1.38.3 with the Wald test (two‐sided) and Benjamini–Hochberg correction (FDR ≤ 0.05). Pathway enrichment analysis was performed with ReactomePA and clusterProfiler. Cell types were annotated based on ScType, SingleR, CIBERSORTx, and established GBM marker references (Neftel et al. 2019).

Clinical bulk RNA‐seq survival analysis. Cox proportional hazards regression was used to test associations between ssGSEA signature scores derived from EVOV‐primed GSC–PBMC organoid scRNA‐seq Reactome pathways and survival in TCGA (*n* = 239) and CGGA (*n* = 104) using multivariable models (age, sex, MGMT promoter methylation status), and in CAN‐3110 using univariate models due to sample size (14 pre‐treatment, 16 post‐treatment). Analyses were conducted in R v4.4.3 (survival v3.8.3; survminer v0.5.1; ReactomePA v1.50.0; ggplot2 v4.4.0). P‐values were adjusted using Benjamini–Hochberg FDR.

Proteomic data from Ultra‐Deep DIA‐MS were analysed using Spectronaut v18.3 or v18.4 (Biognosys AG) with the directDIA+ pipeline. Searches were performed against the UniProt human proteome and oHSV‐1 proteome, allowing for variable modifications and two missed cleavages. Peptide‐ and protein‐level FDR were set at 1%. Group comparisons were based on log_2_‐transformed intensity values and assessed using paired *t*‐tests or empirical Bayes moderated *t*‐tests as implemented in limma. Multiple testing correction was performed using Benjamini–Hochberg FDR. Functional pathway enrichment was performed using ReactomePA and clusterProfiler. Stromal and immune scores were derived from significantly deregulated proteins using established scoring algorithms, and proteomic immune cell‐type deconvolution was performed using curated marker sets.

Immunopeptidomic analysis was based on DIA_MS data matched against the HLA Ligand Atlas, IEDB, and MHC Motif Atlas. Peptide‐spectrum matches were filtered at 1% FDR. Differentially expressed immune peptides were identified using two‐tailed paired *t*‐tests (FC > 1.5, *p ≤ 0.05), and their source genes were annotated using Gene Ontology and ReactomePA. Immunogenicity scores were assigned based on the IEDB MHC I immunogenicity tool.

To ensure the inclusion of proteins or immune peptides detected exclusively in one condition (e.g., EV or EVOV), a value of 1 was added to all raw intensity values before calculating fold changes. This transformation enabled the computation and visualization of log_2_ fold changes for these features in volcano plots and heatmaps presented in the supplementary data. This approach is commonly applied in proteomic data processing to handle zero‐intensity values in comparative analyses.

Statistical comparisons of immune cluster abundance across MPFCs were performed using linear mixed‐effects models (LMMs) implemented in the lme4 package (version 1.1‐35.5) (Douglas Bates et al. 2015). Stimulation was included as a fixed effect, and random intercepts for cell line nested within sample donor were added to account for inter‐patient and inter‐cell‐line variability. Pairwise comparisons between stimulation levels were performed using estimated marginal means (EMMs) from the emmeans package (version 1.10.5), with Tukey's adjustment for multiple comparisons.

Cytokine concentrations were quantified using the LEGENDplex Human Essential Immune Response Panel and analysed by paired two‐tailed *t*‐tests.

Multiplex immunofluorescence imaging was analysed using CyteFinder (Rarecyte). Infiltration of CD8^+^GrB^+^ and CD11c^+^PD1^+^ cells was quantified based on marker co‐localization.

For tumour BLI, Two‐way ANOVA was performed using a mixed‐effects model. The model included fixed effects for group, time, and their interaction, with matched values treated as repeated measures. Tukey's post hoc test was applied for multiple comparisons.

Spatial transcriptomics data were processed using Space Ranger (10x Genomics) and analysed in Seurat v5.0.2, with functional enrichment of spatial clusters performed using ReactomePA. All statistical analyses on spatial transcriptomic data were performed in R v4.3.0 or GraphPad Prism v9.0. For matched comparisons (e.g., EV vs EVOV from the same donor), paired *t*‐tests were applied; for independent groups, unpaired *t*‐tests were used. When multiple comparisons were performed, Tukey's or Benjamini‐Hochberg correction was applied.

For spatial transcriptomics, Differential expression between treatment and control was tested per population using Seurat's Wilcoxon rank‐sum test, retaining genes with padj ≤ 0.05. For GSEA, differential expression was computed with Presto, ranked by AUC, and analysed with fgsea against MSigDB Gene Ontology Biological Process and Molecular Function collections. Gene sets with padj ≤ 0.1 were considered significant. Functional enrichment used ReactomePA and clusterProfiler. Volcano plots were generated in ggplot2, defining upregulated genes as adjusted *p* < 0.05 and log_2_FC > 0.25, and downregulated genes as log_2_FC < ‐0.25. Heatmaps were produced using DoHeatmap after downsampling to 2000 cells per population.

Gene signature scores were computed using AddModuleScore (Seurat) and standardized as z‐scores where appropriate. Statistical tests for signature differences between groups used Wilcoxon rank‐sum (two‐sided) or GLMMs (for repeated measures), with p‐value correction for multiple testing.

A detailed description of the reagents used in the study is provided in the Supplementary Methods file.

## 3 Results

### 3.1 EVOVs Reprogram PBMC Composition toward Cytotoxic and Proinflammatory Immune Phenotypes

To first evaluate the *ex vivo* immunomodulatory effect of EVOVs on immune cells, PBMCs were primed with EV or EVOV generated from mock‐ or oHSV‐1‐infected GSCs, respectively. Before PBMC priming, EVs and EVOVs were characterized and quantified, showing enrichment of EV‐associated markers and comparable particle size and concentration between EV and EVOV samples (Figure S1A–D). PBMCs were then exposed to EVs or EVOVs for 72 h and analysed by MPFC using a 17‐marker panel after gating on viable CD45^+^ immune cells (Figures 1A and S1E). Batch consistency across independent acquisition days and reference UMAP distributions for PBS‐treated CD3^+^ and CD3^−^ PBMC compartments were used to support the reproducibility of the MPFC workflow and cluster annotation (Figure S1F,G).

**FIGURE 1:**
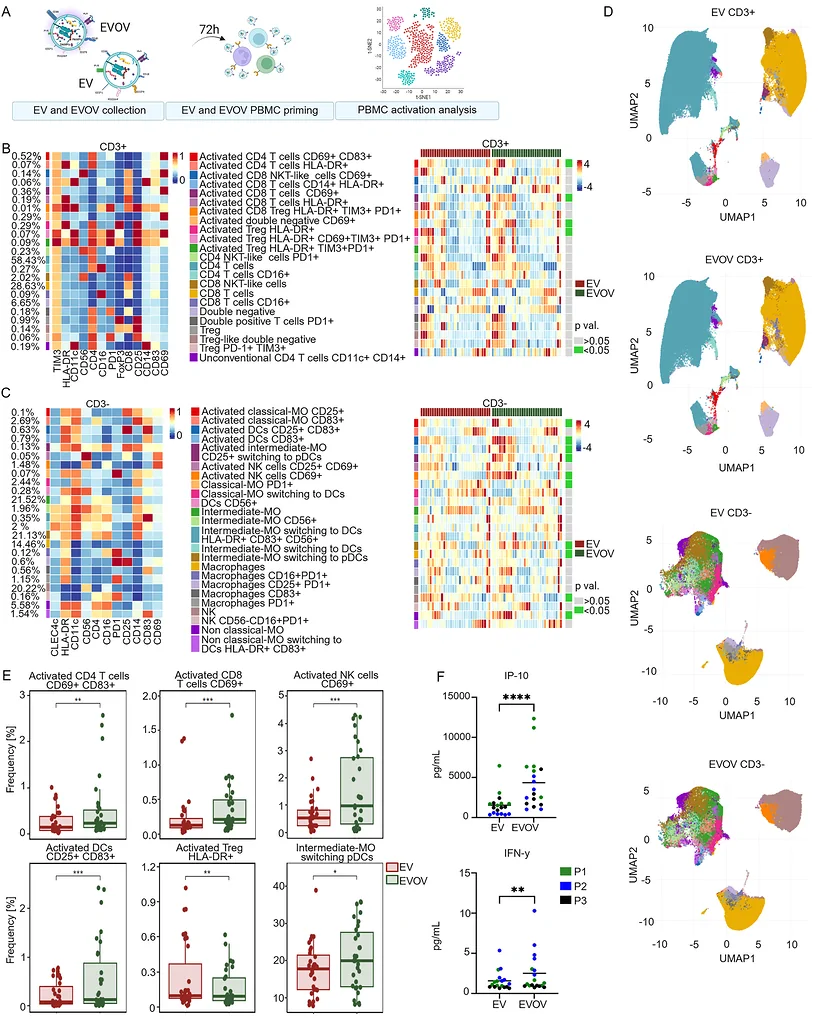
Profiling of EV‐ or EVOV‐primed PBMCs. (A) Schematic overview of PBMC priming and MPFC analysis. (B) Heatmaps of relative identity and percentage (left) of differential abundance (right) of CD3^+^ cell clusters of PBMC (*n* = 8) primed with EV or EVOV (*n* = 6) by MPFC, mixed‐effects model with Tukey correction. (C) Heatmaps of relative identity and percentage (left) of differential abundance (right) of CD3^−^ cell clusters of PBMC (*n* = 8) primed with EV or EVOV (*n* = 6) by MPFC, mixed‐effects model with Tukey correction. (D) UMAP projections of CD3^+^ (top) and CD3^−^ (bottom) cell clusters of PBMC (*n* = 8) primed with EV or EVOV (*n* = 6) by MPFC. (E) Bar plots of CD3^+^ and CD3^−^ cell clusters of PBMC (*n* = 8) primed with EV or EVOV (*n* = 6) by MPFC, mixed‐effects model with Tukey correction, *p ≤ 0.05; **p ≤ 0.01; ***p ≤ 0.001. (F) Scatter plots of cytokine profiles in supernatants of PBMCs (*n* = 3) primed with EV or EVOV (*n* = 6) by MPFC, paired *t*‐test, *p ≤ 0.05; **p ≤ 0.01.

A total of 49 immune subpopulations, comprising 23 CD3^+^ T/NKT cell subsets and 26 CD3^−^ innate immune cell types, were defined using FlowSOM clustering and analysed across six healthy donors. Differential abundance and population frequencies revealed that EVOV priming significantly reshaped the immune landscape of both CD3^+^ and CD3^−^ compartments (Figure 1B,C). EVOVs increased the frequency of activated cytotoxic subsets, including activated CD4 T cells (CD69^+^CD83^+^), CD8 T cells (CD69^+^), and NK cells (CD69^+^), alongside dendritic cells (DCs CD25^+^CD83^+^). Although the total NK cell frequency was reduced, this likely reflected redistribution within the CD3^−^ compartment toward activated NK CD69^+^ and other EVOV‐responsive DC/monocyte‐like populations rather than selective NK cell depletion. In contrast, Tregs were not expanded, indicating that immune cells were selectively activated (Figures 1D,E, S2A and S3A).

To assess functional cytokine output, culture supernatants were analysed using a LEGENDplex cytokine assay. EVOV‐primed PBMCs secreted significantly higher levels of proinflammatory mediators, including Interferon‐gamma (IFN‐γ) and interferon‐gamma‐inducible protein 10 (IP‐10), compared to EV‐primed counterparts (Figures 1F and S2B). These cytokine signatures are consistent with a type I immune activation profile linked to antitumour responses.

To assess whether increased EVOV exposure induced overt PBMC toxicity or nonspecific immune hyperactivation, PBMC viability and immune composition were evaluated after EV/EVOV priming in monoculture using the standard dose and a 2.5‐fold higher dose (Figure S4). Live CD45^+^ PBMC frequencies remained high across the tested conditions, with no significant reduction between standard‐dose and high‐dose EVOV exposure (Figure S4A). Heatmap and stacked‐bar analyses of CD3^+^ and CD3^−^ immune compartments did not reveal a global dose‐dependent collapse of PBMC populations (Figure S4B–E). Importantly, activation‐associated PBMC populations were not markedly increased in the high‐dose EVOV condition compared with the selected EVOV dose, indicating that higher EVOV exposure did not trigger global immune activation changes, except for an elevation of the activated classical‐MO CD25^+^ subpopulation. High‐dose EV shows elevated non‐classical MO and decreased CD56+ DC subpopulations.

Within the CD3^−^ compartment, a phenotypic switch was observed from intermediate monocytes (MO) toward plasmacytoid dendritic cells (pDCs), further supporting EVOV‐mediated immune reprogramming (Figures 1E and S3A).

Altogether, these findings suggest that EVOVs alter the composition and activation state of PBMCs, promoting the activation of cytotoxic cells and APCs, with no evidence of increased immunosuppression. The dose‐escalation analysis further showed that the selected EVOV dose range preserved PBMC viability and did not induce dose‐dependent nonspecific hyperactivation. This immune reprogramming is thus associated with the enhanced release of proinflammatory cytokines, highlighting the immunostimulatory potential of EVOVs.

### 3.2 EVOV‐Primed PBMCs Enhance Cytotoxic Immune Infiltration in 3D Tumour Organoids

An allogeneic co‐culturing organoid assay was developed to assess the functional impact of EVOV‐primed PBMCs on GSCs infiltration and cancer‐immune interactions (Figure 2A).

**FIGURE 2:**
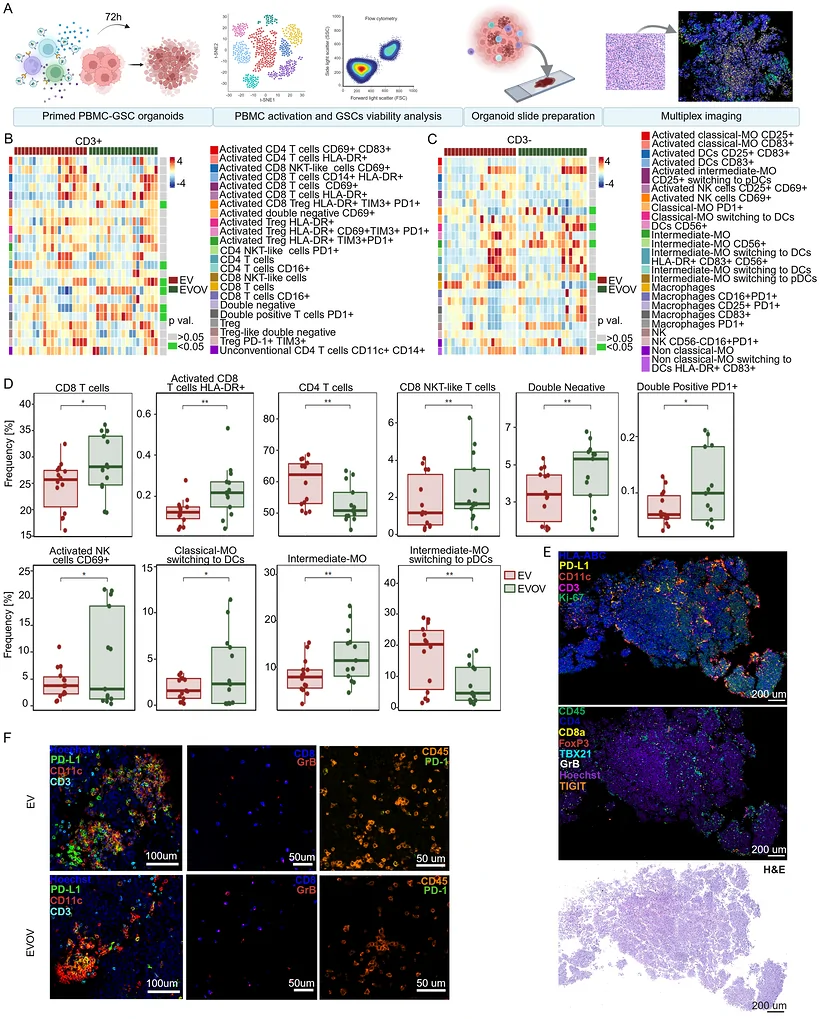
Characterization of GSC‐PBMC organoids. (A) Schematic overview of the organoid analysis by MPFC and MPI. (B) Heatmap of differential abundance of CD3^+^ cell clusters (*n* = 4) derived from organoids with PBMC primed with EV or EVOV (*n* = 4) by MPFC. (C) Heatmap of differential abundance of CD3^−^ cell clusters (*n* = 4) derived from organoids with PBMC primed with EV or EVOV (*n* = 4) by MPFC, mixed‐effects model with Tukey correction. (D) Bar plots of CD3^+^ and CD3^−^ cell clusters (*n* = 4) derived from organoids with PBMC primed with EV or EVOV (*n* = 4) by MPFC, mixed‐effects model with Tukey correction *p ≤ 0.05; **p ≤ 0.01. (E) Representative immunofluorescence with indicated antibodies and H&E staining images of organoids with PBMC primed with EVOV by MPI, scale bar = 200 µm. (F) Representative immunofluorescence images with indicated antibodies of organoids with PBMC primed with EV or EVOV by MPI, scale bar = 50 µm.

MPFC analysis of dissociated organoids revealed that PBMCs primed with EVOVs acquired a more activated and cytotoxic phenotype than those primed with EVs alone. Differential abundance in CD3^+^ and CD3^−^ subpopulations demonstrated significant enrichment of CD8^+^ T cells, CD8^+^ NKT cells, and activated NK cells, along with an MO‐to‐DC transition in the EVOV‐primed group (Figure 2B,C). Analysis of immune subset frequencies confirmed these shifts and revealed a selective expansion of effector populations (Figure 2D) without an increase in regulatory cells (Figures S5A and S6A).

Immunophenotyping of organoids by a 13‐marker MPI (Figures 2E and S7A,B) supported these findings, showing greater infiltration of CD8^+^ GrB^+^ cytotoxic T cells in the EVOV‐primed group, alongside a reduction in immunosuppressive populations, including PD‐L1^+^CD11c^+^ cells and PD‐1^+^CD45^+^ lymphocytes (Figures 2F, S5B and S6B).

Together, these results show that EVOV priming potentiates PBMC cytotoxicity and drives immunological remodelling within organoids by enhancing infiltration of effector lymphocytes and reducing suppressive signalling, thus supporting the pro‐immunogenic capacity of EVOVs in a physiologically relevant multi‐cell‐subpopulation organoid model.

### 3.3 EVOVs Reprogram Cancer and Immune Compartments in GSC‐PBMC Organoids at Single‐Cell Resolution

ScRNA‐seq was performed on GSC‐PBMC organoids to delineate the effects of EVOVs on cancer‐immune interactions at high resolution, upon dissociation and transcriptomic profiling (Figure 3A).

**FIGURE 3:**
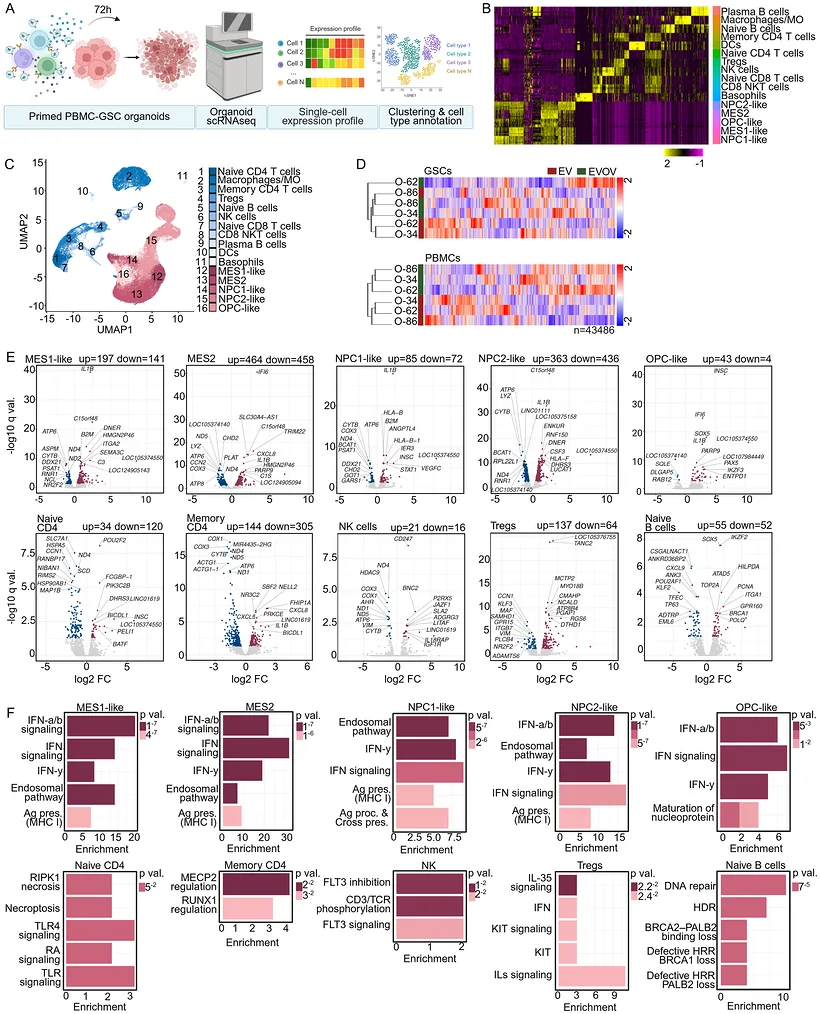
Single‐cell characterization of organoids. (A) Schematic overview of organoid analysis by scRNA‐seq. (B) Heatmap of the top 50 differentially expressed marker genes across annotated clusters in organoids with PBMC primed with EV or EVOV (*n* = 3) by scRNA‐seq, aggregated expression (pseudobulk), Wilcoxon Rank Sum test, FDR≤0.05. (C) UMAP projection of cell subpopulation clusters in organoids with PBMC primed with EV or EVOV (*n* = 3) by scRNA‐seq, paired *t*‐test, one‐tailed, *p ≤ 0.05. (D) Heatmap and hierarchical clustering of GSCs (top) and PBMCs (bottom) from organoids (O) with PBMC primed with EV or EVOV (*n* = 3) (O) by scRNA‐seq, aggregated expression of all genes shown as z‐score. (E) Volcano plots of differentially expressed genes in cell subpopulation from organoids with PBMC primed with EV or EVOV (*n* = 3) by scRNA‐seq, aggregated expression, DESeq2, Wald test, FDR≤0.05. (F) Bar plots of significantly enriched upregulated pathways in cell subpopulation from organoids with PBMC primed with EV or EVOV (*n* = 3) by scRNA‐seq, aggregated expression, hypergeometric test, FDR≤0.05.

Unsupervised annotation followed by marker‐guided annotation identified 16 transcriptionally distinct subpopulations spanning both cancer states (NPC1, NPC2, MES1, MES2, OPC) and immune compartments (CD8^+^ T cells, naïve and memory CD4^+^ T cells, Tregs, NK cells, CD8^+^ NKT cells, DCs, macrophages/MO, Plasma B cells, basophils). Top DEGs robustly distinguished these clusters and separated immune and tumour compartments within the organoids (Figure 3B,C).

The global transcriptomic analysis of GSCs revealed patient‐specific signatures that differed significantly depending on whether the GSCs were derived from organoids primed with EVs or EVOVs. In particular, the transcriptome of PBMCs in organoids was strictly determined by the priming conditions, but not by the GSCs' origin/heterogeneity (Figure 3D).

Subpopulation‐specific differential expression analysis in GSCs from the EVOV organoid group revealed broad and significant upregulation of key components of IFN signalling, including STAT1, IRF1/IRF7, GBPs, TRIM family members, as well as core mediators of MHC‐I antigen processing and presentation, such as TAP1, B2M, and multiple Human Leukocyte Antigen (HLA) isoforms. In parallel, genes associated with tumour‐supportive metabolic and biosynthetic programs were consistently downregulated, including those involved in global protein translation (e.g., RPL22L1, EIF3, EIF4) and cholesterol biosynthesis (especially in OPC‐like subpopulations). These transcriptional shifts were reproducible across all GSC subpopulations and were accompanied by strong enrichment of immune activation and antigen presentation pathways (Figures 3E,F and S8A).

Within PBMC subpopulations from the EVOV organoid group, the most upregulated genes were associated with cytotoxicity (Figure 3E,F). In particular, primary innate immune system sensors, TLRs, were upregulated in naïve CD4 T cells. Additionally, several immune activation pathways, including RIPK1, retinoic acid signalling, MECP2, and RUNX1 regulatory programs in CD4^+^ T cells, and FLT3 and CD3/TCR phosphorylation in NK cells, were selectively enriched, highlighting broad pro‐inflammatory remodelling of lymphoid compartments (Figure 3F).

Mechanistically, observed downregulation of TCR phosphorylation in memory CD4^+^ T cells suggests they receive weaker signals through the T‐cell receptor, making them less responsive to second direct antigen stimulation within the organoids. Because RUNX1‐ and MECP2‐dependent pathways are simultaneously upregulated, this suggests that memory CD4+ T cells compensate by relying more on cytokine‐driven and transcription‐factor–mediated activation (Bangs et al. 2009) rather than classical TCR signalling.

Conversely, downregulated pathways were associated with protein PTMs and chaperone‐mediated autophagy (CMA), along with reductions in toxin‐uptake modules in Tregs and in suppressed IGF‐transport and L1CAM‐interaction modules in naïve CD4^+^ T cells (Figure S8B,C). Together, these downregulated pathways suggest that EVOV priming reduces inhibitory signals and shifts PBMCs toward a more activated, pro‐inflammatory state.

Cohen's d effect‐size analysis further supported the magnitude and directionality of EVOV‐associated transcriptional changes across GSC subpopulations (Figure S8D). To further support the robustness of these findings, replicate‐level visualization of IFN/MHC‐I‐related gene expression across independent GSC‐PBMC organoids showed consistent EVOV‐associated induction of antigen‐presentation and IFN‐response programs across donor‐derived GSC backgrounds (Figure S8A,E–G).

Collectively, these findings demonstrate that EVOVs profoundly alter the transcriptional state of GSC‐PBMC organoids. This reprogramming was characterized by the coordinated activation of IFN/MHC‐I antigen‐presentation programs in GSCs and the enrichment of cytotoxic and inflammatory signalling signatures across PBMC compartments. The added replicate‐level expression summaries and Cohen's d effect‐size analysis provided additional quantitative support for the robustness of EVOV‐associated transcriptional remodelling in the organoid scRNA‐seq dataset.

### 3.4 Systemic Immune Response to EVOV in PBMC‐engrafted NSG Mice with Intracranial GSCs Xenograft

A PBMC‐engrafted NSG mouse model of intracranial GBM was applied to evaluate the systemic and local immune effects of EVOV adjuvant *in vivo*. EV and EVOV were isolated from mock‐ or oHSV‐1‐infected GSCs, characterized, and quantified on the day of injection (Figure S1A–D).

Mice were intravenously injected with human PBMCs (day 0), intracranially implanted with patient‐derived GSCs (G62; day 1), and treated intraperitoneally with freshly isolated EVs or EVOVs (day 2). On day 15, mice were sacrificed to assess immune activation and tumour composition (Figure 4A).

**FIGURE 4:**
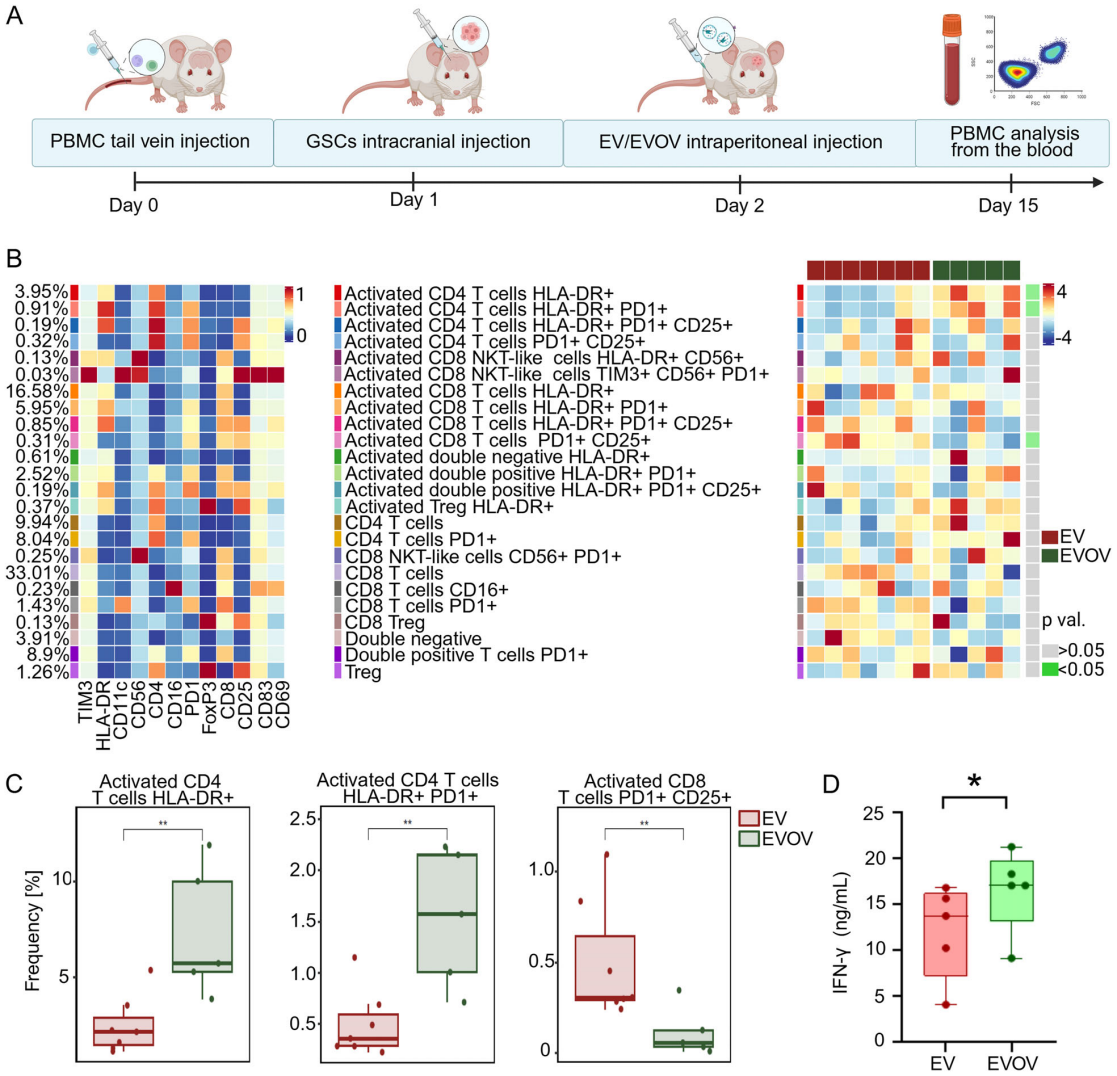
Profiling of systemic immune cells in the NSG model. (A) Schematic overview of the *in vivo* experiment. (B) Heatmaps of percentage (left) and differential abundance (right) of CD3^+^ cell clusters of PBMC from the blood of mice treated with EV (*n* = 7) or EVOV (*n* = 5) by MPFC, mixed‐effects model with Tukey correction, *p ≤ 0.05. (C) Bar plots of CD3^+^ cell clusters of PBMC from the blood of mice treated with EV (*n* = 7) or EVOV (*n* = 5) by MPFC, mixed‐effects model with Tukey correction, *p ≤ 0.05. (D) Bar plot of IFN‐γ concentration in plasma of mice treated with EV or EVOV (*n* = 5) by ELISA, paired *t*‐test, *p ≤ 0.05.

MPFC analysis of peripheral blood (day 15) using a 17‐marker panel, with gating on CD45+ immune cells, was performed for deep immunophenotyping (Figure S9A). Data showed that EVOV‐treated mice exhibited significantly higher frequencies of activated CD4^+^ helper T cells and a reduced proportion of exhausted CD8^+^PD1^+^CD25^+^ T cells, indicating enhanced systemic immune activation (Figures 4B,C and S9A). IFN‐γ was significantly elevated in the plasma of EVOV‐treated animals compared to the EV group (Figure 4D).

Establishing a PBMC‐engrafted mouse model by intravenous injection of PBMCs followed by intracranial implantation of GSCs enabled assessment of a therapeutic window for subsequent intraperitoneal vesicle administration. Importantly, immune cells responded systemically to EVOV treatment, leading to T cell activation and elevated circulating IFN‐γ.

### 3.5 Immune Cell Recruitment into the Brain in PBMC‐engrafted NSG Mice with Intracranial GSCs Xenografts

Exploratory analysis of spatial transcriptomic profiling of brain tissue from intracranial GSC xenografts (Figure 5A) identified spatially organized T cell programs across anatomically defined niches (Figure 5B). These T cell subclusters were annotated into periphery T cells, border T cells, and core T cells. Border and core T cells displayed similar transcriptomic profiles consistent with tumour contact, while periphery T cells showed a distinct signature (Figures 5B,C and S10A).

**FIGURE 5:**
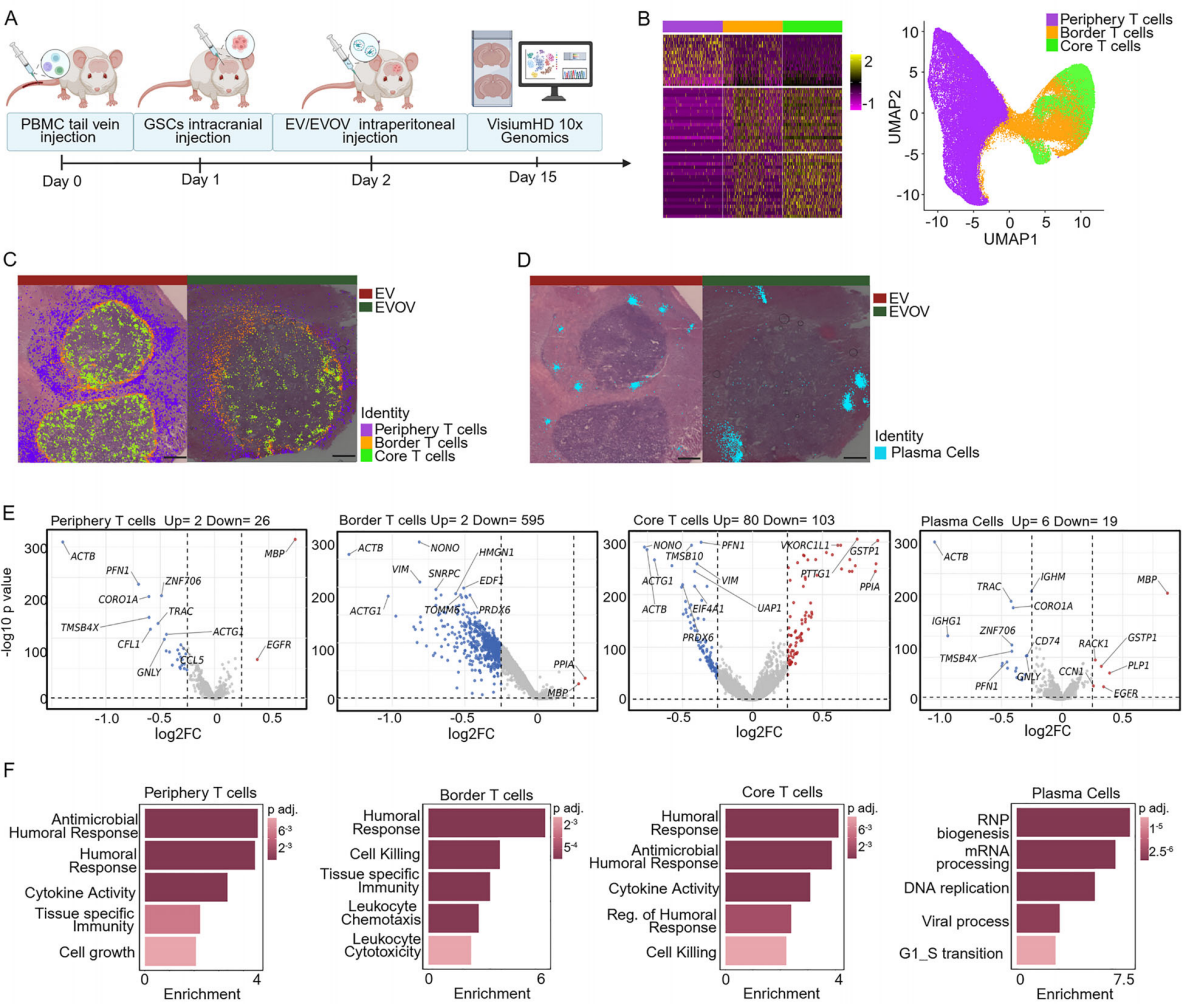
Spatial profiling of systemic immune cells in the NSG model. (A) Schematic overview of the *in vivo* experiment. (B) Heatmap of top 50 differentially expressed marker genes (left) and UMAP projection (right) across annotated clusters in mouse T cells from brain tissue by VisiumHD in EV and EVOV (*n* = 2), Wilcoxon rank‐sum test with Benjamini‐Hochberg correction, FDR≤0.05. (C) Representative images of T cell subpopulation clusters in brain tissue from mice treated with EV or EVOV by VisiumHD, scale bar = 500 µm. (D) Representative images of Plasma cell subpopulation clusters in brain tissue from mice treated with EV or EVOV by VisiumHD, scale bar = 500 µm. (E) Volcano plots of differentially expressed genes in cell subpopulations in brain tissue from mice treated with EV or EVOV (*n* = 2), Presto Wilcoxon with Benjamini‐Hochberg correction, padj<0.05, logFC > 0.25. (F) Bar plots of significantly enriched upregulated pathways (fgsea, padj≤0.1) among genes ranked by differential expression statistics (Presto Wilcoxon/AUC ranking) in annotated cell subpopulations, brain tissue from mice by VisiumHD in EV and EVOV (*n* = 2).

The analysis of canonical markers showed that CD8^+^ T cells predominated, whereas CD4^+^ T cells were detected at low levels in the brain (Figure S10B), although a small yet distinct population of plasma cells was clearly distinguishable (Figures 5D and S10C). Consistent with the peripheral blood analysis, no CD3^−^ lymphocyte population was detected within the tumour, likely reflecting the limited survival of CD3^−^ cells in this mouse model.

In immune cell niches, peripheral T cells exhibited increased effector and cytokine programs, with reduced differentiation and regulatory signals; border and core T cells similarly shifted toward cytotoxic responses, with suppressed cell‐cycle checkpoints and mitotic signatures. Across plasma cell‐assigned regions, EV and EVOV diverged in transcriptomic state, with EVOV showing enrichment for RNA metabolism and proliferation‐associated programs. In contrast, EV showed greater enrichment of immune effector‐related gene sets (Figure 5E,F).

Cytotoxicity scoring showed a trend of decreased effector activity in peripheral niches and modestly increased, more uniform cytotoxic programs at the tumour border (Figure S10D). Proliferation scoring showed limited proliferative activity in immune‐cell regions, with more pronounced signal in selected EVOV‐associated areas (Figure S10E). To provide quantitative support for T‐cell spatial organization, annotated T‐cell bins were summarized as within‐sample proportions across periphery, border, and core regions within each VisiumHD section (Figure S10F). Cytotoxicity‐score distributions further supported niche‐dependent differences between EV and EVOV samples (Figure S10G), most likely as a spatial transcriptomic proxy for T‐cell cytotoxic potential, rather than a direct measure of functional killing. Additional analysis showed that EVOV was associated with lower exhaustion‐marker program scores across T‐cell niches (Figure S10H). EVOV–EV contrasts in selected bin proportions indicated a relative reduction in periphery‐assigned T‐cell bins and a relative enrichment in border‐ and core‐assigned T‐cell bins in EVOV‐treated samples (Figure S10I). The analysis of downregulated pathways further confirmed suppression of cell‐cycle and metabolic programs in these immune compartments (Figure S10J).

Spatially, EV‐treated brains exhibited numerous small, discrete plasma cell clusters, whereas EVOV‐treated brains showed larger, contiguous plasma cell‐assigned territories (Figures 5D and S10C).

The PBMC‐engrafted intracranial model thus allows exploratory association of immune responses in a tumour context, as immune cell infiltration into the brain was observed. This enabled potential detection and spatially resolved profiling of human immune infiltrates within the tumour‐bearing murine brain.

### 3.6 EVOV Reprograms the Tumour Immune Microenvironment in PBMC‐Engrafted NSG Mice with Intracranial GSCs Xenograft

GSC‐derived tumours showed rapid progression, as quantified by BLI, confirming tumour growth in both the EV and EVOV groups (Figure 6A–C). Exploratory analysis of spatial transcriptomic profiling identified distinct transcriptional states within the tumour compartments (Figure 6D). Based on GBM marker‐guided classification (see Materials and Methods), tumour cells of GSC origin were annotated into five subpopulations: MES1 border, MES1 border‐ANG, MES1 border‐IFN, MES2 core, and MES2 core‐hypoxic (Figures 6E and S11A).

**FIGURE 6:**
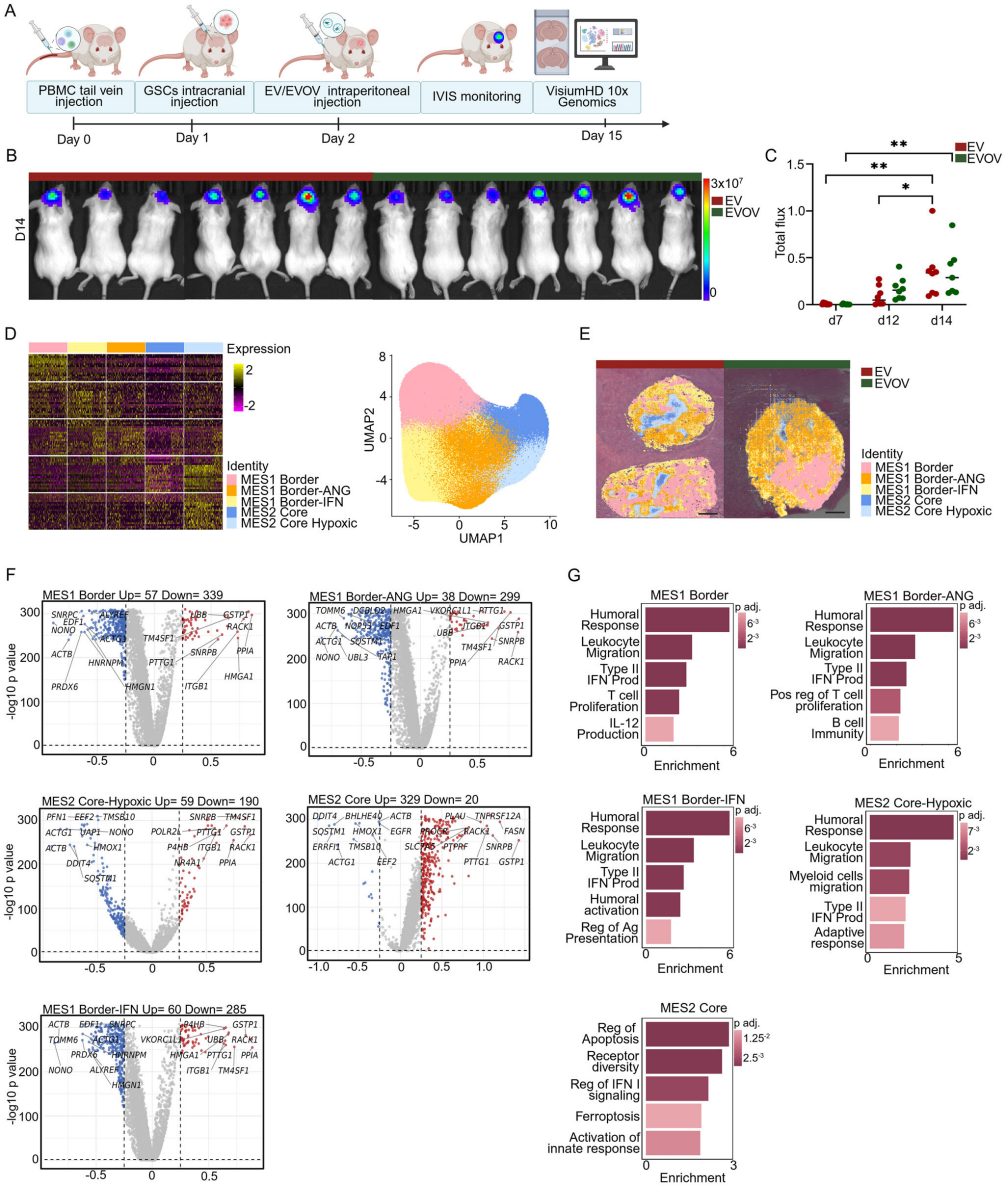
Functional‐spatial analysis of intracranial GSCs NSG model. (A) Schematic overview of the *in vivo* experiment. (B) Representative images of BLI in mice treated with EV or EVOV (*n* = 7) by IVIS. (C) Scatter plots of total flux from BLI in mice treated with EV (*n* = 8) or EVOV (*n* = 7), two‐way ANOVA, *p ≤ 0.05; **p ≤ 0.01. (D) Heatmap of the top 50 differentially expressed marker genes (left) and UMAP projection (right) across annotated clusters in the mouse tumour from brain tissue by VisiumHD in EV and EVOV (*n* = 2), Wilcoxon rank‐sum test with Benjamini‐Hochberg correction, FDR≤0.05. (E) Representative images of tumour cell subpopulation clusters in brain tissue from mice treated with EV or EVOV by VisiumHD, scale bar = 500 µm. (F) Volcano plots of differentially expressed genes in cell subpopulation in brain tissue from mice treated with EV or EVOV (*n* = 2), Presto Wilcoxon with Benjamini‐Hochberg correction, padj<0.05, logFC > 0.25. (G) Bar plots of significantly enriched upregulated pathways (fgsea, padj≤0.1) among genes ranked by differential expression statistics (Presto Wilcoxon/AUC ranking) in annotated cell subpopulations in brain slides from mice by VisiumHD in EV and EVOV (*n* = 2).

Tumour cell clusters showed a trend in functional rewiring: EVOV was associated with upregulation of IFN‐related and leukocyte migration programs in MES1 and MES2 subpopulations, while attenuating DNA replication, mRNA processing, and translation‐associated modules (Figures 6F,G and S11B). Interestingly, among the most upregulated genes was peptidylprolyl isomerase A (PPIA), a marker of innate immune activation (Shankaran et al. 2001, Wu et al. 2023, Benci et al. 2016), which showed elevated expression in border and core T cell niches (Figure 5E) and across multiple tumour clusters (Figure 6F), suggesting active sensing of viral antigens within the TME. To provide quantitative support for tumour‐state spatial organization, annotated tumour‐cell bins were summarized as within‐sample proportions across biological replicates. Importantly, enrichment of IFN‐associated programs was not accompanied by expansion of the MES1 Border‐IFN compartment, whose lower relative representation in EVOV‐treated tumours may reflect either selective loss or redistribution across transcriptional states (Figure S11C). Additionally, spatial mapping of leukocyte migration and IFN production revealed that these niches did not fully overlap, while leukocyte migration remained reduced in hypoxic tumour regions in both EV and EVOV conditions (Figure S11D).

Spatial visualization further demonstrated the organization of these tumour states relative to T‐cell populations localized within peripheral, border, and core tumour niches, indicating immune‐cell penetration beyond the tumour margin (Figure S12A). UMAP projections showed the expression of *CD8A, CD4, FOXP3, MKI67, CASP3*, and *GZMB* within the T‐cell compartment, whereas *MKI67, CASP3*, and *GZMB* displayed heterogeneous expression across tumour cells (Figure S12B).

Orthogonal immunofluorescence analysis confirmed the presence of immune cells within tumour regions, including proliferating and GrB‐expressing immune effector cells. Quantification across the analysed regions demonstrated that EVOV treatment increased immune cell proliferation, concomitant with enhanced tumour cell apoptosis (Figure S13A).

Descriptive effect‐size and replicate‐concordance analyses supporting the integrity of the VisiumHD dataset are shown in Figure S14A,B.

Together, these findings suggest that EV and EVOV delivery in a PBMC‐engrafted mouse model impacts both the immune and tumour compartments in the brain, particularly within humoral response pathways. Upon single‐dose intraperitoneal administration of vesicles, immune cells recruited into the TME adopted a checkpoint‐low, IFN‐licensed phenotype, with EVOV associated with reduced T‐cell exhaustion and spatially distinct immune and tumour‐cell transcriptional states. Both EV and EVOV dosing were well tolerated in this model, with no overt toxicity or evidence of ectopic tumour dissemination, supporting their feasibility as non‐injurious immunomodulatory inputs in PBMC‐engrafted NSG mice.

### 3.7 EVOVs Exhibit an Altered MHC‐I Putative Immunopeptidome

Comprehensive immunopeptidomic profiling of EVs and EVOVs derived from GSCs was performed using DIA‐MS to investigate whether EVOVs harbour a distinct, immunologically relevant repertoire of MHC‐I‐associated peptides (Figure 7A). A total of 41,937 peptides were identified across all samples. Chromatographic profiles confirmed consistency of DIA‐MS runs and robust separation between EV and EVOV samples (Figure S15A–C). The analysis shows ∼1900 predicted MHC‐I‐associated peptides, based on integration with the HLA Ligand Atlas, Immune Epitope Database (IEDB), MHC Motif Atlas, and peptides detected directly from serial tumour biopsies of GBM patients treated intratumourally with oHSV (CAN‐3110) (Ling et al. 2025) (Figures 7B and S15B), highlighting a shared antigenic space between EV/EVOV cargo and the clinically observed immunopeptidome.

**FIGURE 7:**
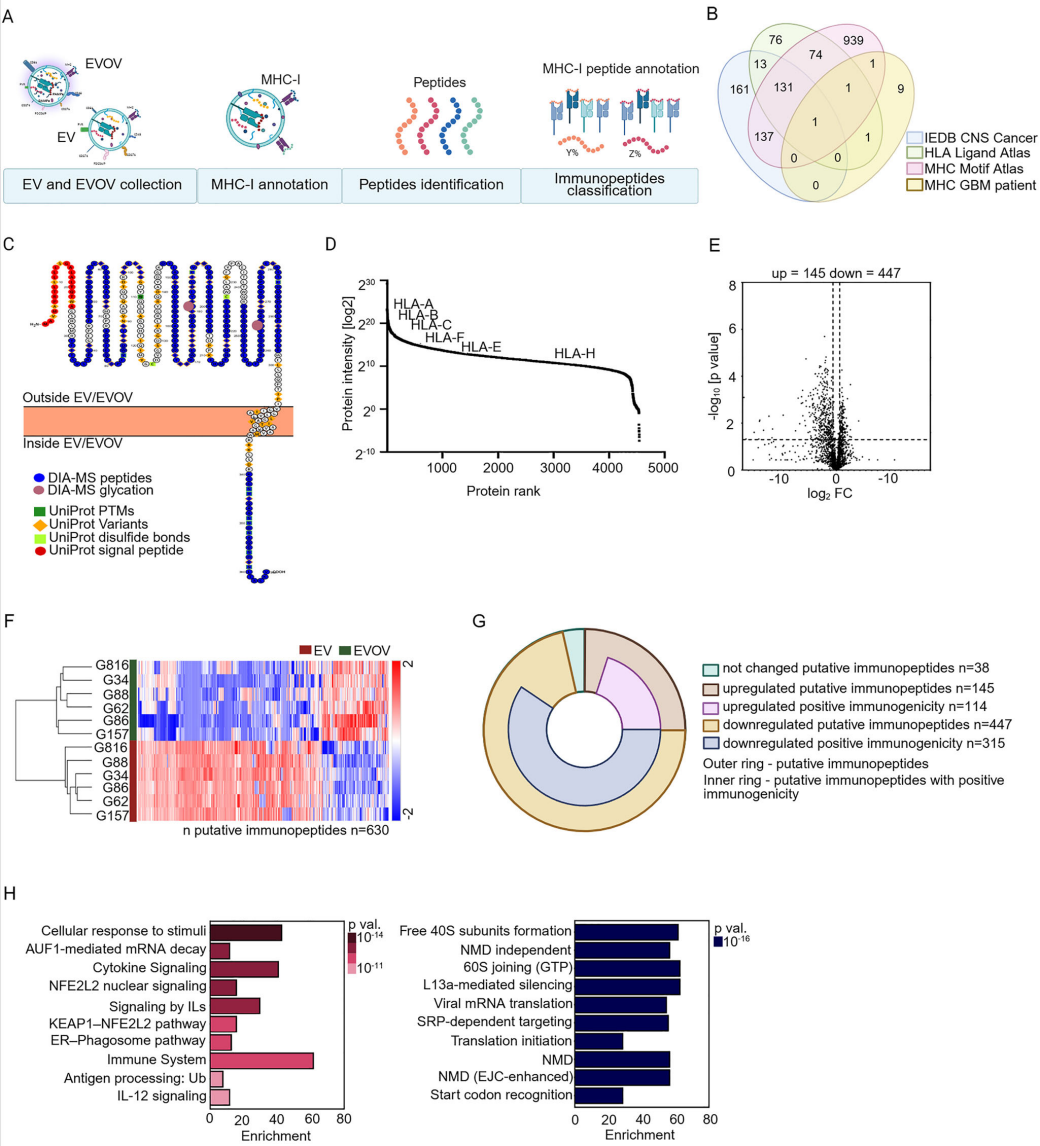
Immunopeptidomic landscape of EVs and EVOVs. (A) Schematic overview of human peptide analysis. (B) Venn diagram of peptide overlaps between immunopeptide databases and MHC‐detected peptides from GBM patients treated with oHSV‐1. (C) Schematic representation of HLA‐A domain map with DIA‐MS detected peptide regions and glycation and UniProt proteoform. (D) Rank plot of MHC‐I protein relative abundance by DIA‐MS among detected proteins in EV and EVOV (*n* = 6). (E) Volcano plot of EV and EVOV (*n* = 6) immunopeptide abundance by DIA‐MS, paired *t*‐test *p* ≤ 0.05. (F) Heatmap and hierarchical clustering of significantly deregulated immunopeptides (*p ≤ 0.05, FC > 1.5) by DIA‐MS in EV and EVOV (*n* = 6). (G) Donut chart summarizing deregulated immunopeptides with positive immunogenicity by DIA‐MS in EVOV vs. EV (*n* = 6). (H) Enrichment chart of source genes from upregulated (left) and downregulated (right) immunopeptides by DIA‐MS in EVOV vs. EV (*n* = 6).

The global protein intensity ranking showed robust detection of MHC‐I proteins, with HLA‐A, ‐B, and ‐C among the higher‐abundance species and HLA‐E, ‐F, and ‐H among the lower‐abundance species (Figure 7C,D). Relative protein abundance analysis confirmed comparable levels of each HLA isoform between EV and EVOV (Figure S15D). MS‐based peptide mapping revealed high coverage of the HLA‐A protein sequence, supporting its active participation in antigen presentation (Figure 7C). Despite similar levels of MHC‐I proteins, the composition of the immunopeptidome was substantially altered in EVOVs compared to EVs. Analysis identified 630 significantly deregulated immunopeptides, including 145 upregulated and 447 downregulated in EVOVs (Figures 7E,F and S15E,F). Clustering of significantly deregulated immunopeptides showed a skew toward downregulation in EVOV compared with EV (Figure 7E,F). This global reduction in peptide diversity, despite comparable vesicle output, results in a less heterogeneous yet more functionally specialized EV cargo.

Notably, 114 of the 145 upregulated peptides and 315 of the 447 downregulated peptides were predicted to exhibit positive immunogenicity (Figure 7G), indicating a reconfiguration of the antigenic repertoire in EVOVs. Source gene enrichment further supported a shift toward antigen processing and cytokine signalling (upregulated) (Figure 7H left) from pathways such as viral mRNA translation and nonsense‐mediated decay (NMD) signalling (downregulated) (Figure 7H right).

Importantly, the identified immunopeptides also carried a range of physiologically relevant PTMs, including acetylation (Acetyl), carbamidomethylation (CAM), phosphorylation (Phospho), oxidation (OX), glycation (notably in HLA‐A peptides), and hexosylation (Hex), which was not restricted to the C‐terminal residues (Figures 7C and S15G). The detection of these PTMs, in combination with high‐resolution DIA‐MS, supports the notion that a subset of the detected peptides is not merely tryptic fragments, but includes biologically derived and functionally relevant immunopeptides.

Capitalizing on our previous findings showing that oHSV exposure remodels the GBM secretome (Godlewski et al. 2021), we analysed an independent EV/EVOV cohort in the present study to confirm broader remodelling of protein cargo and to relate it to the immunopeptidomic changes observed here. Global proteomic profiling identified over 5200 proteins in heterogeneous GSCs, clearly separating EV and EVOV proteomes (Figure S16A). Reproducibility of protein quantification was further validated by coefficient of variation analysis and peptide intensity distributions, confirming robust measurement across biological replicates (Figure S16B–D). Differential abundance analysis revealed 1914 proteins to be significantly deregulated (Figure S16E). Protein‐level scoring showed reduced tumour purity and enhanced immune cell‐associated signatures in EVOVs (Figure S16F). Notably, functional proteomic mapping revealed that EVOVs are enriched in immunological and protein‐folding categories and depleted in translation‐associated components (Figure S16G). Deconvolution analysis of proteomic profiles predicted enhanced infiltration of activated immune cells, including NK cells, NKT cells, activated CD8^+^ T cells, macrophages, and Type 1 CD4^+^ T cells, in EVOVs compared to EVs (Figure S16H).

Together, these findings indicate that although quantitatively MHC‐I protein expression remains stable, EVOVs exhibit a remodelled, more immunogenic MHC‐I‐associated peptide landscape, enriched in PTM‐bearing peptides that suggest facilitation of antitumour immune activation. The added global proteomic analysis further supports the conclusion that EVOV‐mediated immunomodulation is not limited to putative immunopeptides but also involves broader remodelling of immune‐associated protein cargo.

### 3.8 EVOV‐Mediated Antigenic Remodelling Drives MHC‐I–Dependent Immune Activation

An experimental platform (Figure 8A) of patient‐derived GSC *ex vivo* spheroid cultures was characterized by the expression of stemness and MES markers (Figure S17A–B). Quantitative PCR analysis showed the presence of oHSV‐1 viral transcripts in infected GSCs (Figure 8B). However, in the EVOV fraction, only the early entry of oHSV‐1 transcript ICP0 and a low level of the virus's genomic DNA were detected, compared to EVs (Figure 8C) (Chudzik et al. 2010).

**FIGURE 8:**
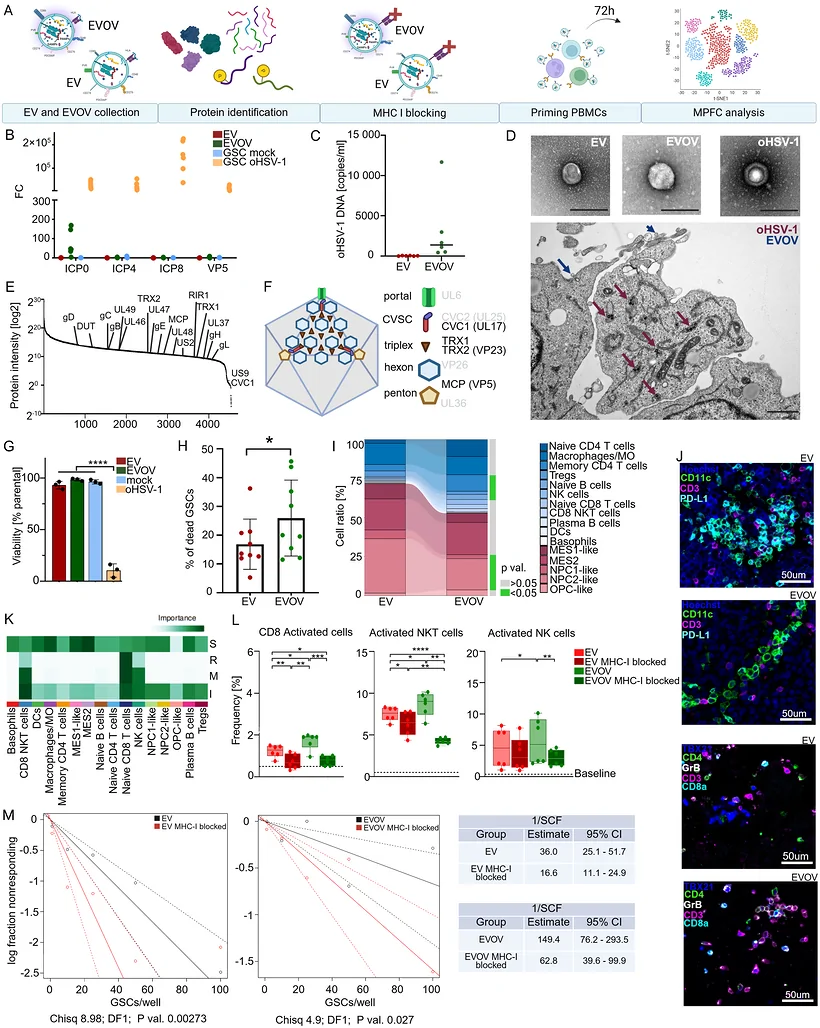
MHC‐I Blockade in EVOVs Diminishes PBMC‐Mediated Antitumour Responses against GSCs. (A) Schematic overview of EV and EVOV characterization and MHC‐I blocking analysis. (B) Scatter plot of qPCR quantification of oHSV‐1 transcripts in mock‐ or oHSV‐1‐infected GSCs and EV or EVOV (*n* = 6). (C) Scatter plot of qPCR quantification of oHSV‐1 genomic DNA (copies/ml) in EV and EVOV (*n* = 6). (D) Representative TEM micrographs of purified EVs, EVOVs, oHSV‐1 particles (top, scale bar = 200 nm), and oHSV‐1‐infected GSCs (bottom, 1 µm). (E) Rank plot of oHSV‐1 protein relative abundance by DIA‐MS among detected proteins in EV and EVOV (*n* = 6). (F) Schematic representation of viral capsid proteins detected (black) and not detected (grey) by DIA‐MS. (G) Bar plot of GSC viability by MPFC after 72 h of treatment with EV, EVOV, mock, or oHSV‐1 (*n* = 3); paired *t*‐test, *p ≤ 0.05. (H) Bar plot of GSC viability derived from organoids (GSCs *n* = 3; PBMCs *n* = 3) by MPFC; paired *t*‐test, *p ≤ 0.05. (I) Sankey plot of cell subpopulation clusters in organoids by scRNA‐seq in EV and EVOV (*n* = 3), paired *t*‐test, one‐tailed, *p ≤ 0.05. (J) Representative immunofluorescence images of organoids by MPI; scale bar = 50 µm. (K) Heatmap of the MHC‐I signalling pathway network from organoids by scRNA‐seq in EVOV (*n* = 3). S‐sender, R‐receiver, M‐mediator, I‐influencer. (L) Bar plots of CD3^+^ and CD3^−^ cell clusters of PBMC (*n* = 2) primed with EV, EVOV, EV MHC‐I blocked, or EVOV MHC‐I blocked (*n* = 3) by MPFC, paired *t*‐test, *p ≤ 0.05; **p ≤ 0.01; ***p ≤ 0.001; ****p ≤ 0.0001. (M) ELDA plots for GSCs (G157, G816) from co‐cultures with PBMC primed with EV, or EV MHC‐I blocked (left), or EVOV, or EVOV MHC‐I blocked (right), ELDA likelihood ratio test (χ^2^). Stem cell frequencies 1/x with 95% confidence intervals.

TEN analysis of cells after mock or oHSV‐1 infection confirmed a similar vesicular morphology of EVs and EVOVs, distinguishing these vesicles from hedral oHSV‐1 particles (Figure 8D). DIA‐MS further characterized the viral cargo in EVOVs, identifying 19 oHSV‐1‐derived proteins in EVOVs but not in EVs (Figure 8E). Most of the detected viral proteins were associated with the viral capsid or tegument (Figure 8F). At the same time, key early lytic transcripts were absent (Figure 8B), reinforcing the notion that these particles are replication‐deficient.

Subsequently, treating non‐infected/parental GSCs with EVOVs did not impair cell viability, unlike active viral infection (Figure 8G), demonstrating EVOVs’ lack of infectivity.

GSC death in organoids formed with EVOV‐primed PBMCs was significantly increased and associated with a decreased proportion of GSCs (Figure 8H,I). To determine whether EVOV priming recapitulates the immunostimulatory effect of direct viral exposure and enhances killing of virus‐conditioned tumour cells, PBMCs were primed with PBS, EV, EVOV, or oHSV‐1 and subsequently analysed for *IFNG* expression. EVOV induced a marked increase in *IFNG* transcript levels, comparable to that observed after direct oHSV‐1 stimulation, whereas EV elicited only a modest response (Figure S17C). In PBMC–GSC organoids, pretreatment of GSCs with oHSV‐1 resulted in a pronounced reduction in GSC viability, while PBMC viability decreased by only slightly, indicating preferential cytotoxicity toward tumour cells (Figure S17D). Importantly, within organoids containing oHSV‐1‐pretreated GSCs, PBMC priming with EVOV further reduced GSC viability compared with EV priming, whereas PBMC viability was not comparably affected (Figure S17E). Consistent with these findings, the combination of oHSV‐1 pretreatment of GSCs and EVOV priming of PBMCs produced the strongest cytotoxic effect on GSCs and the most pronounced disruption of organoid structure, although a smaller reduction in PBMC viability was also observed (Figure S17F). Together, these findings indicate that EVOV‐primed PBMCs act additively with oHSV‐1 pretreatment to enhance GSC cytotoxicity, with a modest reduction in PBMC viability.

Importantly, PBMCs primed with EVOV did not uniformly target all GSC subpopulations (e.g., MES2 showed relative preservation), whereas the most pronounced depletion was observed in NPC‐like cells. In parallel, EVOV exposure reduced Treg abundance and expanded effector immune subsets, including memory CD4^+^ T cells and naïve B cells, within the organoids (Figures 8I and S17G).

Immunophenotyping of organoids using MPI further supported these findings, revealing that EVOV priming reduced PD‐L1^+^CD11c^+^ immunosuppressive myeloid cells and was accompanied by a pronounced increase in fully activated CD8^+^GrB^+^TBX21^+^ cytotoxic T cells, indicating a shift toward a more pro‐inflammatory and permissive immune microenvironment (Figures 8J and S18A). Consistent with this phenotype, scRNA‐seq analysis showed that GSCs within EVOV‐primed PBMC organoids upregulated classical MHC‐I molecules (*HLA‐A/B/C*) and key components of antigen processing and peptide loading (*TAP1, B2M, SEC24D, PIK3C3*), suggesting enhanced antigen‐presenting capacity (Figure S8D,E).

Analysis of the MHC‐I signalling network revealed that EVOV‐primed PBMCs in organoids displayed a distinct, more immune‐engaged communication pattern compared with the EV group. In the EVOV organoid group, multiple GSC states, particularly the MES and NPC subclasses, acted as strong MHC‐I senders. At the same time, cytotoxic lymphocytes, including CD8 NKT and NK cells, showed markedly higher receiver activity, indicating enhanced responsiveness to antigen presentation. In contrast, the EV group of organoids exhibited a narrower, MES‐dominated network with weaker cytotoxic lymphocyte engagement, reflecting a less coordinated and less immunogenic MHC‐I communication profile (Figures 8K and S17H).

To investigate whether EVOV‐driven activity of PBMCs relies on MHC‐I peptide‐binding signalling, which leads to anti‐cancer cytotoxicity, MHC‐I was antibody‐blocked on EVs and EVOV before priming PBMCs, significantly reducing IFN‐γ secretion and bringing levels to near‐baseline (Figure S17I–J). This was accompanied by a marked decrease in the activation of CD8^+^ T cells, CD8^+^ NKT cells, and NK cells, particularly in PBMCs primed with EVOVs (Figures 8L and S17K). These findings suggest that the enhanced effector function induced by EVOVs heavily depends on the presentation of EVOV‐derived peptides *via* MHC‐I.

EVOV‐primed PBMCs were evaluated for cytotoxicity against GSCs, with MHC‐I blocked using vesicles. ELDA demonstrated that EVOV consistently and significantly reduced GSCs' stemness, more strongly than EV. MHC‐I blockade restores sphere‐forming capacity, demonstrating MHC‐I‐dependent anti‐cancer activity in PBMCs (Figures 8M and S17L–M).

To further dissect the antigenic component of EVOV cargo, MHC‐I immunoprecipitation was performed on EV and EVOV samples. A representative MHC‐I IP Western blot confirmed successful enrichment of MHC‐I complexes (Figure S18B). Peptide‐level profiling revealed EVOV‐associated remodelling of the MHC‐I IP peptide repertoire, including EVOV‐upregulated, EVOV‐downregulated, and stable peptide groups (Figure S18C). Functional annotation of the corresponding source genes showed that EVOV‐upregulated MHC‐I IP peptides were linked to neutrophil/granulocyte activation, leukocyte degranulation, and immune‐response‐related programs. In contrast, EVOV‐downregulated peptides included mitochondrial respiration and ATP biosynthesis pathways together with partially overlapping neutrophil/leukocyte immune terms. Stable peptides were mainly associated with oxidative stress, apoptosis, and cellular stress‐response programs (Figure S18D). Integration of source genes corresponding to MHC‐I IP–detected peptides with scRNA‐seq data from GSC–PBMC organoids, together with module‐score analysis, further showed EVOV‐associated induction of antigen‐processing, IFN‐response, and cytotoxicity‐associated transcriptional programs in immune and tumour compartments (Figure S18E,F). Finally, overlap analysis between MHC‐I IP peptide source genes and the global EV/EVOV DIA‐MS proteome showed that most MHC‐I IP source genes were independently detected in the broader EV/EVOV proteomic cargo (Figure S18G). The remaining IP‐only source genes likely reflect the selective enrichment of low‐abundance MHC‐I‐binding peptide species by immunoprecipitation, thereby revealing antigenic peptides that are not detectable at the source‐protein level in the total proteome. Together, the differences between EV and EVOV MHC‐I IP‐derived peptide repertoires support the conclusion that oHSV‐1 infection reshapes the MHC‐I peptide repertoire in GSC‐derived vesicle preparations.

Overall, these data demonstrate that EVOVs activate a potent, MHC‐I‐restricted cytotoxic program that enhances immune infiltration, promotes GSC killing, and functionally impairs cancer cell stemness by reprogramming antigenic signals presented on MHC‐I through oHSV‐1.

These findings establish EVOVs as distinct, non‐replicative entities that carry viral and immune‐modulatory content yet remain replication‐deficient, with the potential to reshape the TME.

### 3.9 EVOV‐derived Immune and Antigen‐presentation Programs Align with Improved Survival in CAN‐3110 Post‐treatment

Pathways identified as significantly upregulated in EVOV‐primed GSC‐PBMC organoids by scRNA‐seq (Figure 3) were converted into ssGSEA signature scores and evaluated for associations with survival across three independent GBM bulk RNA‐seq cohorts (Figures 9, S19 and S20).

**FIGURE 9:**
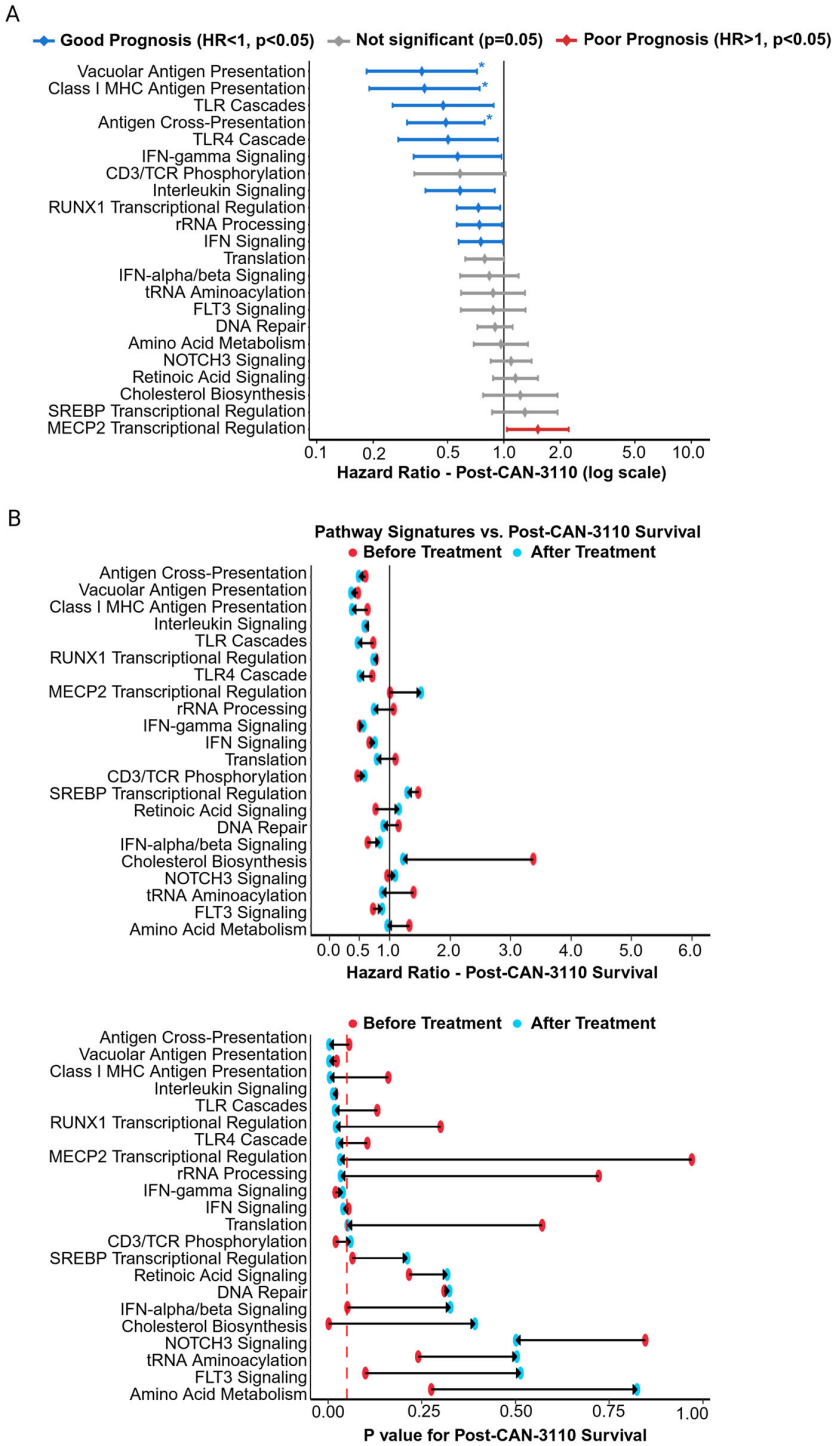
Post‐treatment Survival Associations of EVOV‐upregulated Pathway Signatures in the CAN‐3110 Cohort. (A) Forest plot of Cox proportional hazards models for the association between post‐treatment pathway ssGSEA signature scores and post‐CAN‐3110 survival in IDH‐WT Arm A/B patients, HR shown on a log10 scale, 95% CI, Benjamini–Hochberg, *FDR≤0.05. (B) Forest plots comparing pre‐treatment (red) and post‐treatment (blue) pathway ssGSEA signature associations with post‐CAN‐3110 survival in IDH‐WT Arm A/B patients; top: HR (log10 scale) with reference line HR = 1, bottom: *p ≤ 0.05; Cox proportional hazards models.

In the TCGA cohort, higher immune activation scores are paradoxically associated with poorer overall survival (Figure S19A). In contrast, in the CGGA cohort, the examined signatures are not significantly associated with survival (Figure S19B). To determine whether this cohort‐dependent behaviour was influenced by tumour subtype composition, the TCGA cohort was further stratified according to transcriptomic subtype (Figure S19C–F). Among the evaluated subtypes, associations between immune‐inflammatory pathway activity and adverse outcome were most evident in the MES cohort. In this subtype, increased immune‐inflammatory pathway activity was associated with poorer outcome, with interleukin signalling remaining significant after FDR correction in the multivariate model (Figure S19F). In contrast, subtype‐restricted analyses in the other groups showed weaker or non‐significant associations, consistent with reduced statistical power after stratification.

In CAN‑3110–treated patients, higher pre‑treatment immune signature scores (including CD3/TCR phosphorylation, vacuolar antigen presentation, IFN‐γ signalling, and interleukin signalling) are associated with improved post‑treatment survival, whereas elevated cholesterol biosynthesis pathway activity is a significant predictor of worse outcome (Fig S20A). In the same cohort, higher post‑treatment immune signature scores across all three antigen presentation pathways (vacuolar antigen presentation, class I MHC antigen presentation, and antigen cross‐presentation) are significantly associated with improved survival, with antigen presentation remaining significant after FDR correction (Figure 9A). Direct comparison of pre‐ versus post‐treatment signature associations shows changes in hazard ratios and p‑values in the CAN‑3110 dataset, considering all IDH‑WT Arm A/B patients (Figure S19) and the subset of HSV1‑seropositive patients (Figure S20B). In both analyses, higher post‑treatment immune scores remain associated with better survival (Figures 9B and S20B).

Overall, EVOV‐induced immune and antigen‐presentation programs show cohort‐dependent baseline prognostic behaviour but converge in the CAN‐3110 setting, where higher post‐treatment antigen‐presentation/immune‐signature scores are robustly associated with improved survival, supporting these pathways as therapy‐conditioned, clinically relevant correlates of benefit.

## 4 Discussion

GBM is known for its high recurrence rate and resistance (Stupp et al. 2005, Lim et al. 2018) to both cytotoxic and immunotherapies, highlighting the need for approaches that prime peripheral antitumour immunity and support immune recruitment to the tumour site, including vaccine‐like strategies (Kiel et al. 2025). OVs offer a promising strategy, combining direct tumour cell killing with the delivery of immunostimulatory cues and the induction of antiviral and antitumour immunity. As clinical benefits have already been observed in a subset of patients across OV platforms, the field is increasingly focused on improving targeting, arming, dosing, and combination regimens (Ling et al. 2023, Todo et al. 2022, Lowenstein et al. 2025). By that rationale, EVOVs released by GSCs during early oHSV‐1 infection are poised to be a modular extension of OV mode of action, capable of packaging antigenic and adjuvant‐like signals into a vesicular format that can prime immune responses while curbing liabilities associated with viral replication. Conceptually, this would complement existing OV strategies in GBM, serving as an adjuvant.

In a PBMC‐engrafted intracranial GSC NSG model, systemic EVOV administration was associated with increased activation of peripheral CD4^+^ helper T cells and a reduced frequency of PD‐1^+^ CD8^+^ T cells in blood, indicating measurable systemic immune modulation within the experimental window. Spatial transcriptomic profiling further supported immune engagement at the tumour site, including enrichment of effector immune populations and an IFN‐linked, MHC‐I‐associated transcriptional program that paralleled key *ex vivo* organoid observations, supporting convergent EVOV‐associated immune activation across model systems.

In clinical oHSV‐1 studies in GBM, post‐treatment increases in intratumoural T cell infiltration and immune activation signatures have been linked to improved outcomes in subsets of patients, supporting the relevance of therapy‐associated immune remodelling (Ling et al. 2023). Within the exploratory *in vivo* model, spatial mapping revealed a compartmentalized architecture of immune cells consistent with an organized local response. At the tumour border, a key interface of immune‐tumour interaction, T cell‐enriched regions displayed elevated cytotoxic programs and reduced expression of checkpoint/exhaustion‐associated transcripts relative to other tumour regions (Greenwald et al. 2024). At the same time, an IFN/MHC‐driven state, accompanied by lower expression of multiple exhaustion markers (*PDCD1, LAG3, HAVCR2, TIGIT*) in T cell clusters (Wherry and Kurachi 2015, Blank et al. 2019), was observed within the tumour core, especially in the EVOV group. Plasma cell‐enriched territories with proliferative signatures were also detected, suggesting the emergence of localized immune niches; however, confirmation of tertiary lymphoid‐like organization would require additional marker‐based spatial validation (Schumacher and Thommen 2022, Helmink et al. 2020). Notably, these spatial features were consistent with scRNA‐seq profiles showing increased representation of activated memory CD4^+^ T cells, and with IFN‐responsive transcriptional signatures (Cloughesy et al. 2019) observed in GSCs. The upstream drivers cannot be resolved from transcriptomic snapshots alone, but the observed programs are compatible with sustained helper‐associated activation in an inflammatory milieu.

The MES2 core remained a notable exception, showing a program dominated by stress and innate‐response features, with only a limited/partial type‐I IFN‐associated signature. This pattern is consistent with an early‐response, potentially antigen‐independent immune state, and thus with a niche of increased resistance to efficient immune control (Neftel et al. 2019, Ricklefs et al. 2018, Puchalski et al. 2018, Mineo et al. 2016).

By shifting from macroscopic observations to underlying molecular mechanisms, critical signals that initiate the immune response were revealed. One of the most consistently upregulated genes in the EVOV group across both T cells and tumour cells was *PPIA* (encoding Cyclophilin A), a multifunctional host factor canonically co‐opted by replicating viruses, such as HIV‐1 (Zhou et al. 2012, Franke et al. 1994). The induction of *PPIA*, despite the absence of productive viral replication, is consistent with early host sensing of vesicle‐associated viral cues and/or cellular stress pathways (Yurchenko et al. 2010, Liu et al. 2017). Mechanistically, this upregulation would be linked to IFN‐driven transcriptional programs, including activation of STAT1 and IRF1, as well as expression of downstream IFN‐stimulated genes such as *ICAM1* and *TAP1* (Shankaran et al. 2001, Wu et al. 2023, Benci et al. 2016), which were co‐upregulated with *PPIA*. This suggests that *PPIA* is a relevant marker of non‐cytopathic immune sensing, but its precise functional contribution to the antiviral and antitumour programs remains to be determined.

In parallel to *in vivo* analyses, EVOV priming of PBMCs in organoids also triggered antiviral signalling. Two complementary classes of signals, as antigenic cargo and cytokine‐like mediators (e.g., IP‐10 (Mao et al. 2023)), were associated with IFN‐γ signalling across all major GSC subpopulations. *Ex vivo* antiviral mimicry coincided with broad activation of IFN‐responsive transcription factors. These findings, which align with prior evidence that *STAT1* and *IRF1* coordinate MHC‐I expression and sensitize tumours to T cell‐mediated cytotoxicity (Shankaran et al. 2001, Wu et al. 2023), support the use of EVOVs as an adjuvant to induce an immune response in GBM.

The functional outcome of IFN signalling is tightly linked to the expression dynamics of immunological checkpoints. EVOV priming seemingly counterbalances this restraint in 3D organoid settings, where immune activation was associated with MHC‐I upregulation and tumour cell death driven by infiltration of GrB^+^ CD8^+^ T cells and NK cells, alongside APC expansion. Notably, a concurrent reduction in PD‐1^+^ immunosuppressive populations indicated a shift toward an effector‐dominant immune landscape that is not captured by conventional 2D systems (Magré et al. 2023). These discoveries support the EVOV priming mechanism as a potential functional stratification tool for identifying patient cohorts most likely to benefit from EVOV‐based therapies, thereby reducing reliance on extensive in vivo testing prior to clinical application.

The non‐uniform transcriptional response across GSC states highlights differential sensitivity to EVOV (Neftel et al. 2019, Richards et al. 2021, Hara et al. 2021, Chanoch‐Myers et al. 2022). The preferential induction of innate immune and cell‐death‐associated programs in MES2 states, contrasted with the rapid activation of antigen‐processing and MHC‐I‐related pathways in NPC‐like cells, suggests divergent yet potentially complementary trajectories of EVOV‐associated tumour remodelling (Neftel et al. 2019, Richards et al. 2021). Importantly, the data further support enhanced coupling between antigen‐presenting programs and effector lymphocyte activity, consistent with increased coordination between the cytotoxic and helper compartments. The functional relevance of this axis is supported by the reduced sphere‐forming capacity (Hu and Smyth 2009) upon EVOV exposure. Notably, MHC‐I blockade restored sphere formation, supporting an MHC‐I‐dependent immune‐recognition component of the EVOV effect and arguing against a purely tumour‐intrinsic, direct cytotoxic mechanism (Shankaran et al. 2001, Wu et al. 2023).

Despite originating from oHSV‐1‐infected GSCs, characterization confirmed that EVOVs are non‐infectious, replication‐deficient vesicular entities rather than intact virions (Deschamps and Kalamvoki 2018, Kalamvoki et al. 2014), as determined by strict timing (early phase of infection to avoid secondary spread) and low dose (to minimize carryover of free virus). EVOVs contained traces of oHSV‐1‐associated molecular cargo, yet essential components required for productive virion assembly/replication (e.g., UL46, VP5) were not detectable (Huang and Wagner 1994, Vittone et al. 2005). Functional assays further confirmed that EVOV lacked replication competence and did not infect parental GSCs. Consistent with this non‐replicative profile, PBMC monoculture dose‐escalation analyses did not show overt PBMC toxicity, as PBMC viability remained preserved at a 2.5‐fold higher EVOV dose and MPFC profiling did not reveal a global collapse of CD3^+^ or CD3^−^ immune composition. Crucially, while non‐replicative, EVOV retained potent immune‐stimulatory cargo, including oHSV‐1 DNA and early transcripts (e.g., *ICP0*) (Kalamvoki et al. 2014), consistent with a highly immunogenic payload that triggers the IFN‐γ response described above (Deschamps and Kalamvoki 2018). EVOVs also exhibited reduced diversity of vesicle‐associated proteins and peptides compared with EVs, despite comparable particle numbers and total protein yields, suggesting that oHSV‐1 conditioning reshapes EVOV cargo toward a more restricted and functionally directed immunomodulatory repertoire. The translational relevance of the EVOV proteome cargo as a readout of antiviral immune pressure was supported by intersecting the EVOV peptidome with immunopeptides detected in serial rGBM biopsies during intratumoural oHSV‐1 therapy (CAN‐3110), revealing a shared antigenic repertoire (Ling et al. 2023).

Consistent with this IFN‐driven state and the immunogenic potential of the EVOV cargo, their immunopeptidomic profiling revealed selective enrichment of predicted canonical MHC‐I binding peptides with binding motifs (8‐11 amino acid residues). Additionally, peptides’ PTMs reinforce their biological relevance and immunogenic potential (Mei et al. 2020, Kacen et al. 2023, Castillo‐Peña and Molina‐Pinelo 2023, Liu et al. 2018, Wolfers et al. 2001), as evidenced by functional assays demonstrating CD8^+^ T cell activation following EVOV priming, both *ex vivo* and *in vivo*.

From a translational perspective, EVOVs provide a safety rationale distinct from live oHSV‐1 therapy as they are designed to transfer oHSV‐1‐induced immunogenic cues without productive viral replication. In this study, EVOVs were generated using a low‐dose, early‐conditioning window (MOI 0.1, 12 h), selected to capture early virus‐induced immune remodelling while maintaining a safety margin against productive virion accumulation. This reduces risks related to uncontrolled viral spread, viral shedding, and antiviral clearance, which remain important challenges for live OV approaches (Totsch et al. 2019, Nguyen and Saha 2021).

Nevertheless, EVOVs would require rigorous GMP‐compatible manufacturing and batch qualification (Ng et al. 2022, Takakura et al. 2024). Our proteomic data support a quality‐control framework based on positive markers of oHSV‐1 exposure and host‐cell response, including gD, gC, UL47, and PPIA, together with the absence of markers associated with mature virion or virion‐like material. Additional controls, such as CD63, would further confirm vesicular identity, while other markers not detected in EVs and EVOV, such as POLA1, would exclude contamination from cellular material.

EVOVs are also conceptually distinct from whole‐tumour lysate vaccines. While lysates provide broad tumour antigen content, they represent less‐defined mixtures of intracellular, nuclear, and damaged‐cell components (Kiel et al. 2025). In contrast, EVOVs are cell‐free vesicular products that may combine tumour‐derived antigens with oHSV‐1‐induced immunostimulatory signals in a more compartmentalized and quality‐controllable format (Kiel et al. 2025, Balakrishnan et al. 2025). Thus, EVOVs are well positioned as controllable, non‐replicative biologics whose translational feasibility will depend on standardized production, validated potency assays, infectivity testing, and stringent release criteria.

To further place these EVOV‐induced programs into a clinical context, EVOV‐derived signatures were mapped onto independent GBM bulk RNA‐seq cohorts, including standard‐of‐care GBM populations (TCGA, CGGA) and the CAN‐3110 oHSV‐1 trial dataset (Ling et al. 2025). As expected, the EVOV‐dependent gene signatures enriched for immune activation pathways in the TCGA and CGGA cohorts are linked to poor prognosis, consistent with inflammatory, MES‐like tumours embedded in a hypoxic niche in the absence of effective immunotherapy. In contrast, the same signature is associated with improved survival in patients treated with CAN‐3110 immunotherapy, indicating that EVOV‐driven upregulation of antigen presentation converts otherwise maladaptive inflammation into a therapeutically productive, T‐cell–engaging state.

Given these spatially organized immune activation patterns, a therapeutic window was proposed: intraperitoneal administration of EV or EVOV 2 days after intravenous delivery of PBMCs. This regimen was designed to promote the recruitment of immune effector cells into the TME, as evidenced by spatial transcriptomic readouts annotated using human data (Neftel et al. 2019, Greenwald et al. 2024). High‐resolution profiling methods were integrated to capture the spatial organization and cellular complexity of the GBM immune microenvironment across *ex vivo* and *in vivo* settings. The current tiered workflow, comprising EVOV priming of human PBMCs, GSC‐PBMC organoid assays (Jacob et al. 2020), and PBMC‐engrafted animals with intracranial xenografts (Morillon et al. 2020, Chuprin et al. 2023), is designed to bridge the gap between existing GBM models (Magré et al. 2023, Chuprin et al. 2023, Yuki et al. 2020, Letchuman et al. 2022, Liu et al. 2023, Khalsa et al. 2020). While syngeneic mouse gliomas preserve an intact immune system but lack a human genetic context, human xenografts in immunodeficient mice retain tumour authenticity but miss the functional immune components required for immunotherapy studies (Letchuman et al. 2022, Liu et al. 2023, Khalsa et al. 2025). The proposed tiered workflow bridges this gap by preserving patient‐imposed tumour heterogeneity and incorporating functional human immune effector compartments (Chuprin et al. 2023). Looking toward future development, the preferential engagement of B cells, consistent with plasma cell expansion *in vivo* (Cakmak et al. 2025, Lauss et al. 2022), suggests the potential of inducing adaptive immune memory. However, this hypothesis requires direct assessment of memory formation and longitudinal *in vivo* follow‐up models (Phad et al. 2022).

It is also worth noting that several limitations temper the generalizability of these findings. The cohort of patient‐derived GSCs used was enriched for MES‐like transcriptional features known to possess immunogenic properties (Doucette et al. 2013), leaving it uncertain whether immune activation and tumour reprogramming would similarly occur in other GBM subtypes, such as proneural‐ or classical‐like GSCs. Moreover, the PBMC‐engrafted NSG mouse system, while enabling rapid immune reconstitution and detailed short‐term analysis of T cell dynamics, is inherently constrained by xeno‐GvHD, thereby precluding evaluation of long‐term outcomes or non‐T cell populations (Ehx et al. 2018, Ali et al. 2012). Similarly, the analysis was limited to PBMCs from healthy donors, which could introduce allo‐reactivity into the system. However, EVs are generally less immunogenic than their parent cells and show a more favourable safety profile. As EVs and EVOV are allogeneic to PBMCs to the same extent, baseline allogeneic effects are expected to be comparable. Thus, the phenotype induced by EVOV is likely due to specific responses to the altered cargo and immunogenic composition rather than differences in allogenicity (Meng et al. 2025, Kou et al. 2022). Finally, the spatial transcriptomics component was limited in the number of animals per condition; accordingly, VisiumHD results are presented as an exploratory, hypothesis‐generating dataset intended to provide spatial context for the functional and single‐cell readouts.

Notwithstanding these caveats, the proposed vaccine‐like approach provides an advanced predictive *ex vivo* assay and a mechanistically informative *in vivo* testing ground for immunotherapies, thus offering a framework for the development of personalized, clinically relevant GBM adjuvant treatment. Emerging data indicate that mRNA SARS‐CoV‐2 vaccination administered in proximity to immune checkpoint therapy is associated with improved survival, suggesting that coordinated priming and local inflammatory cues can amplify antitumour responses (Grippin et al. 2025). The modest reduction in PBMC viability observed in our model under combined conditions supports a sequential, rather than fully concurrent, regimen, in which oHSV‐1 first conditions the tumour, and EVOV is subsequently administered at a dose and interval that preserve PBMC viability while maintaining enhanced GSC cytotoxicity. Taken together, these findings support further translational evaluation of EVOV as a controllable, non‐infectious vesicle‐based immunotherapy platform designed to deliver oHSV‐1–induced immunogenic signals without relying on live viral replication.

## Author Contributions

**Klaudia Kiel**: conceptualization, methodology, investigation, writing – original draft, writing – review and editing, funding acquisition, visualization, formal analysis, validation. **Arpita Balakrishnan**: methodology, investigation, visualization, formal analysis, validation. **Aleksandra Cabaj**: methodology, formal analysis, data curation. **Luiza Stanaszek**: methodology. **Maciej Przybylski**: methodology, resources. **Dawid Stępnik**: methodology, formal analysis, visualization. **Marta Małuszek**: methodology, data curation. **Christopher Jannotta**: formal analysis, visualization. **Chiara Zoe Linke**: formal analysis. **Joanna Suszyńska‐zajczyk**: methodology. **Monika Knaur**: methodology, visualization, formal analysis. **Agnieszka Szczypiorowska**: methodology. **Szymon Hryhorowicz**: methodology, resources, data curation. **Bianka Świderska**: methodology, resources, formal analysis, data curation. **Katarzyna Piwocka**: writing – review and editing, resources, funding acquisition. **Alexander Lee Ling**: formal analysis, writing – review and editing. E. **Antonio Chiocca**: resources, writing – review and editing, funding acquisition. **Agnieszka Bronisz**: conceptualization, investigation, writing – original draft, writing – review and editing, funding acquisition, resources, supervision. **Jakub Godlewski**: writing – review and editing, supervision, funding acquisition, resources, project administration, writing – original draft.

## Funding

The authors declare that financial support was received for the research and publication of this article by: The National Science Centre, Poland: 2021/41/N/NZ6/02682 (to K.K.), 2022/45/B/NZ5/04226 (to A.B.*), 2020/39/B/NZ5/02893, and 2021/41/B/NZ6/02436 (to J.G.); Polish National Agency for Academic Exchange: PPN/PPO/2019/1/00001 (to J.G.); MMRI PAS intramural grant: FBW‐045 (to K.K.); Medical Research Agency 2024/ABM/08/00106/P/02 (to J.G.); National Institutes of Health: National Cancer Institute: P01 CA163205 and P01 CA236749 (to E.A.C.), and National Institute of Neurological Disorders and Stroke: R01NS110942 (to E.A.C.). The research was also supported by the NEBI Project—National Center for Advanced Analysis of Biological and Biomedical Imaging, obtained under the Intelligent Development Operational Program, number POIR.04.02.00‐00‐C004/19 (to K.P.), in the framework of measure 4.2 Development of modern research infrastructure of the science sector of the Operational Program, Intelligent Development 2014–2020 co‐financed by the European Regional Development Fund.

## Ethics Statement

All animal procedures for the care and use of laboratory animals were conducted in accordance with the guidelines of our institutions and the general regulations governing animal experimentation.

## Conflicts of Interest

E. Antonio Chiocca is an advisor to Bionaut Labs, Seneca Therapeutics, Calidi Biotherapeutics, and ReIgnite Therapeutics; he has equity options in Bionaut Laboratories, ReIgnite Therapeutics, Seneca Therapeutics, and Ternalys Therapeutics; he is co‐founder and on the board of directors of Ternalys Therapeutics. Patents related to oHSV and CAN‐3110 are held by Brigham and Women's Hospital, with E. Antonio Chiocca named as a co‐inventor. These patents have been licensed to Candel Therapeutics. Candel distributes present and future milestone license fees and future royalty fees to Brigham and Women's Hospital.

## Supporting information




**Supporting Information**: jev270389‐sup‐0001‐FigureS1‐S20.docx

**Supporting Information**: jev270389‐sup‐0002‐SuppMat.pdf

**Supporting Information**: jev270389‐sup‐0003‐SuppMat.docx

**Supporting Information**: jev270389‐sup‐0004‐SuppMat.xlsb

## Data Availability

The DIA‐MS proteomics data have been deposited in PRIDE under accession number PXD054707, the scRNA‐seq data in EGA under accession number EGAS50000001124, and the VisiumHD data in GEO under accession number GSE308834. The data that support the findings of this study are openly available through the ProteomeXchange Consortium via the jPOST partner repository at https://repository.jpostdb.org/entry/JPST004780, with the dataset identifiers PXD081298 and JPST004780 (Okuda et al. 2025). Original Western blot images are available from the Corresponding Author upon request. No original code was generated in this study. All numerical data reported in this paper are available in the attached Excel spreadsheets, and peptidomics spectra are shown in the Supplemental_MS_Spectra dataset (see Supplementary Source Dataset).
