# Supplementary material for: Oncolytic Virus‐Engineered Extracellular Vesicles Rewire Glioblastoma Immunity
Source: J Extracell Vesicles. 2026 Oct 21;15(10):e70389. doi: 10.1002/jev2.70389 (PMC13687664; doi:10.1002/jev2.70389)

File Description:

Representative MS/MS spectra of differentially abundant MHC-associated peptides. The spectra shown correspond to the highest-confidence identification (lowest q-value) for each differentially abundant peptide identified in the MHC peptidomics analysis. Matched b- and y-type fragment ions, including neutral-loss ions (−H_2O and −NH_3), are annotated. The precursor m/z, charge state, mass error (Δm), XCorr score, and q-value are reported for each spectrum.

ELSDFISYLQR - PDIA3

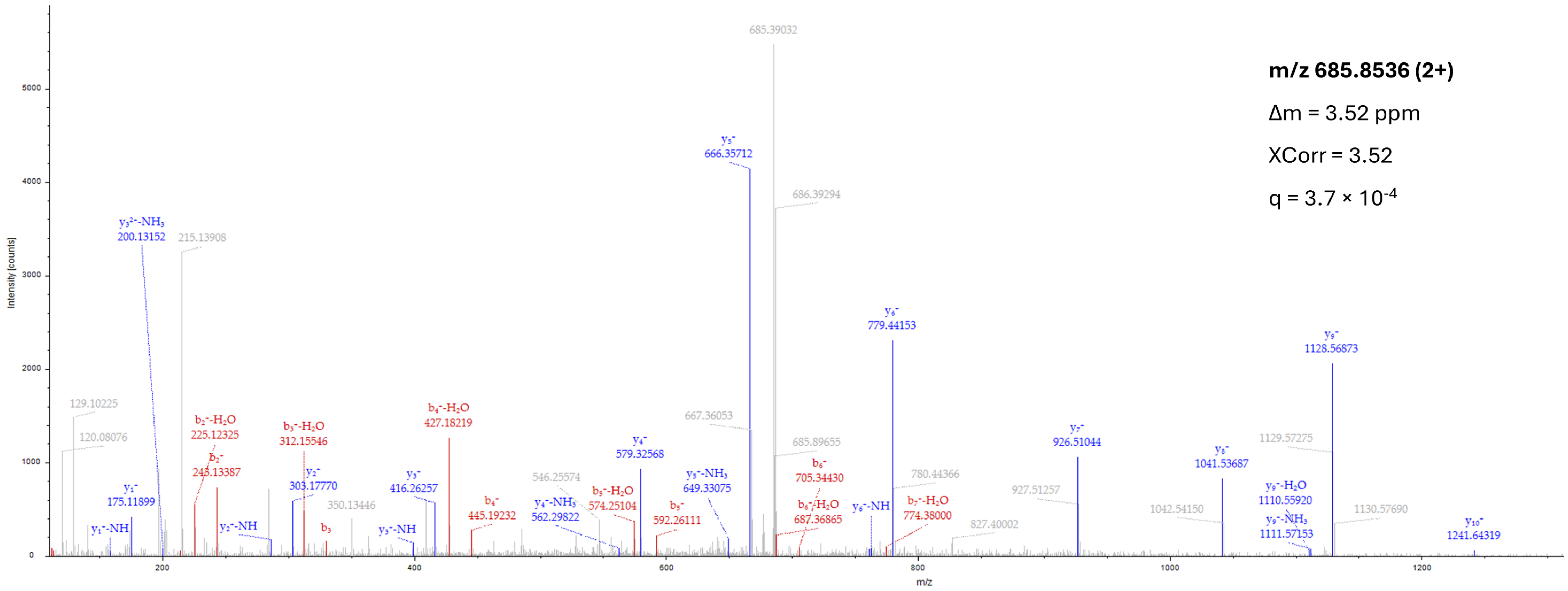

Fragment ions

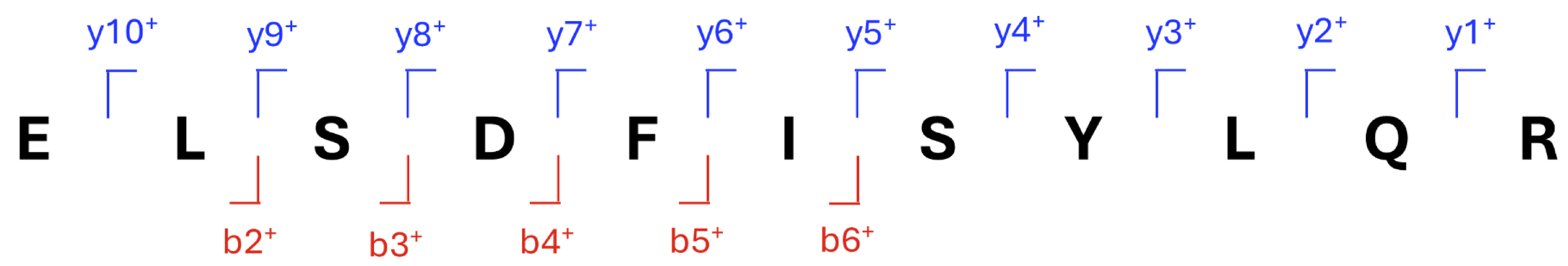

Neutral-loss fragment ions

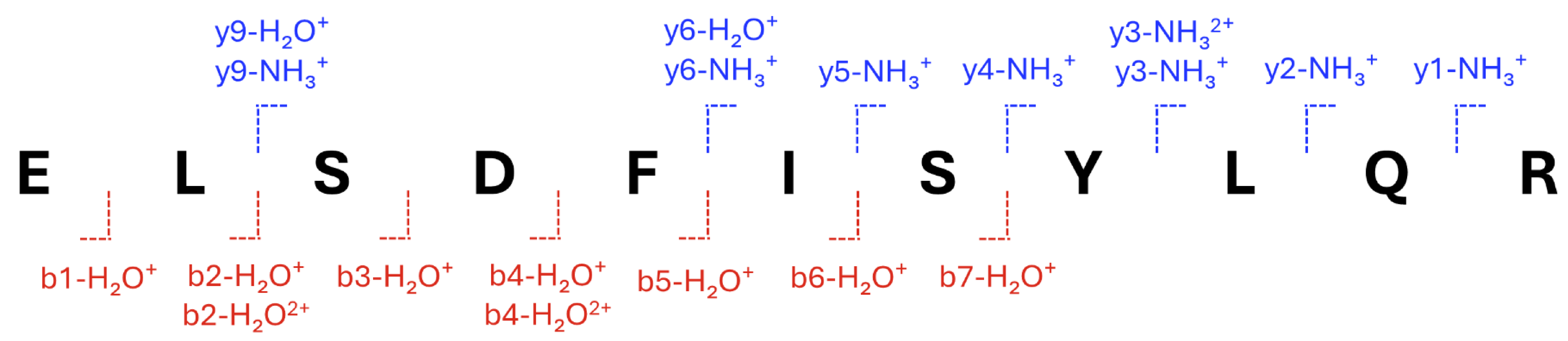

DINTDGAVNF - S100A8

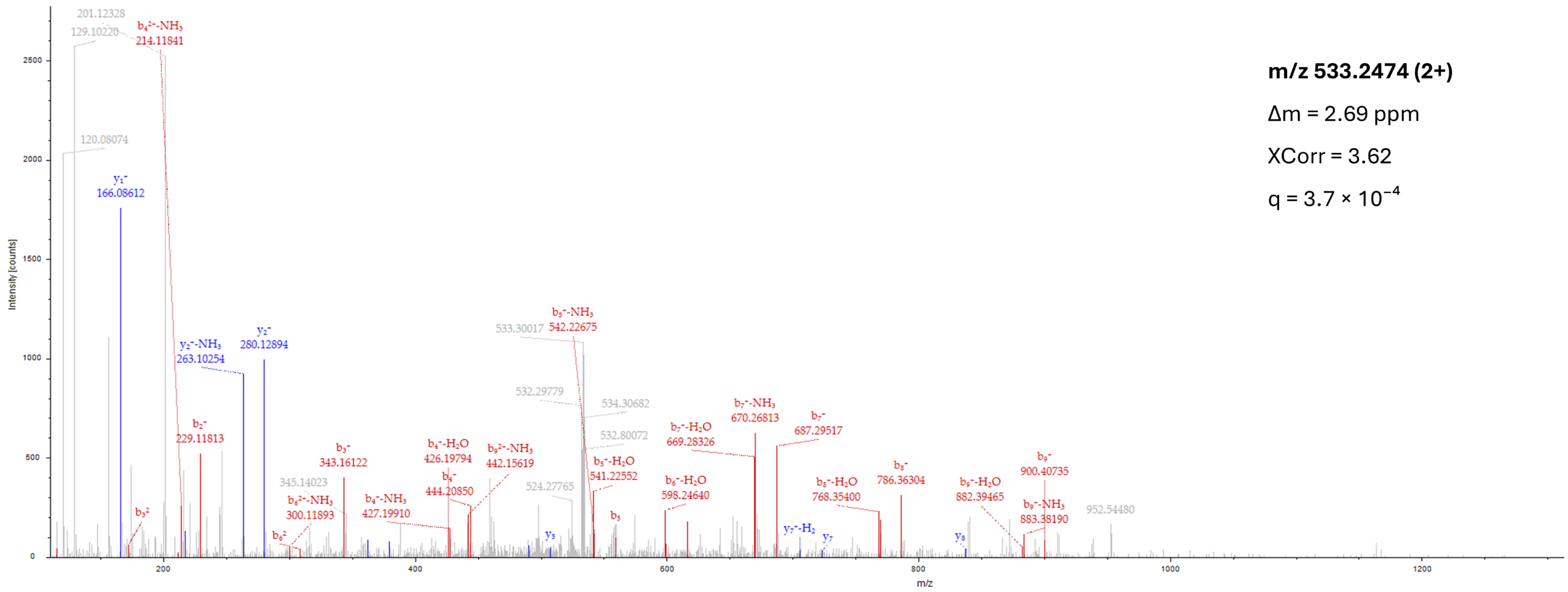

Fragment ions

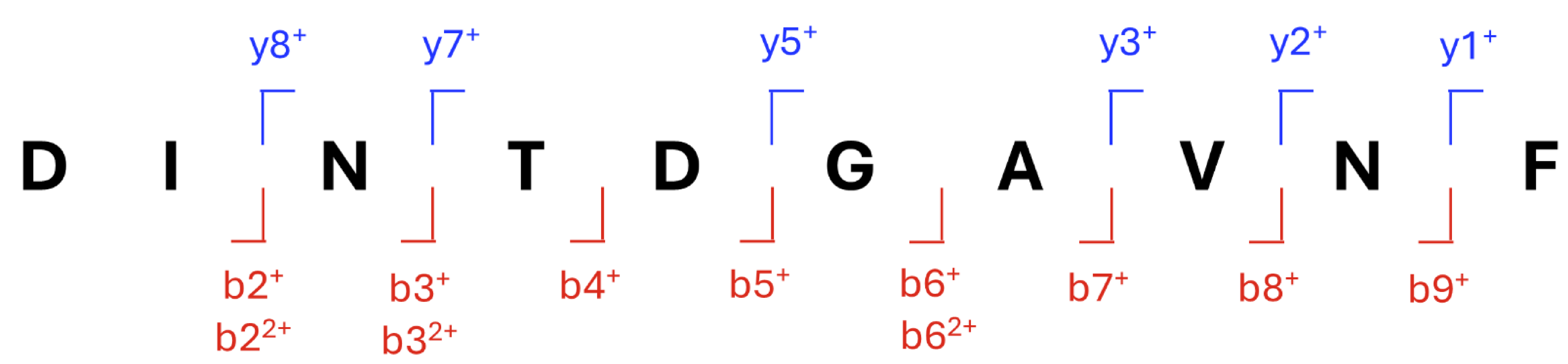

Neutral-loss fragment ions

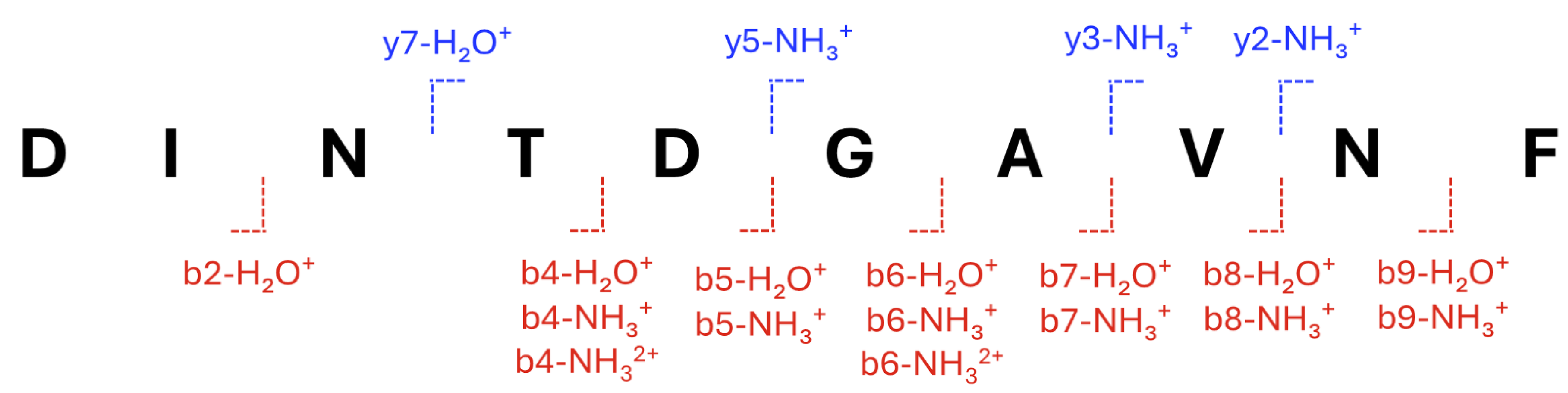

DAERDALNIETAIKTK - ANXA2

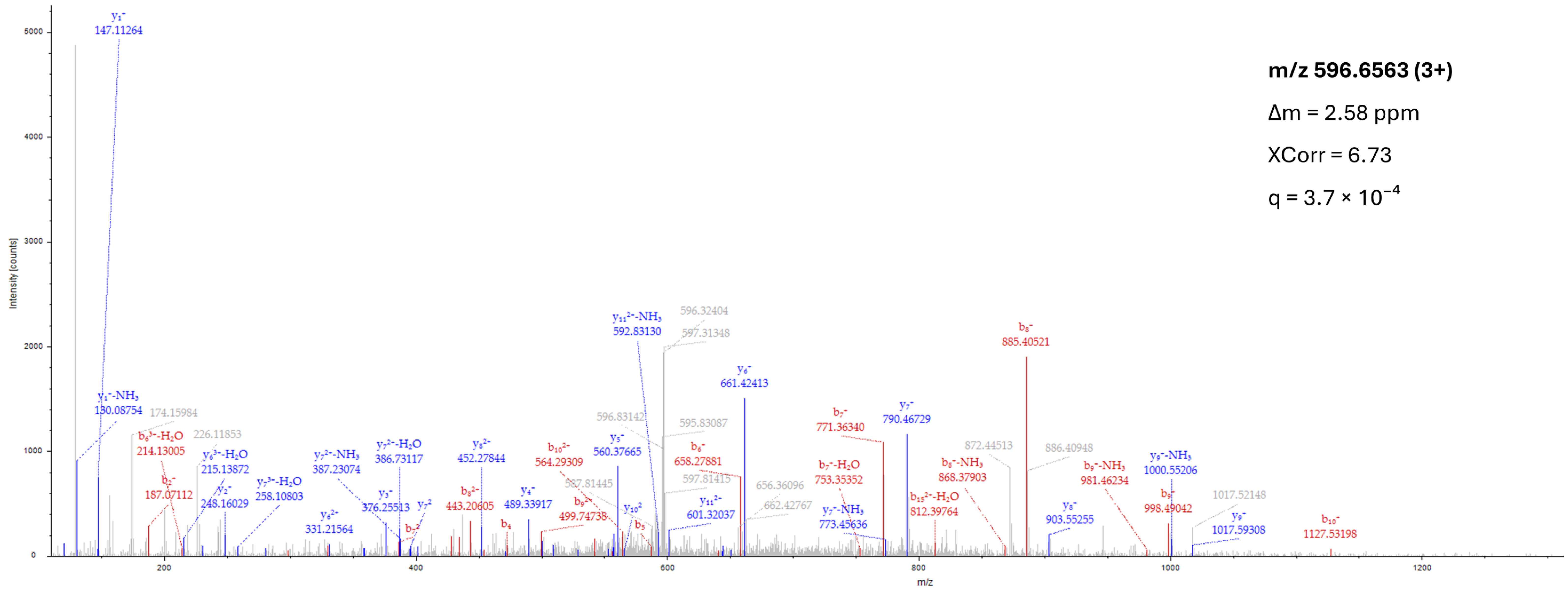

Fragment ions

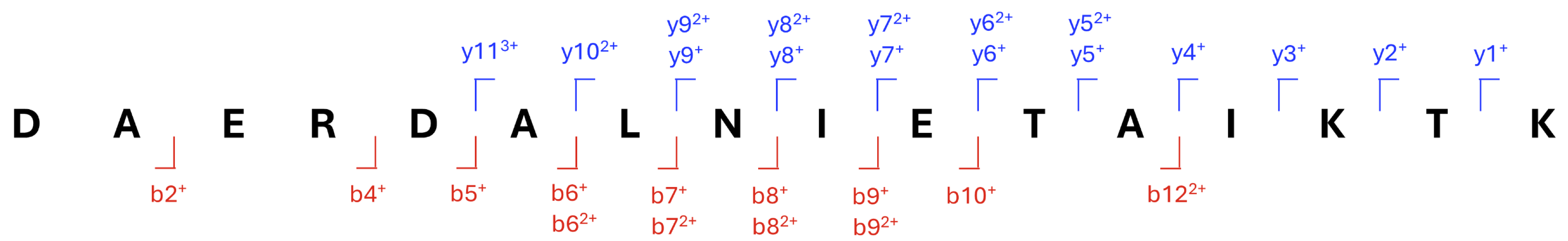

Neutral-loss fragment ions

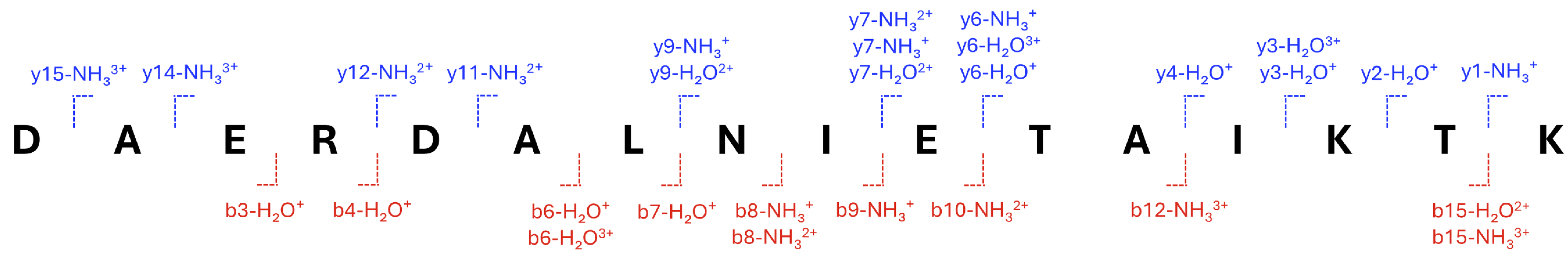

GGVAAGVAVLDNPYPV - GSS

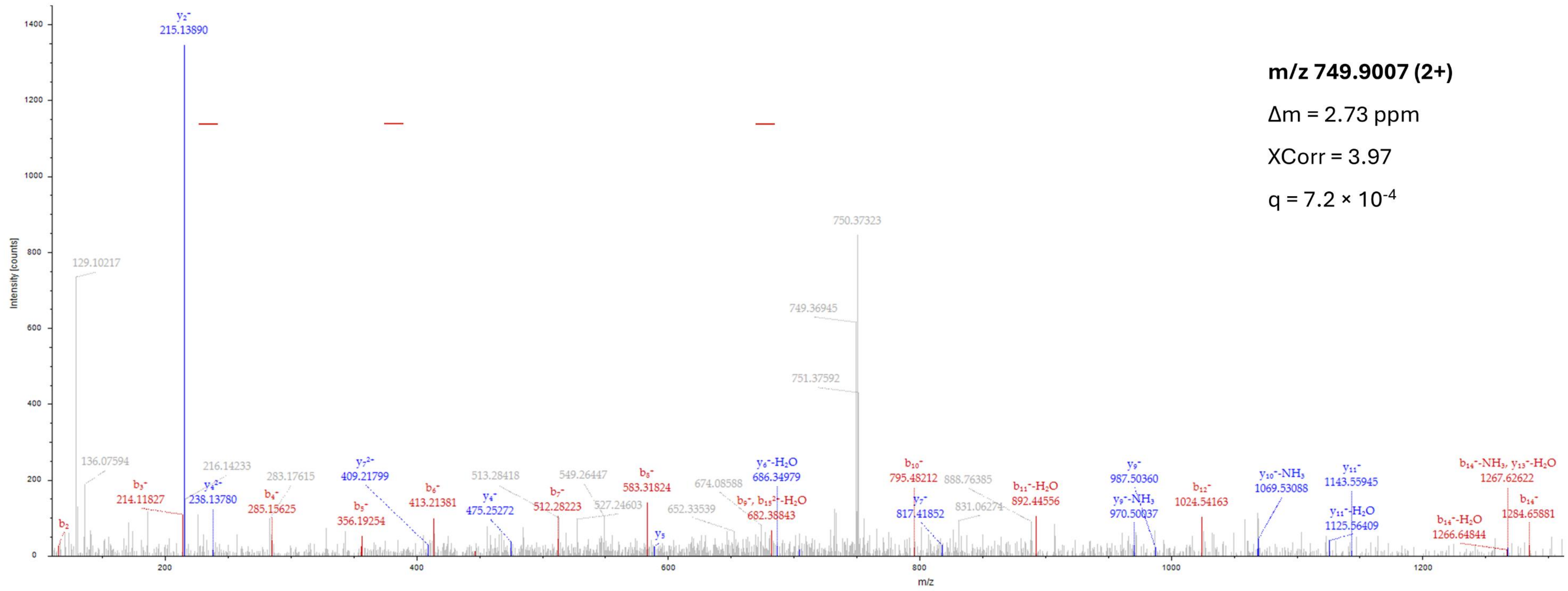

Fragment ions

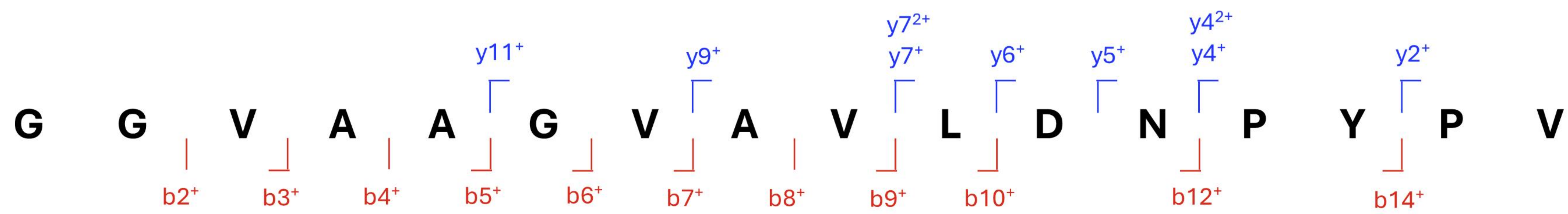

Neutral-loss fragment ions

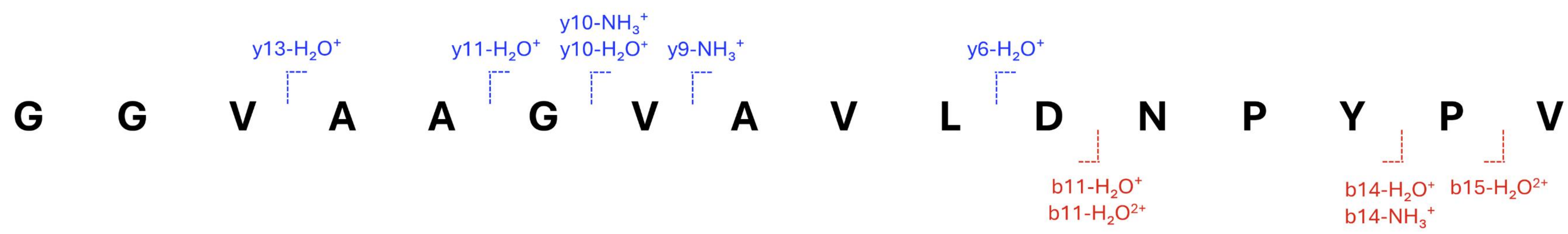

FFDFIGR - TRMT44

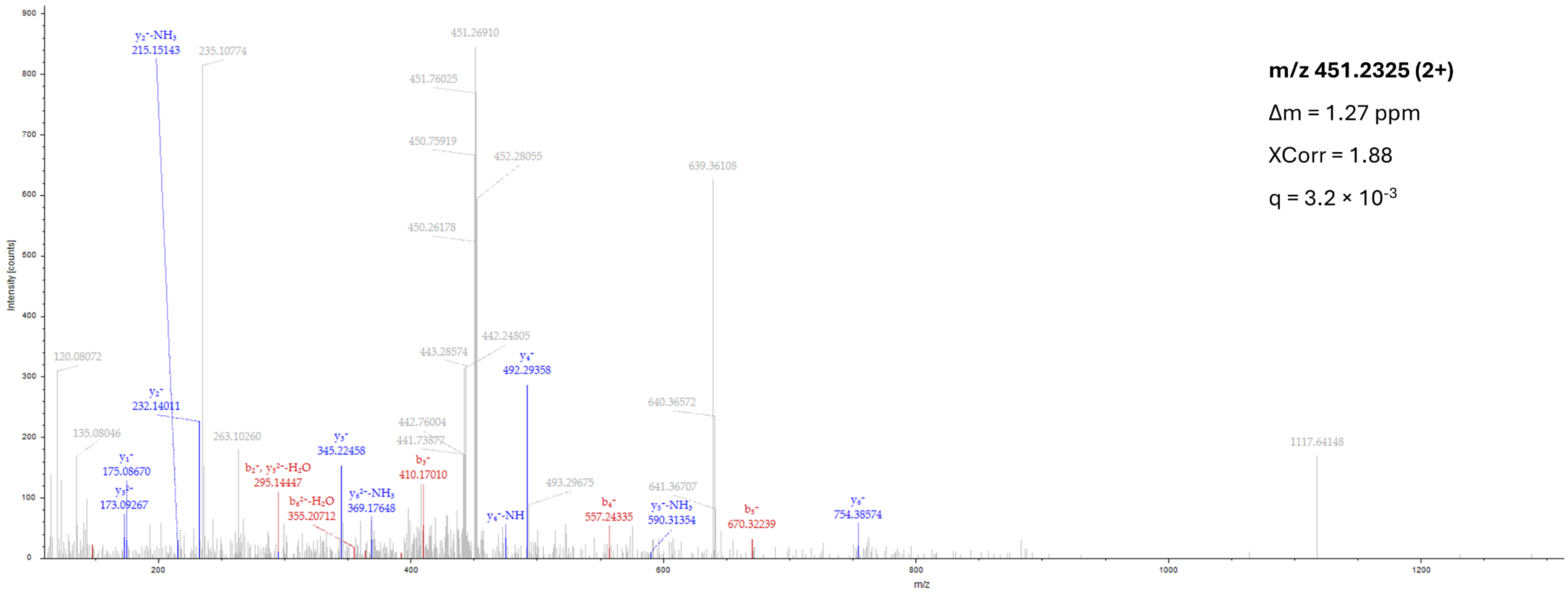

m/z 451.2325 (2+)

Δm = 1.27 ppm

XCorr = 1.88

q = 3.2×10^{-3}

Fragment ions

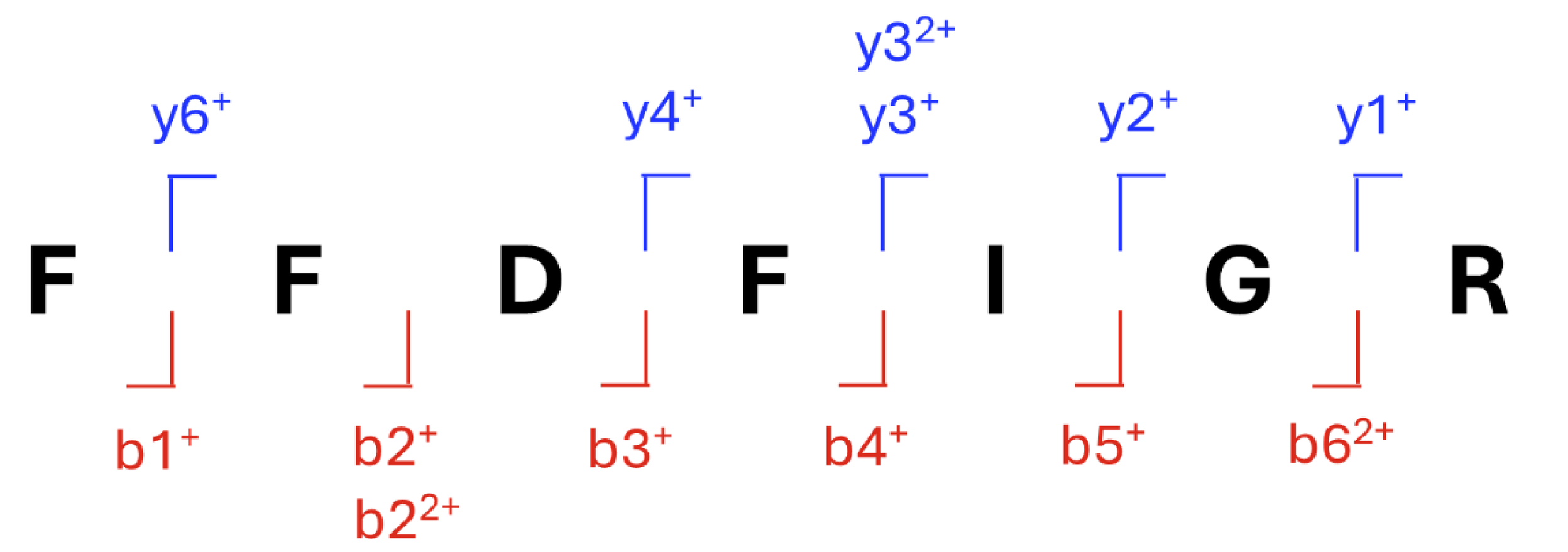

Neutral-loss fragment ions

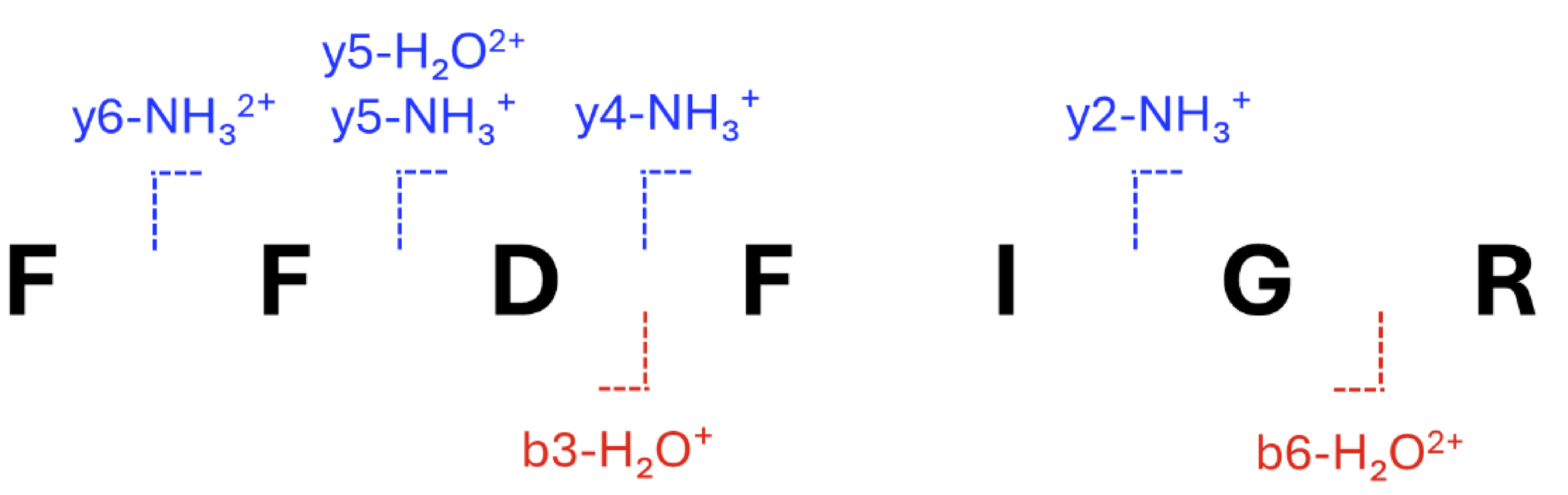

MQQKLVDQNIFS - CTSD

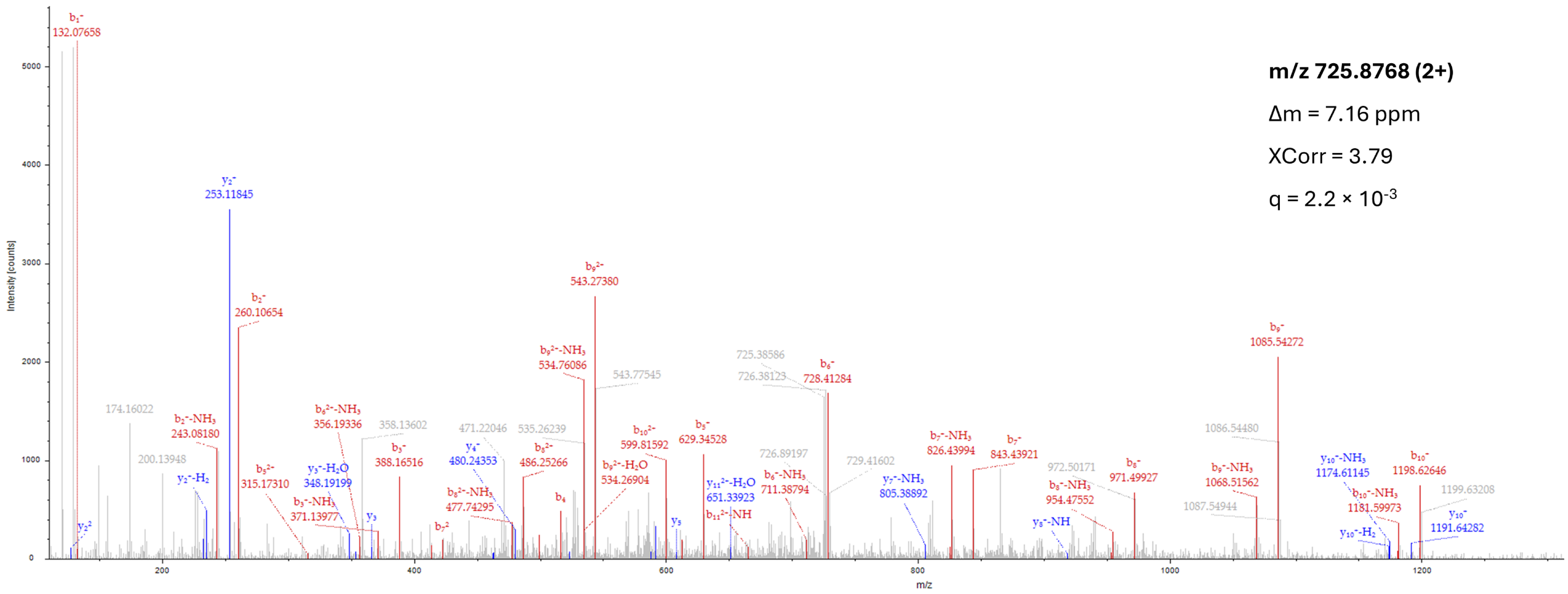

m/z 725.8768 (2+)

Δm = 7.16 ppm

XCorr = 3.79

q = 2.2×10^{-3}

Fragment ions

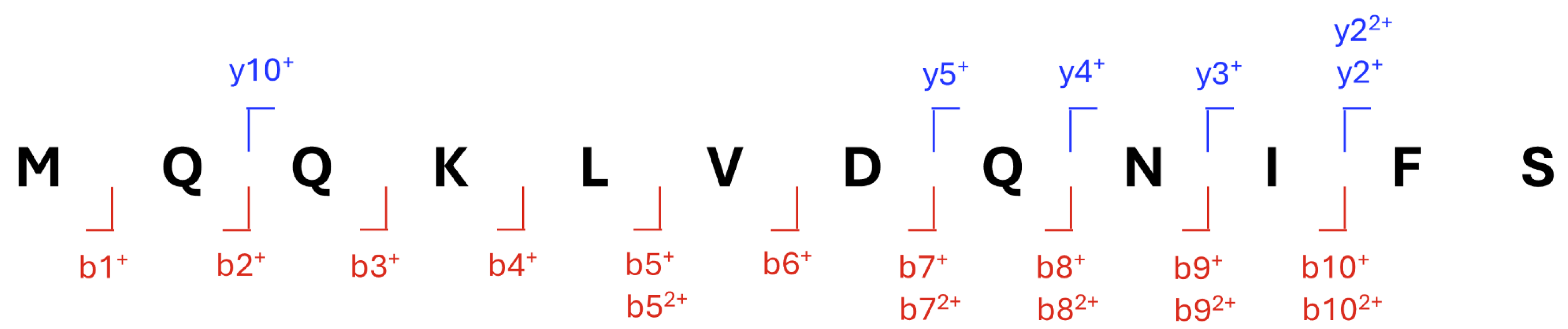

Neutral-loss fragment ions

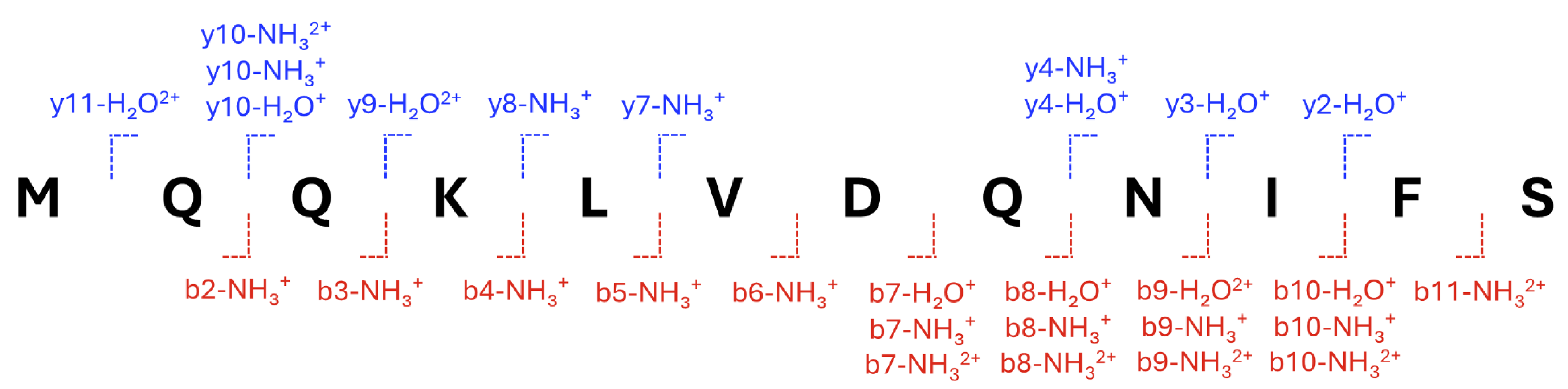

DPFNPFELTNH - CTSC

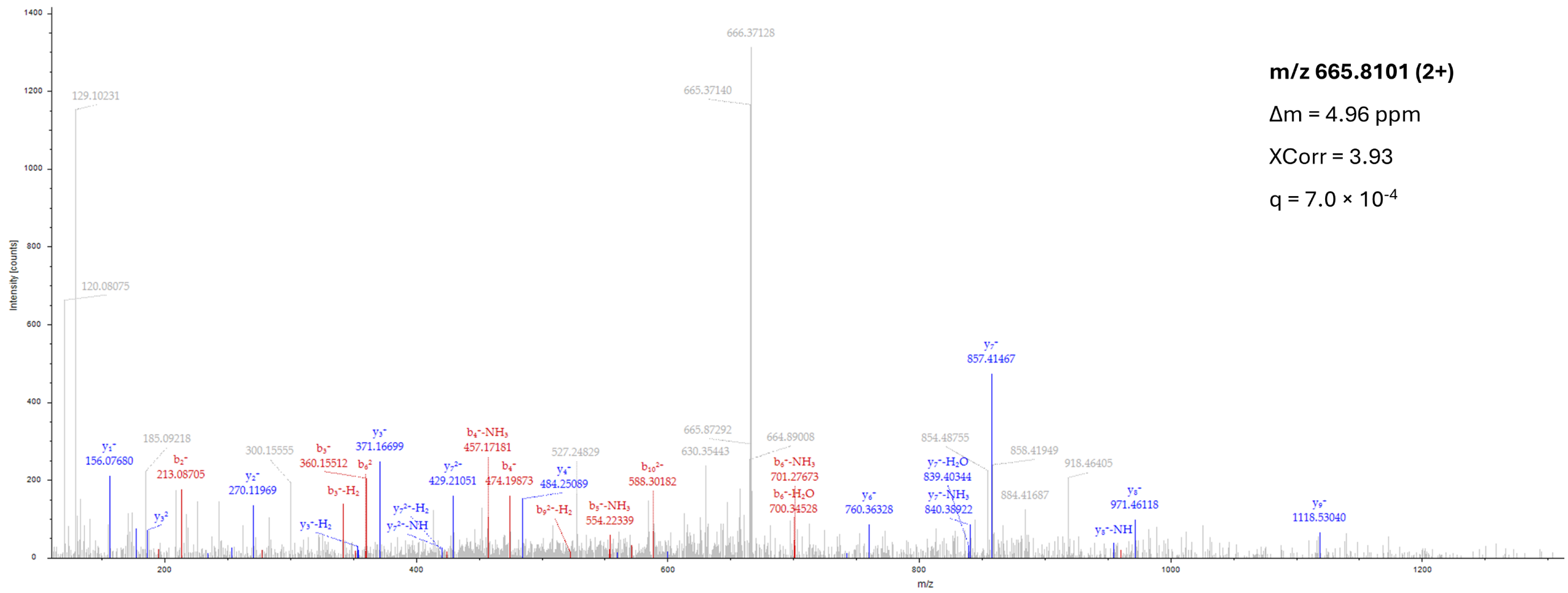

Fragment ions

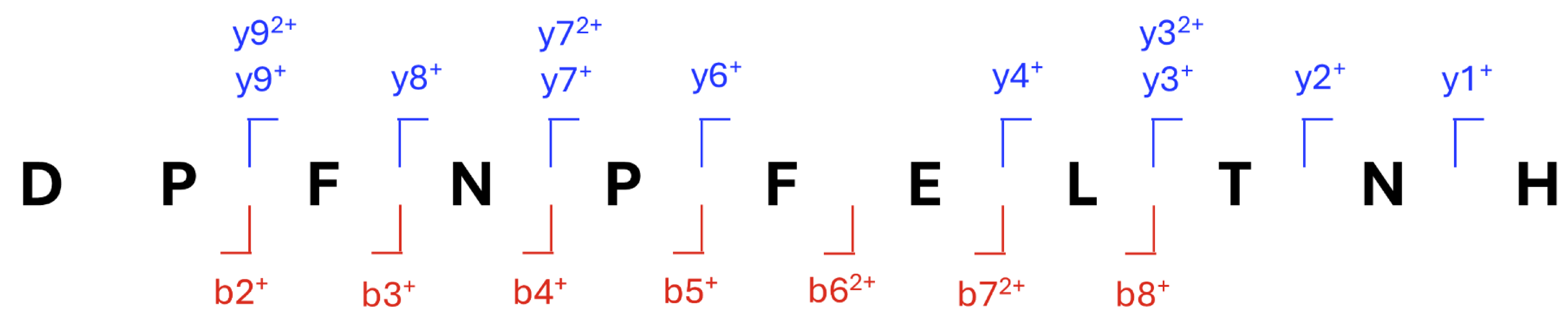

Neutral-loss fragment ions

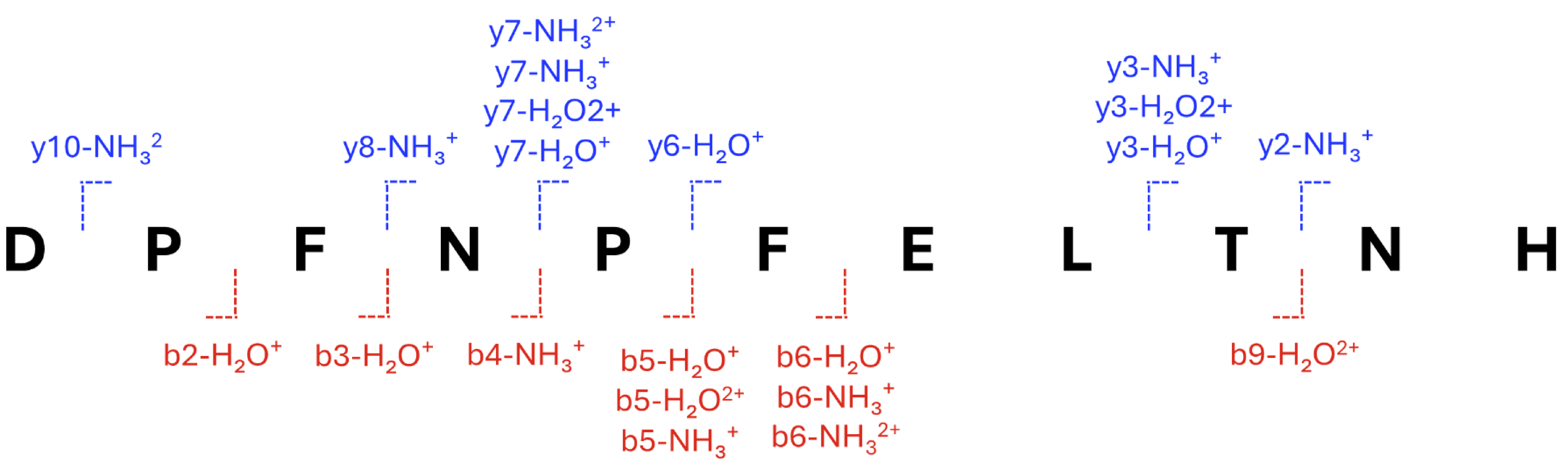

PPLIENSVSI - ALOXE3

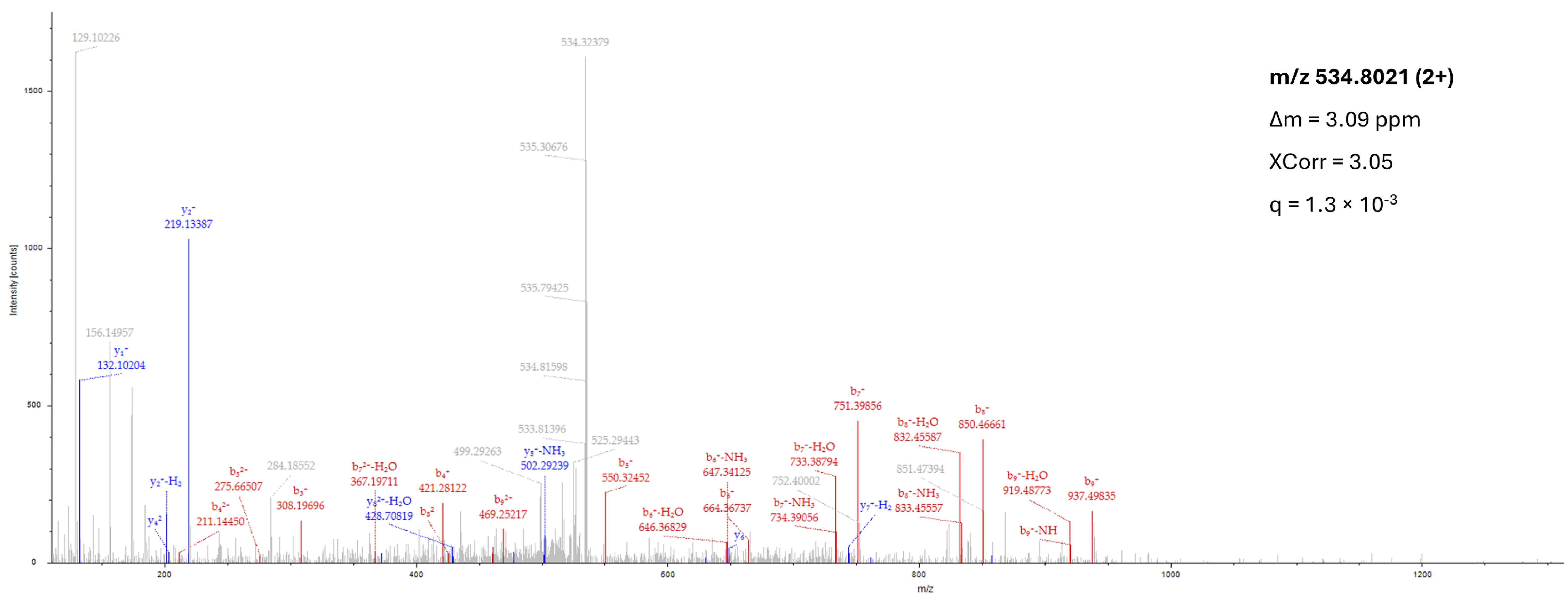

Fragment ions

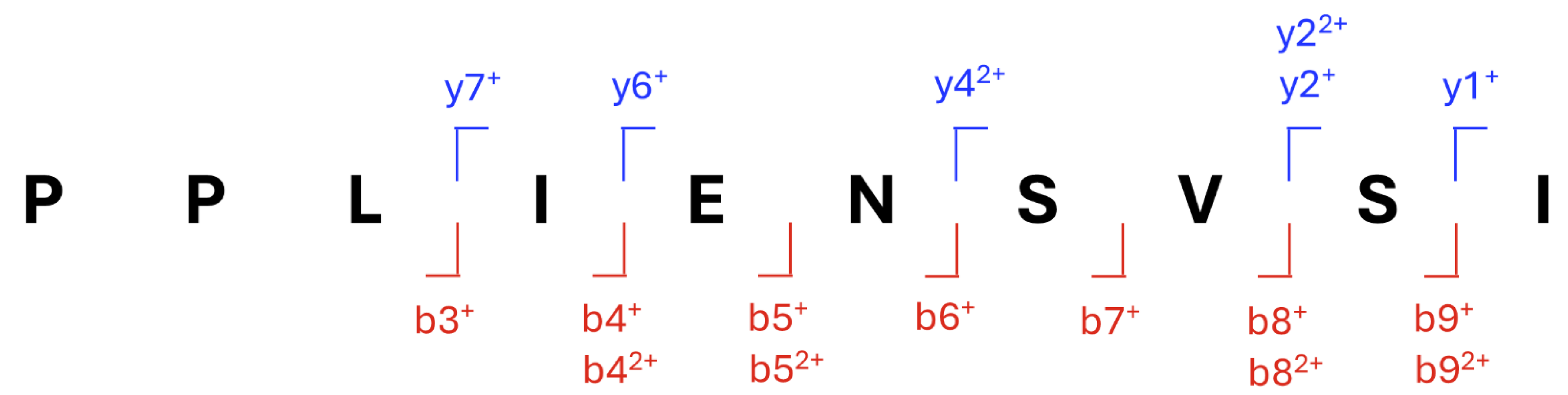

Neutral-loss fragment ions

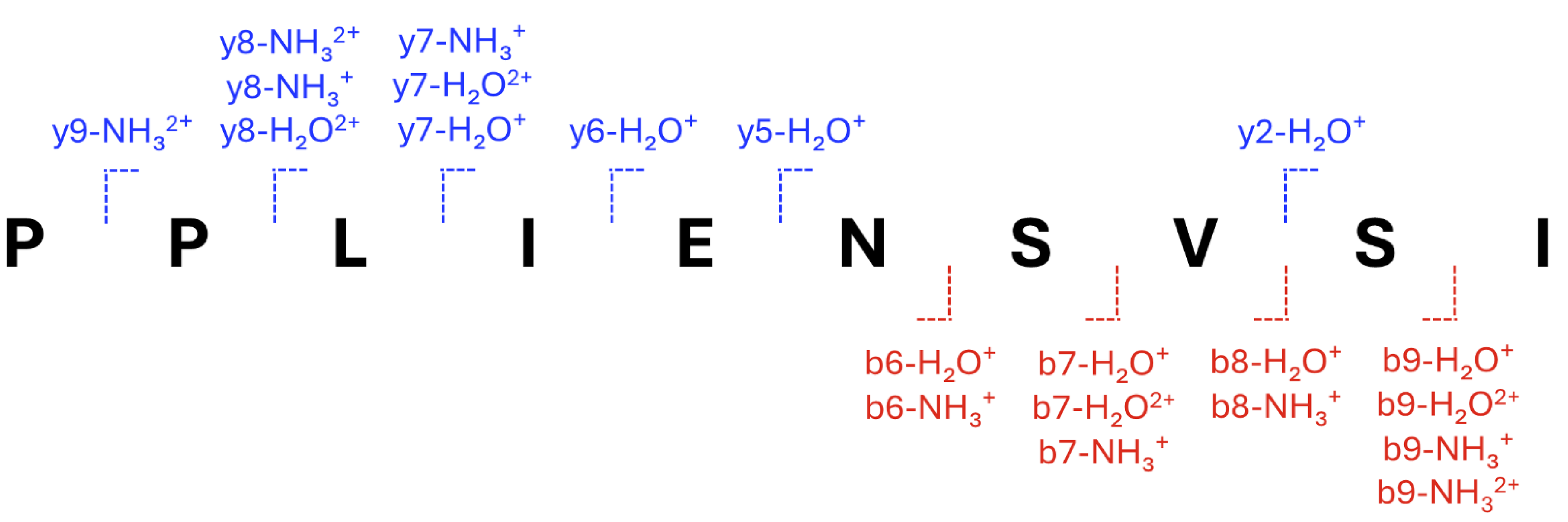

EADDIVNWLK - P4HB

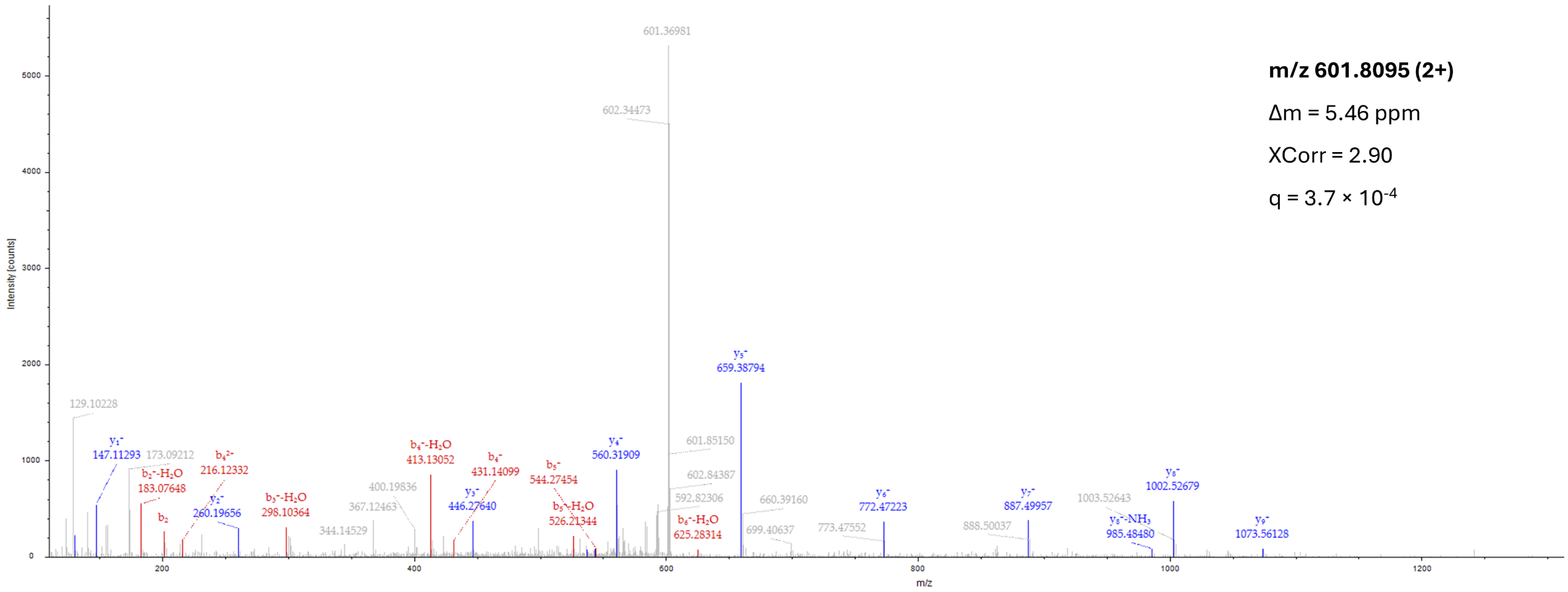

m/z 601.8095 (2+)

Δm = 5.46 ppm

XCorr = 2.90

$q = 3.7 \times 10^{-4}$

Fragment ions

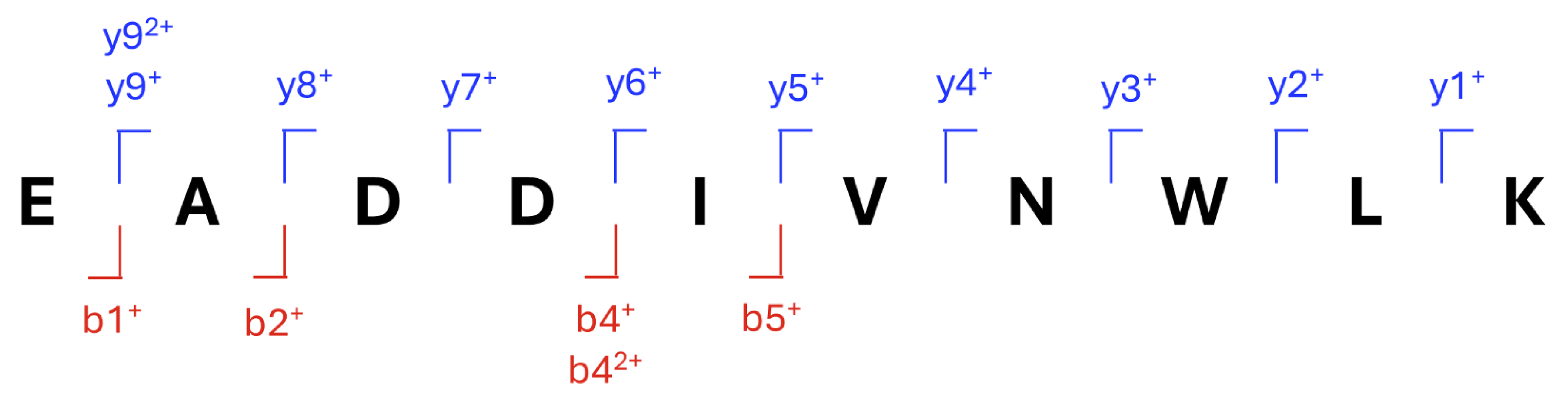

Neutral-loss fragment ions

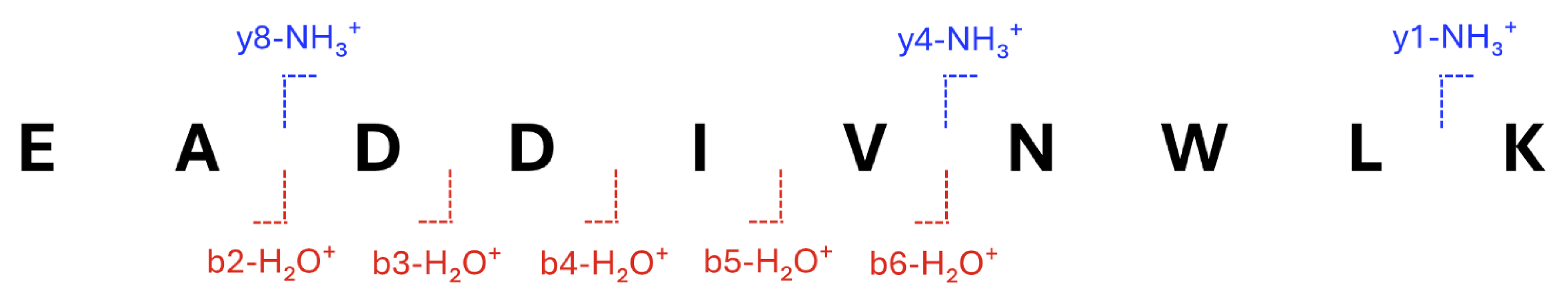

FVFSLVDAMNGK - MDH2

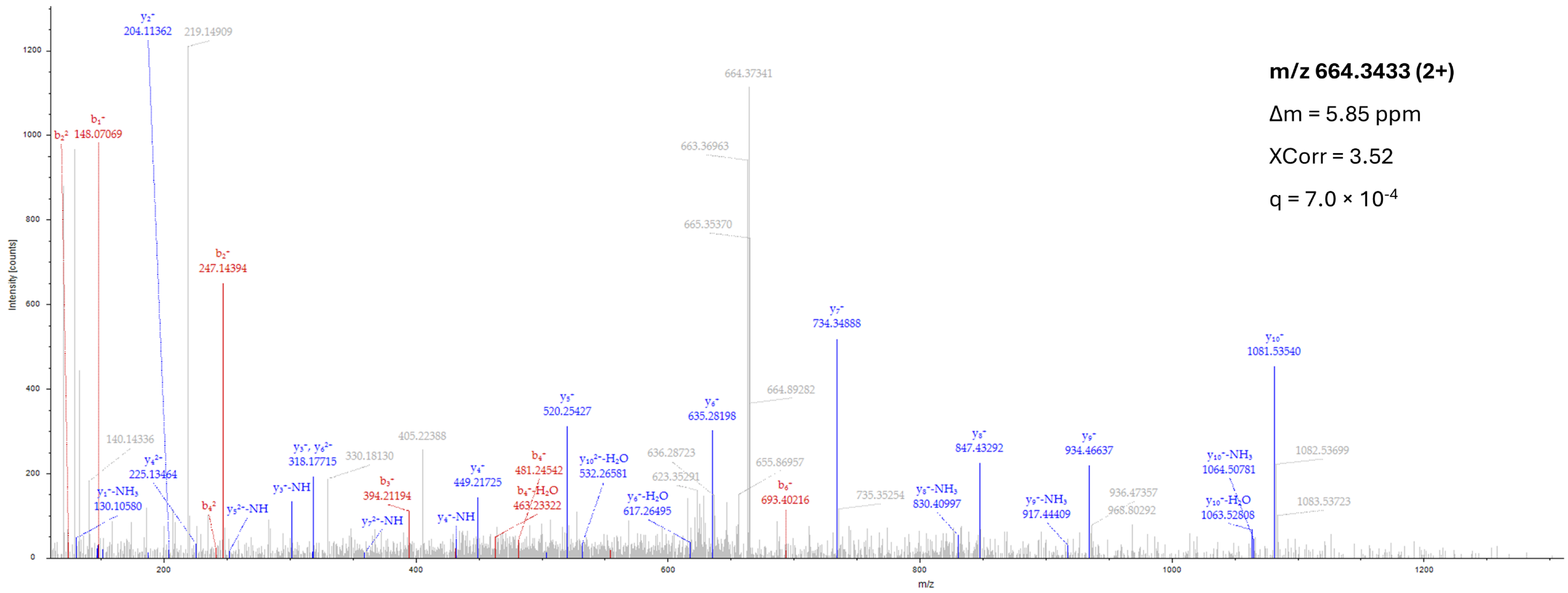

m/z 664.3433 (2+)

Δm = 5.85 ppm

XCorr = 3.52

$q = 7.0 \times 10^{-4}$

Fragment ions

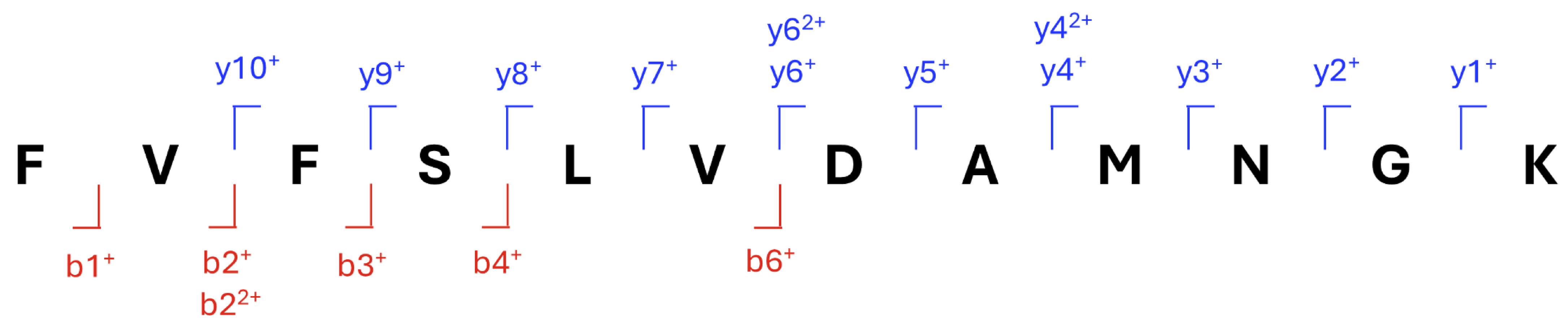

Neutral-loss fragment ions

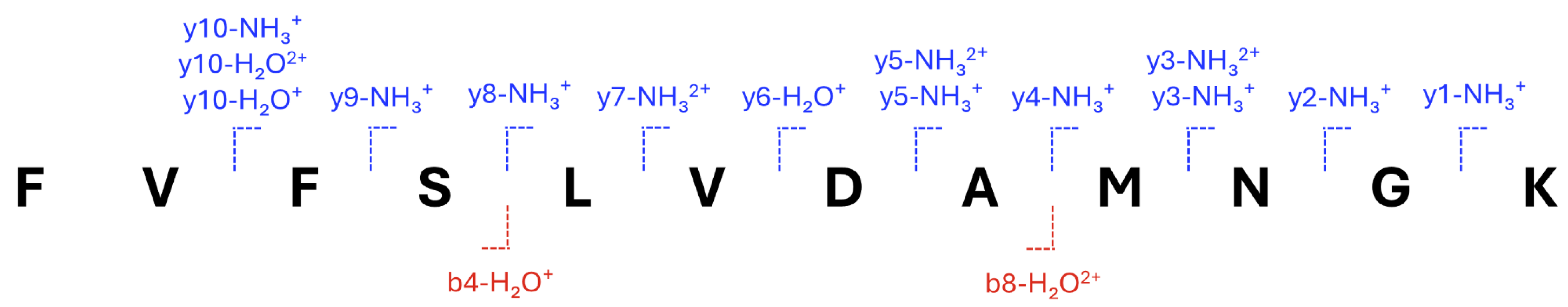

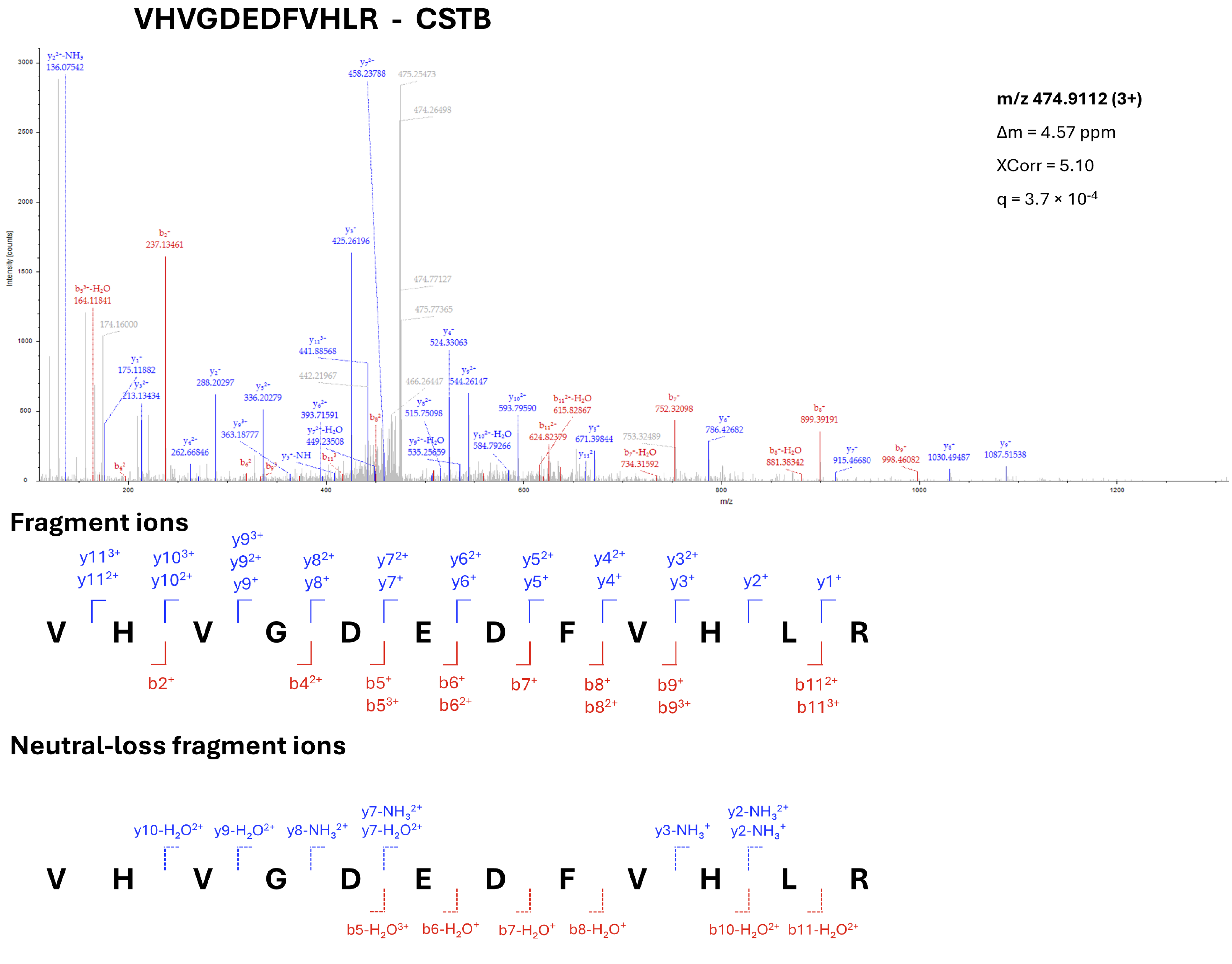
VHVGDEDFVHLR - CSTB
m/z 474.9112 (3+)
Δm = 4.57 ppm
XCorr = 5.10
q = 3.7 × 10-4
Fragment ions
Neutral-loss fragment ions

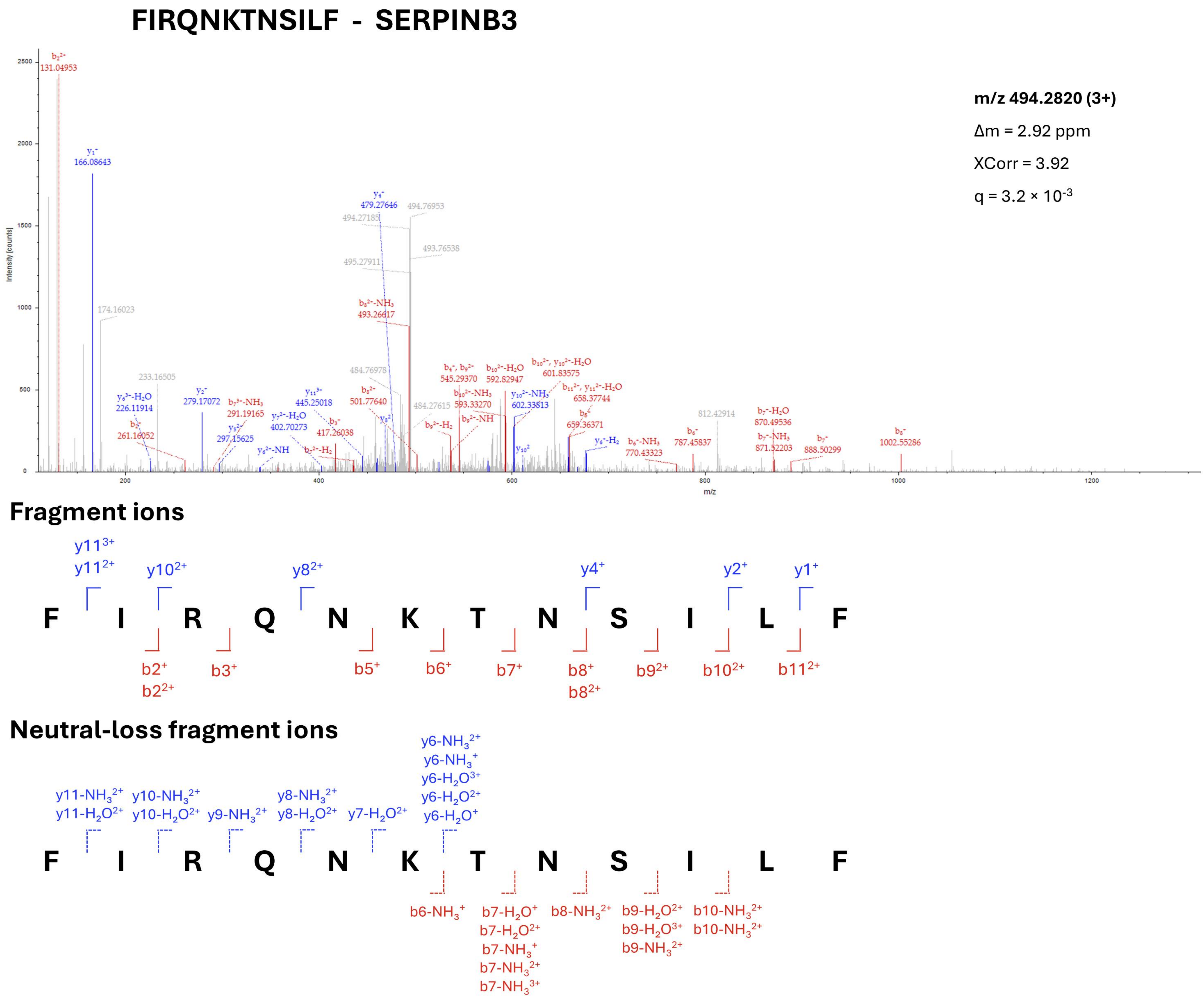
FIRQNKTNSILF - SERPINB3
m/z 494.2820 (3+)
Δm = 2.92 ppm
XCorr = 3.92
q = 3.2 × 10-3
Fragment ions
Neutral-loss fragment ions

GFSGLDGAK - COL1A1

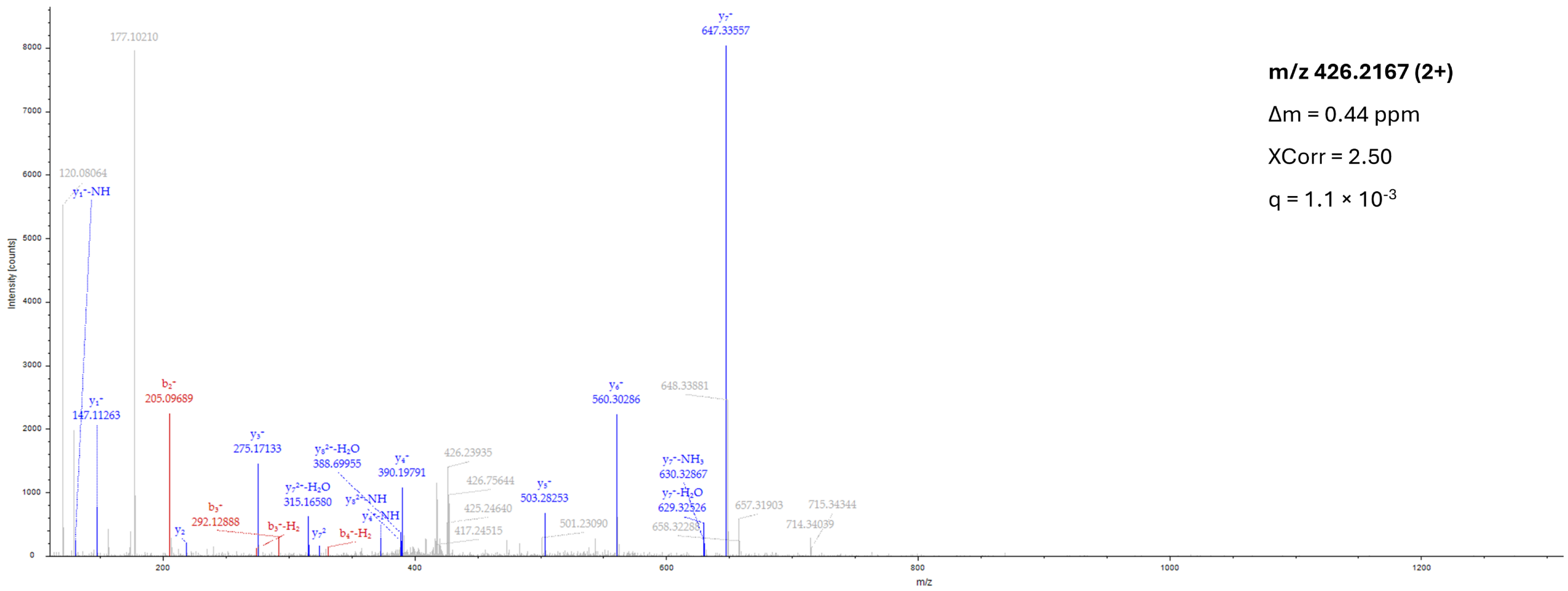

Fragment ions

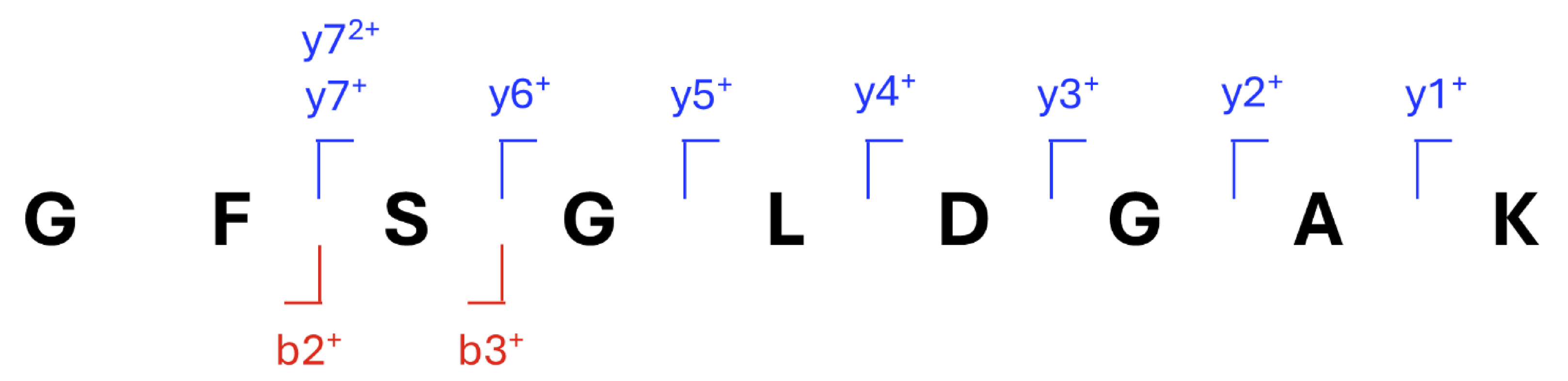

Neutral-loss fragment ions

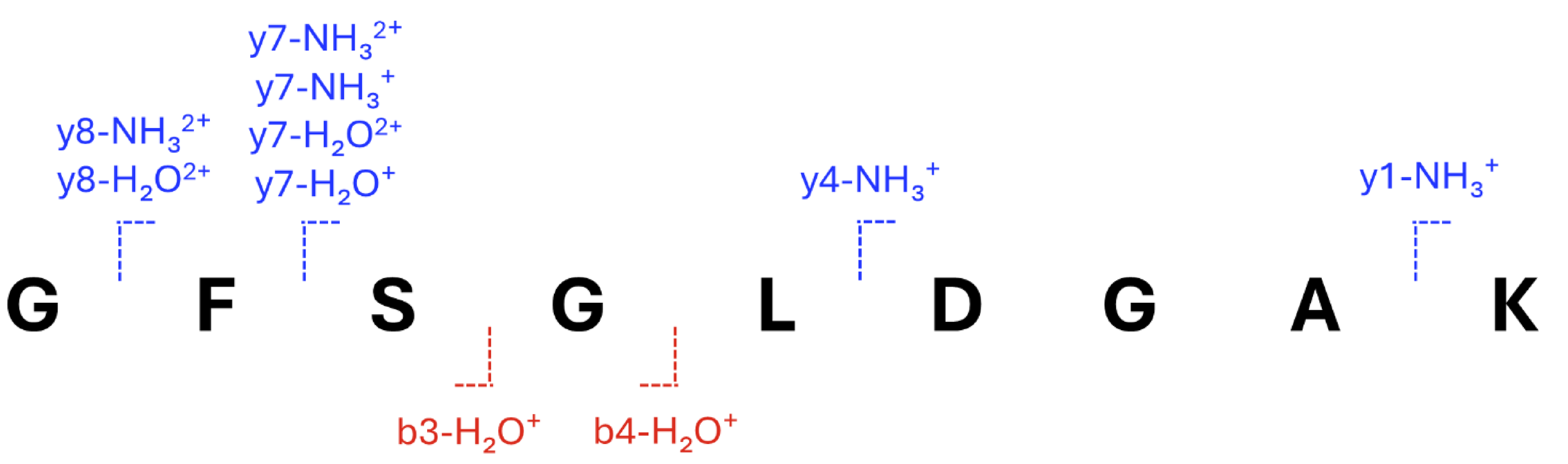

ERNIETIINTF - S100A9

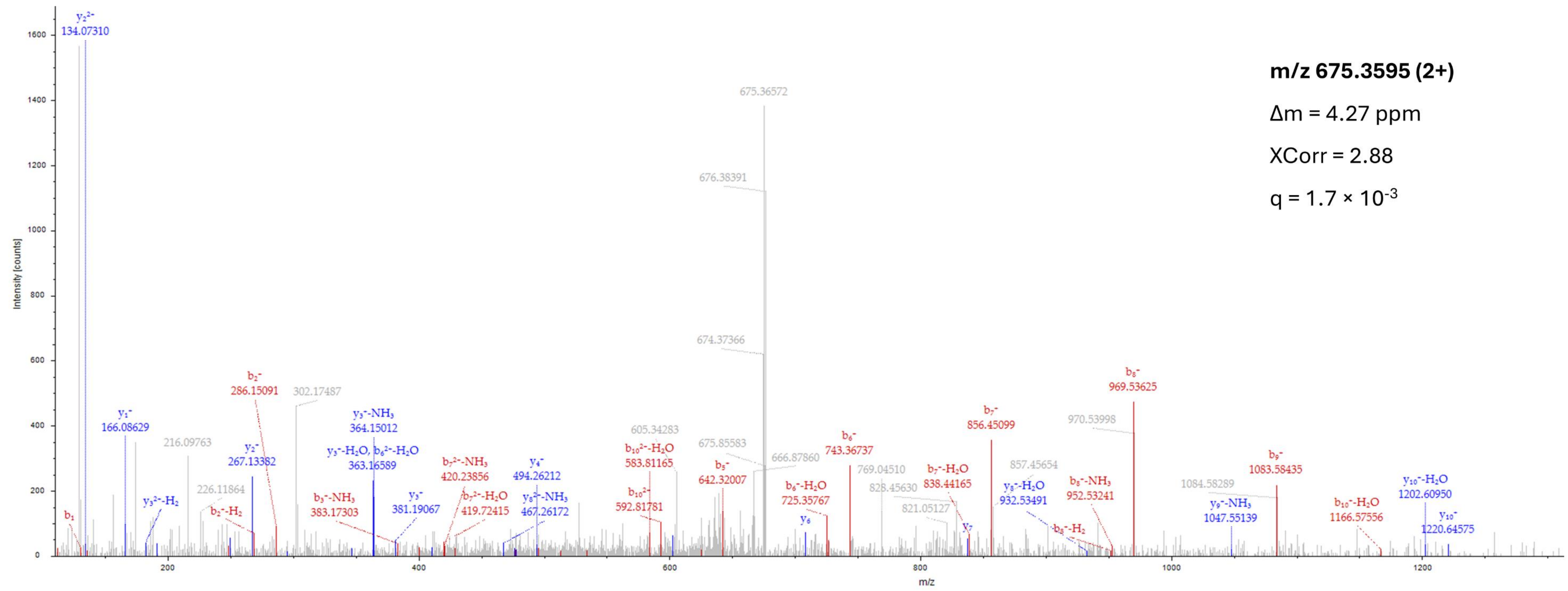

Fragment ions

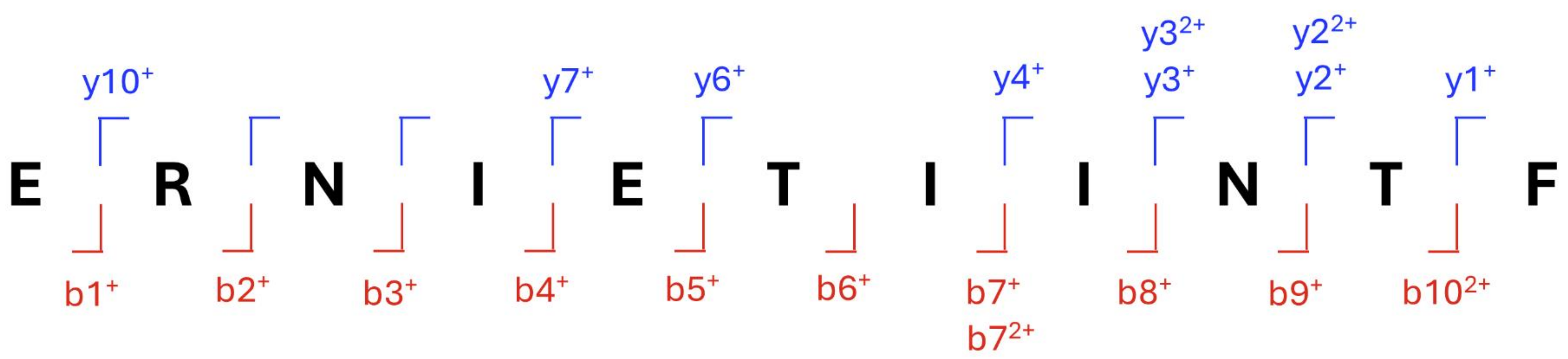

Neutral-loss fragment ions

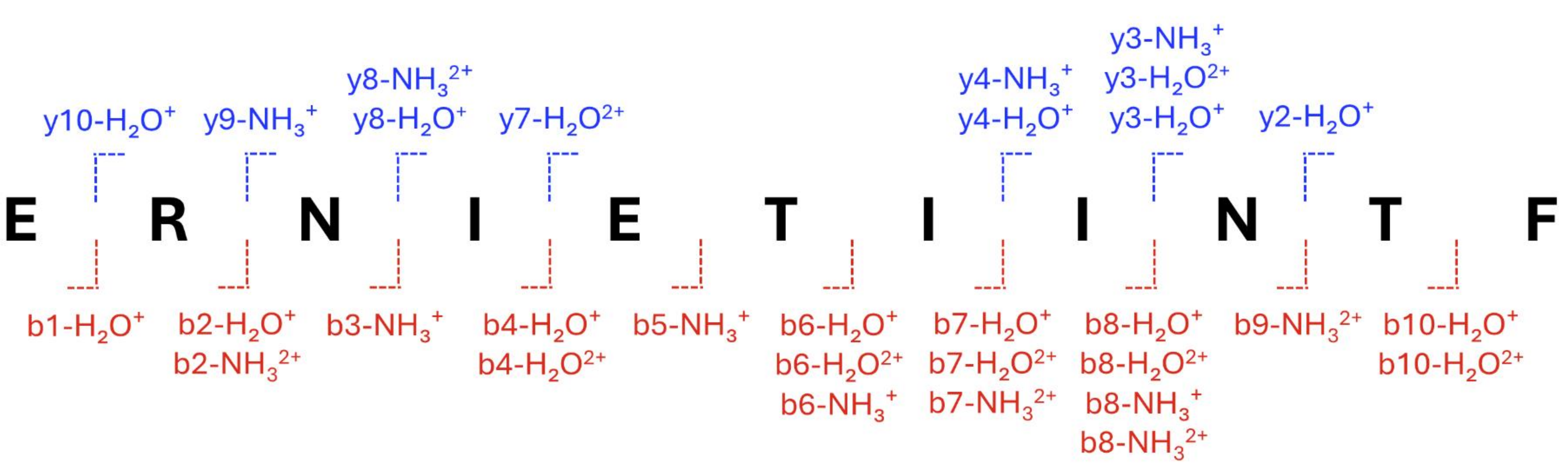

LLLPGELAK - H2BC15

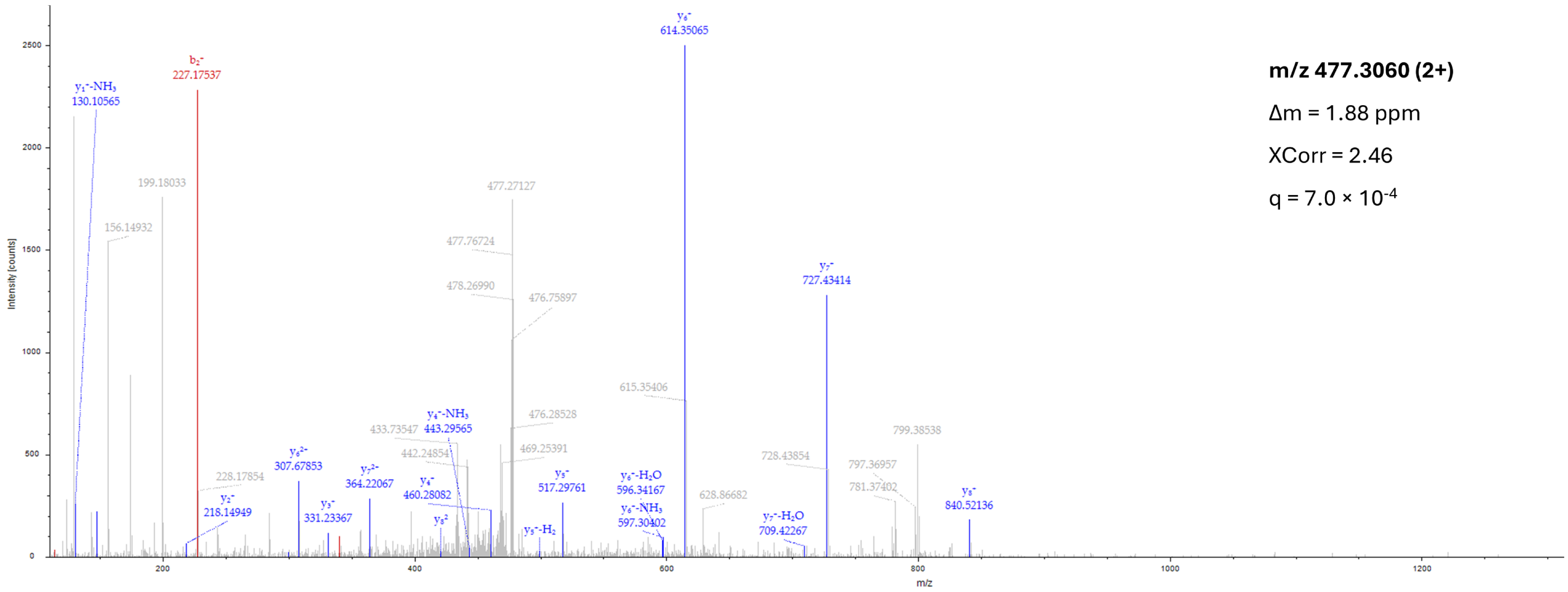

m/z 477.3060 (2+)

Δm = 1.88 ppm

XCorr = 2.46

q = 7.0 × 10^{-4}

Fragment ions

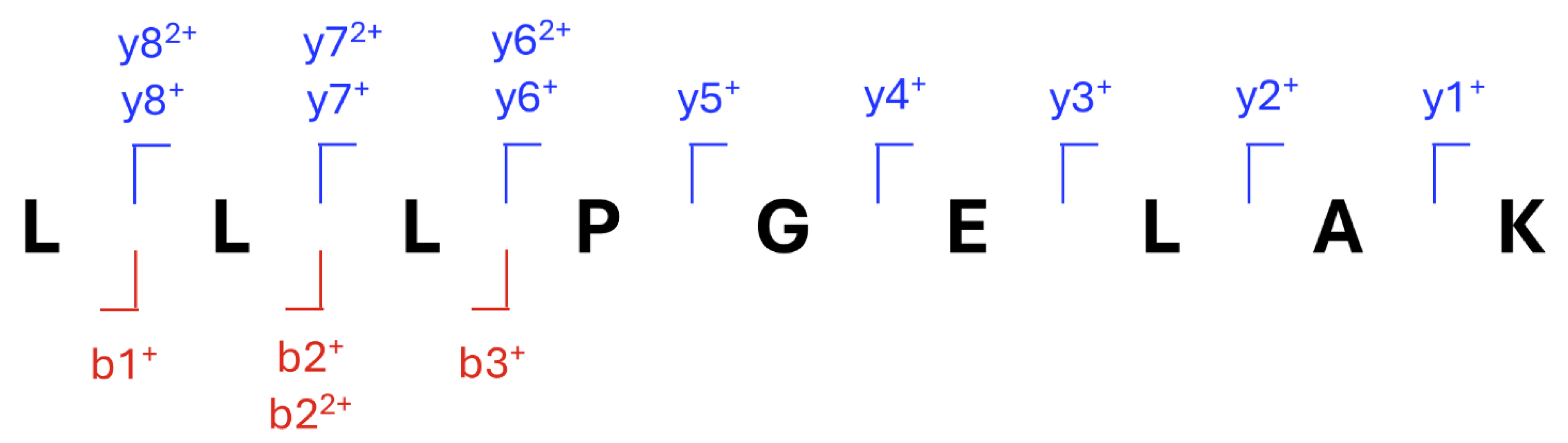

Neutral-loss fragment ions

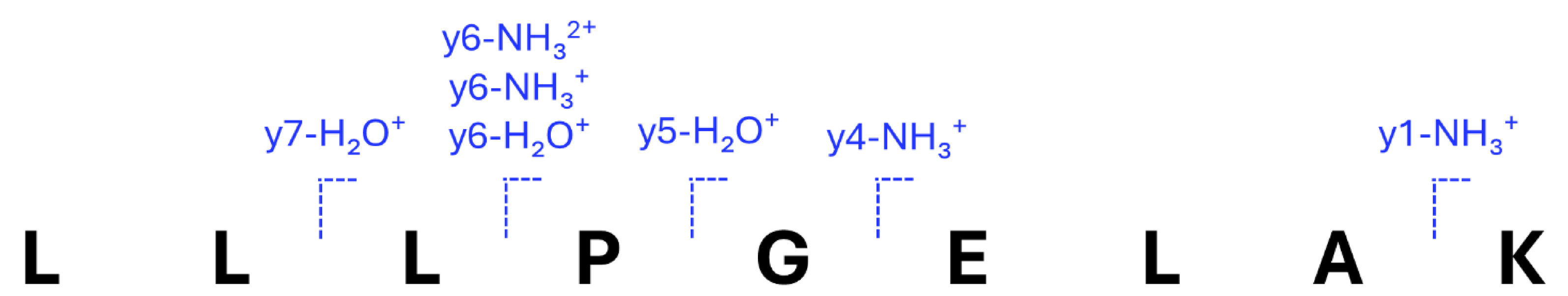

PVLIENSISI - ALOX12B

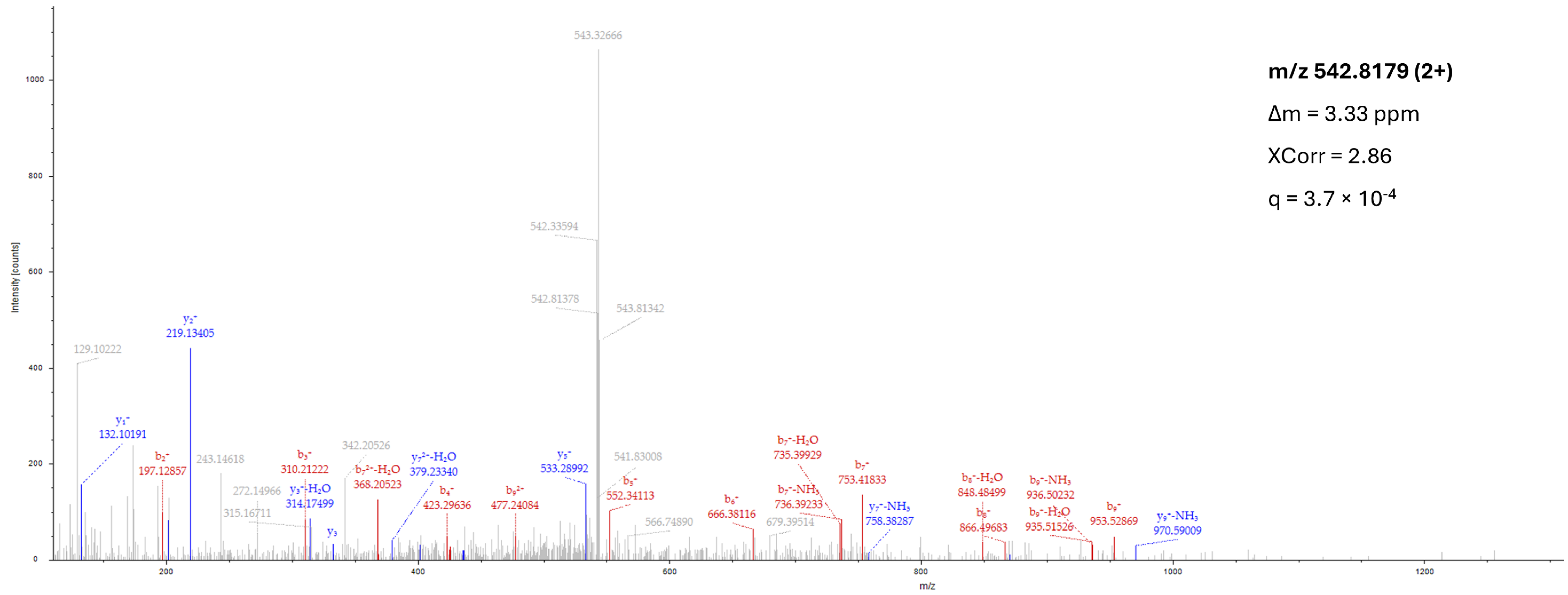

m/z 542.8179 (2+)

Δm = 3.33 ppm

XCorr = 2.86

q = 3.7 × 10^{-4}

Fragment ions

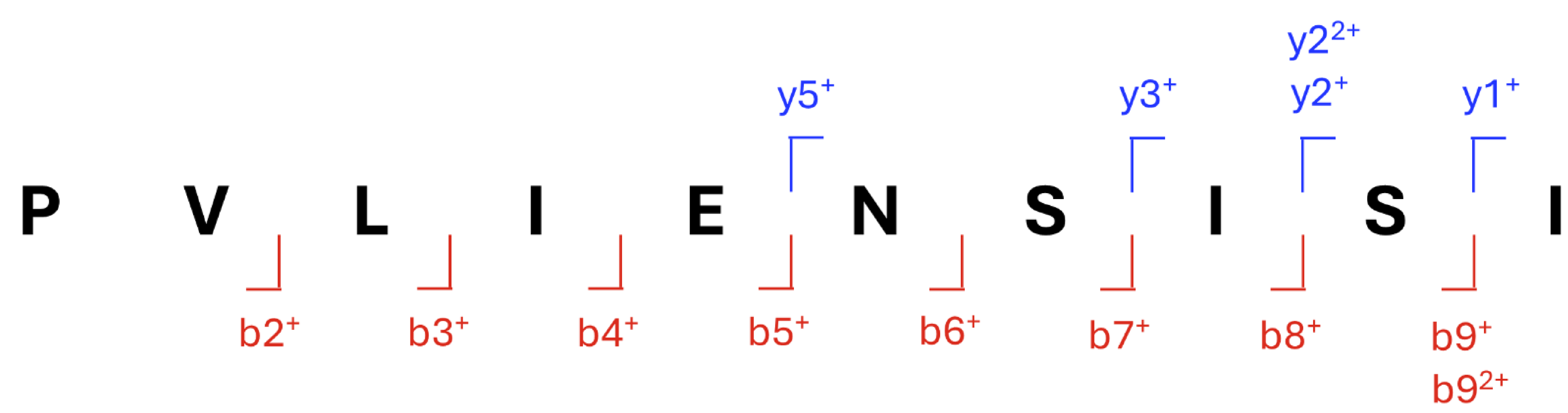

Neutral-loss fragment ions

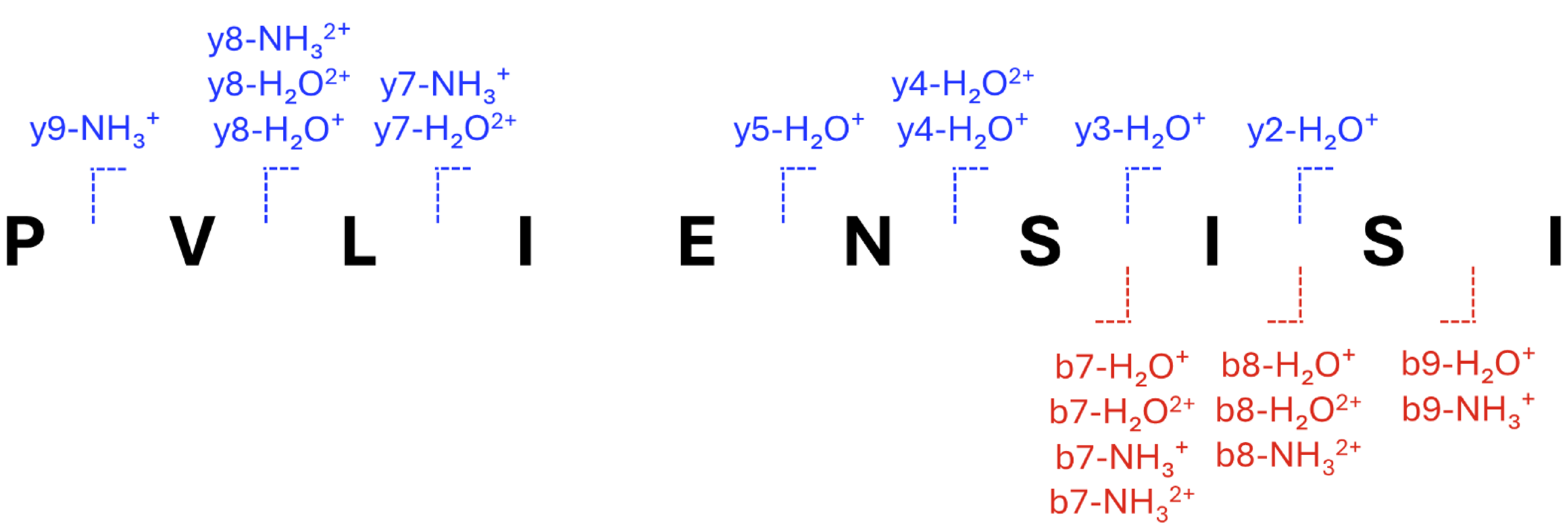

ILNIFGVIK - TFRC

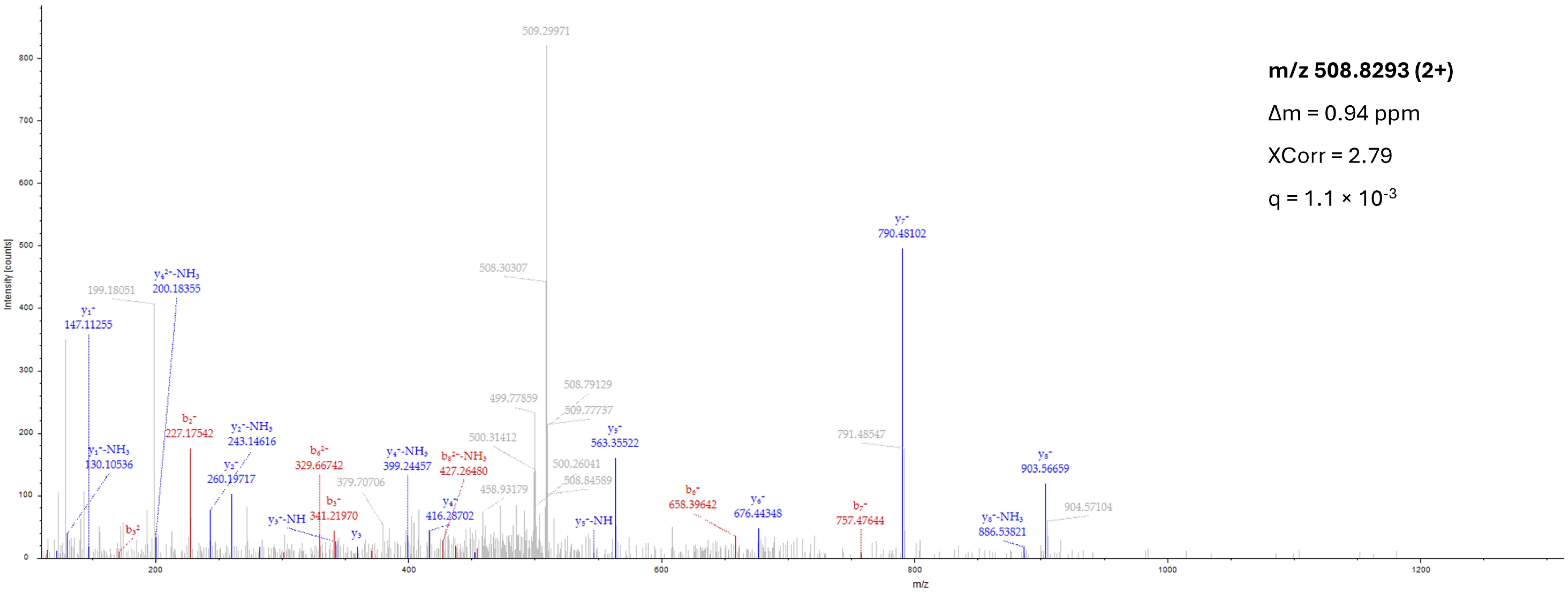

m/z 508.8293 (2+)

Δm = 0.94 ppm

XCorr = 2.79

q = 1.1 × 10^{-3}

Fragment ions

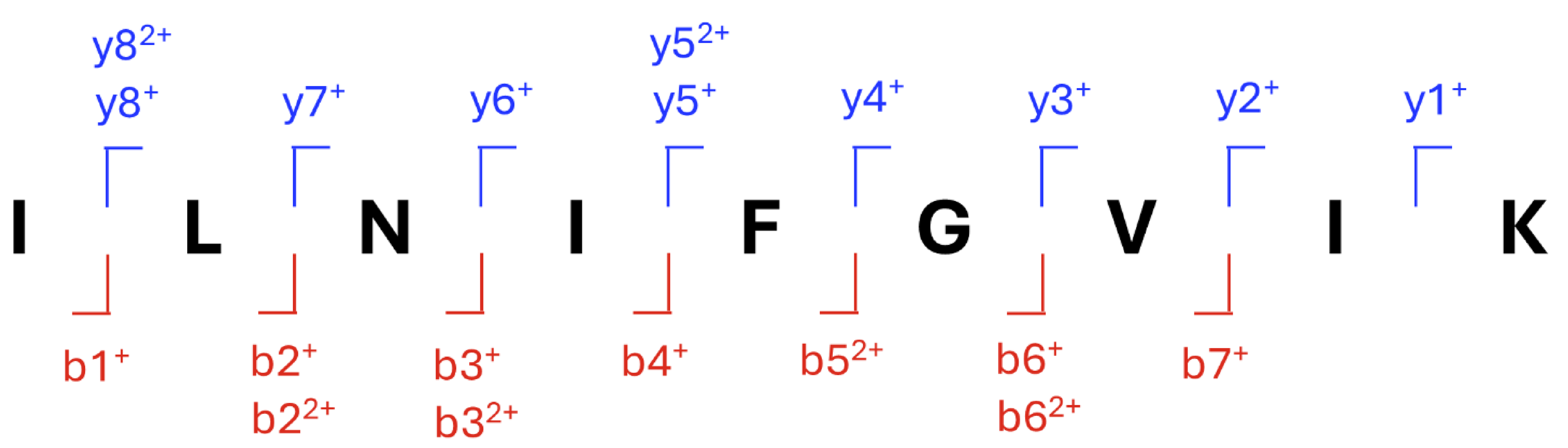

Neutral-loss fragment ions

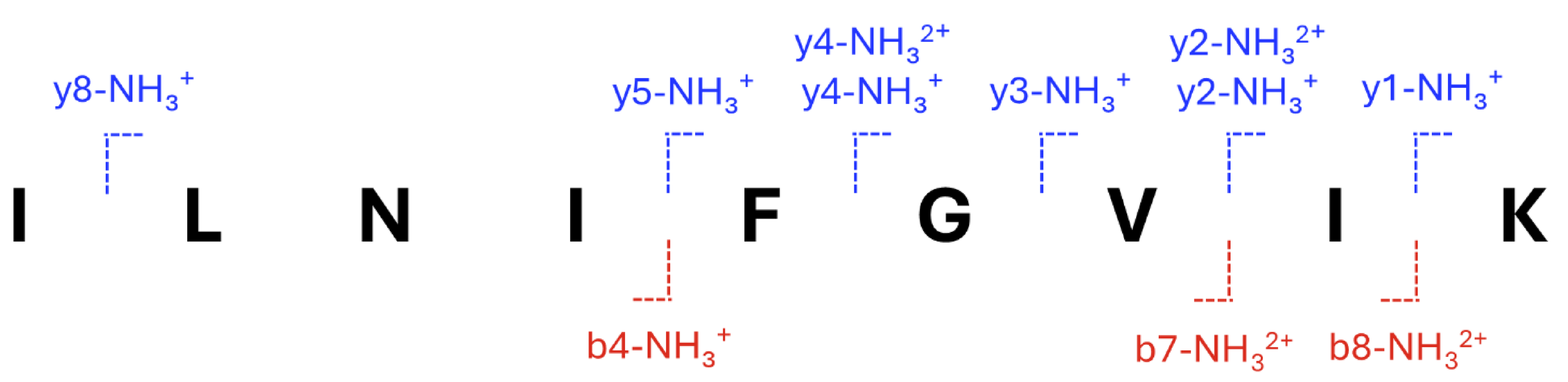

ITEEIYKTGL - ARG1

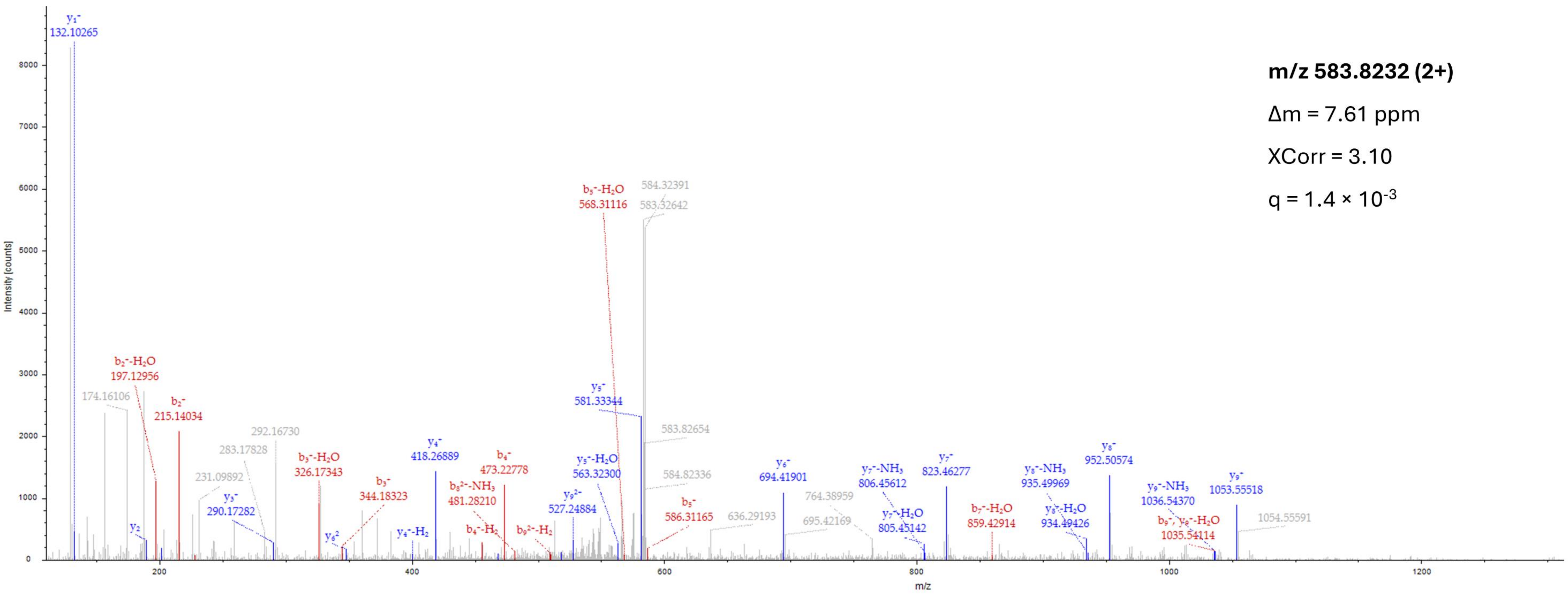

m/z 583.8232 (2+)

Δm = 7.61 ppm

XCorr = 3.10

q = 1.4 × 10^{-3}

Fragment ions

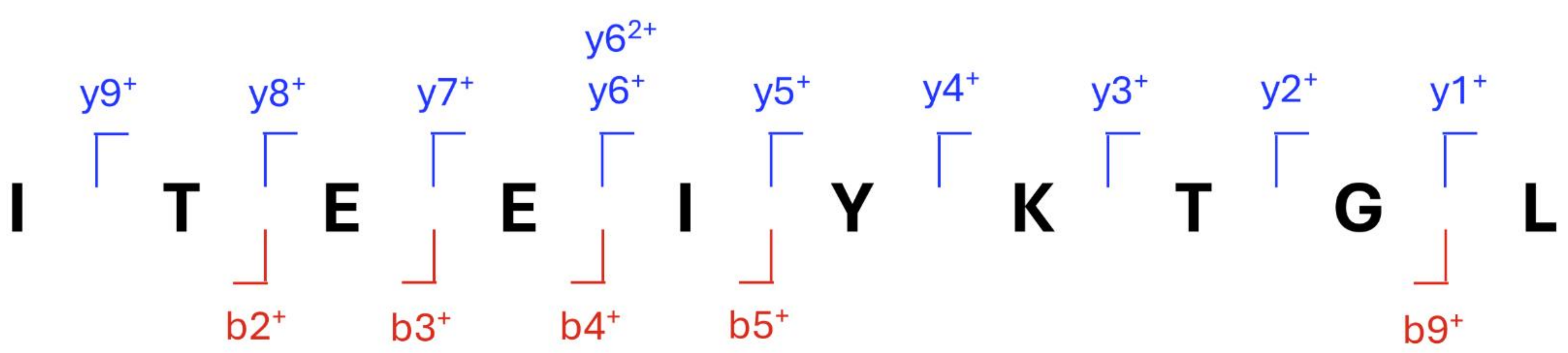

Neutral-loss fragment ions

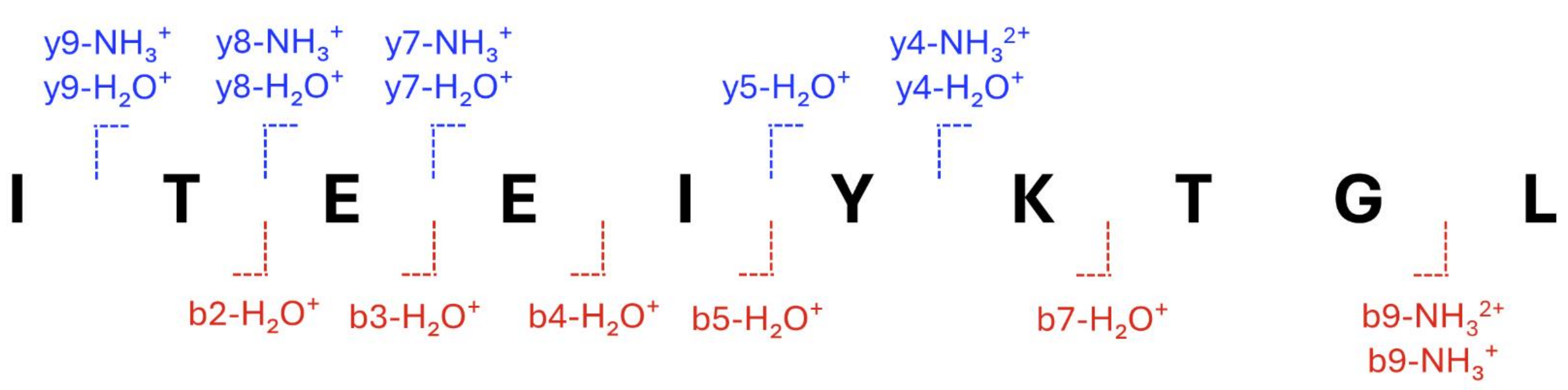

Supplement: Supplementary file 1 [file JEV2-15-e70389-s001.pdf]
