# Supplementary material for: Oncolytic Virus‐Engineered Extracellular Vesicles Rewire Glioblastoma Immunity
Source: J Extracell Vesicles. 2026 Oct 21;15(10):e70389. doi: 10.1002/jev2.70389 (PMC13687664; doi:10.1002/jev2.70389)
Supplement: Supplementary file 2 [file JEV2-15-e70389-s002.docx]

| **REAGENT OR RESOURCE** | **SOURCE** | **IDENTIFIER** |
| --- | --- | --- |
| Antibodies | | |
| BD Horizon™ BUV496 Mouse Anti-Human CD3 clone UCHT1 | Invitrogen | #612940,  ( RRID:AB_2870222) |
| Anti-human CD8a Monoclonal Antibody PerCP-Cyanine5.5 clone RPA-T8 | Invitrogen | #45-0088-42,  ( RRID:AB_1582255) |
| CD25 Monoclonal Antibody (BC96), Super Bright™ 600, eBioscience™ | Invitrogen | #63-0259-42,  ( RRID:AB_2637187) |
| Anti-human CD69 Monoclonal Antibody eFluor450 clone FN50 | Invitrogen | #48-0699-42,  ( RRID:AB_2574025) |
| CD279 (PD-1) Monoclonal Antibody (MIH4), PE, eBioscience™ | Invitrogen | #12-9969-42,  ( RRID:AB_10736473) |
| CD366 (TIM3) Monoclonal Antibody (F38-2E2), Super Bright™ 780, eBioscience™ | Invitrogen | #78-3109-42,  ( RRID:AB_2724091) |
| CD45 Monoclonal Antibody (HI30), Brilliant Ultra Violet™ 395, eBioscience™ | Invitrogen | #363-0459-42,  ( RRID:AB_2920949) |
| CD4 Monoclonal Antibody (SK3 (SK-3)), FITC, eBioscience™ | Invitrogen | #11-0047-42,  ( RRID:AB_1272074) |
| CD14 Monoclonal Antibody (61D3), Super Bright™ 645, eBioscience™ | Invitrogen | #64-0149-42,  ( RRID:AB_2662458) |
| Anti-human CD16 Monoclonal Antibody NovaFluorBlue 610-70S clone eBioCB16 (CB16) | Invitrogen | #H025T03B06,  ( RRID:AB_2910727) |
| HLA-DR Monoclonal Antibody (LN3), Alexa Fluor™ 700, eBioscience™ | Invitrogen | #56-9956-42,  ( RRID:AB_1907427) |
| CD56 (NCAM) Monoclonal Antibody (TULY56), Brilliant Ultra Violet™ 737, eBioscience™ | Invitrogen | #367-0566-42,  ( RRID:AB_2895975) |
| BD Horizon™ BUV661 Mouse Anti-Human CD11c Clone B-ly6 (RUO) | BD Horizon | #612967,  ( RRID:AB_2870241) |
| FOXP3 Monoclonal Antibody (PCH101), PE-Cyanine7, eBioscience™ | Invitrogen | #25-4776-42,  ( RRID:AB_10804638) |
| CD303a Monoclonal Antibody (201A), APC-eFluor™ 780, eBioscience™ | Invitrogen | #47-9818-42,  ( RRID:AB_2784717) |
| CD83 Monoclonal Antibody (HB15e), Super Bright™ 702, eBioscience™ | Invitrogen | #67-0839-42,  ( RRID:AB_2802455) |
| Human Panel Biomarkers | RareCyte™ | Q0156 |
| Human Panel Biomarkers | RareCyte™ | Q0141 |
| Clathrin heavy chain Rabbit pAb | ABclonal | #A12423, RRID:AB_2759265 |
| HSP70/HSPA1 Mouse mAb | ABclonal | #A1507, RRID:AB_2761952 |
| Calnexin Rabbit mAb | ABclonal | #A4846, AB_2863359 |
| CD63 (E1W3T) Rabbit mAb | Cell signaling | #52090, RRID:AB_2924771 |
| Annexin A2 (D11G2) Rabbit mAb | Cell signaling | #8235, RRID:AB_11129437 |
| CD81 Antibody (B-11) | Santa Cruz Biotechnology | #sc-166029, RRID:AB_2275892 |
| CD54/ICAM-1 Antibody | Cell signaling | #4915, RRID:AB_2280018 |
| B7-H3 (D9M2L) XP® Rabbit mAb | Cell signaling | #14058, RRID:AB_2750877 |
| CD44 (E7K2Y) XP® Rabbit mAb | Cell signaling | #37259, RRID:AB_2750879 |
| WT1 (D8I7F) XP® Rabbit mAb | Cell signaling | #83535, RRID:AB_2800020 |
| PD-L1 (E1L3N®) XP® Rabbit mAb | Cell signaling | #13684, RRID:AB_2687655 |
| HMGA1 (D6A4) XP® Rabbit mAb | Cell signaling | #7777, RRID:AB_2797634 |
| CD44 (E7K2Y) XP ® Rabbit mAb | Cell signaling | #37259, RRID:AB_2750879 |
| HLA-ABC monoclonal antibody (W6/32) | eBioscience™ | # 14-9983-82  RRID:AB_468661 |
| Monoclonal Anti-HLA Class I Antigen antibody, (W6/32) | Merck | # H1650  RRID:AB_260021 |
| MHC Class I Mouse Monoclonal Antibody (EMR8-5) | Cell signaling | #88274 RRID:AB_3714874 |
| FoxP3 (D6O8R) Rabbit mAb | Cell signaling | #12653, RRID:AB_2797979 |
| Cleaved Caspase-3 (5A1E) Rabbit mAb | Cell signaling | #94530, RRID:AB_3076239 |
| Ki-67 (D2H10) Rabbit mAb (IHC Specific) | Cell signaling | #9027, RRID:AB_2636984 |
| PD-1 (Intracellular Domain) (D4W2J) XP® Rabbit mAb | Cell signaling | #63815, RRID:AB_3675993 |
| TIGIT (E5Y1W) XP® Rabbit mAb | Cell signaling | #99567, RRID:AB_2922806 |
| Goat anti-Rabbit IgG (H+L) Cross-Adsorbed Secondary Antibody, Alexa Fluor™ 555 | ThermoFisher | #A-21428, RRID:AB_2535849 |
| Anti-Granzyme B antibody [EPR8260] | Abcam | #ab134933, RRID:AB_2889221 |
| Anti-CD8 alpha antibody [EPR21769] | Abcam | #ab217344, RRID:AB_2890649 |
| Bacterial and viral strains | | |
| oHSV-1 vector rQNestin34.5v1 | Brigham and Women’s Hospital | - DOI: 10.1007/978-1-4939-0428-0_8 |
| *E. coli* DH5α competent cells | Thermo Fisher | - EC0112 |
| Biological samples | | |
| Buffy coats obtained from healthy donors | Regional Blood Center, Warsaw | Approval number WAW/111/2021 |
| Chemicals, peptides, and recombinant proteins | | |
| Pierce™ Bovine Serum Albumin Standard Ampules, 2 mg/mL | ThermoFisher Scientific | 23209 |
| UltraPure™ DNase/RNase-Free Distilled Water | Invitrogen | 10977-035 |
| GlycoBlue™ Coprecipitant (15 mg/mL) | ThermoFisher | AM9516 |
| Neurobasal™ Medium | Gibco | 21103049 |
| DMEM, High glucose | Gibco | 11965092 |
| B-27™ Supplement (50X), minus vitamin A | Gibco | 12587010 |
| MycoBlue Mycoplasma Detector | Vazyme Biotech | 50 rx |
| Precision Plus Protein Dual Color Standard | Bio-Rad | 1610374 |
| Mini-Protean TGX Stain-Free Precast Gels | Bio-Rad | 4568094 |
| 4-20% Criterion™ TGX Stain-Free™ Protein Gel, 12+2 well, 45 µl | Bio-Rad | 5678093 |
| 2-Methylbutane, 99+%, extra pure, Thermo Scientific Chemicals - 1L | Thermo Scientific Chemicals | 126470025 |
| PolyFreeze Tissue Freezing Medium | Merck | SHH0026-6X120ML |
| Vidisic moisturizing gel for dry eyes, 10 g | Inpharm | N/A |
| Human Heat Stable bFGF Recombinant Protein 100ug | Gibco | # PHG0369 |
| Human EGF Recombinant Protein 100ug | Gibco | PHG0311 |
| Penicillin-Streptomycin (10,000 U/mL) | Gibco | 15140122 |
| GlutaMAX (100x) | Gibco | 35050-061 |
| Bambanker™ - freezing medium 120ml | GC LYMPHOTEC INC. | BB01 |
| StemPro Accutase 100ml | Gibco | A11105-01 |
| GAPDH loading control | Thermo Scientific™ | MA5-15738 |
| Halt™ Protease and Phosphatase Inhibitor Cocktail (100X) | ThermoFisher Scientific | 78444 |
| Diethyl pyrocarbonate | Merck | D5758-25ML |
| D-luciferin 1g | Syd labs | MB000102-R70170 |
| Image-iT FX Signal Enhancer (blocking for IF) | Thermo Fisher | Cat#I36933 |
| Lymphoprep™ 500ml | StemCell | 7851 |
| eBioscience™ 1X RBC Lysis Buffer | ThermoFisher Scientific | 00-4333-57 |
| CellBlox™ Plus Blocking Buffer | ThermoFisher Scientific | C001T03F01 |
| Invitrogen™ TRI Reagent™ Solution | Invitrogen | AM9738 |
| Clarity Max Western ECL Substrate, 100 ml | Bio-Rad | 1705062 |
| PowerUp SYBR Green master mix | Thermo Fisher | A25742 |
| Super Bright Complete Staining Buffer, eBioscience™ | ThermoFisher Scientific | SB-4401-42 |
| Ampicillin | Thermo Scientific Chemicals | J60977.06 |
| Puromycin | Gibco | A1113803 |
| LB Broth with agar | Sigma | L3147-250G |
| DNase I, RNase-free (1 U/μL) | ThermoFisher Scientific | EN0521 |
| UltraComp eBeads™ Compensation Beads | Invitrogen | 01-2222-41 |
| LIVE/DEAD™ Fixable Blue Dead Cell Stain Kit, for UV excitation | Invitrogen | L34962 |
| Fetal Bovine Serum, exosome-depleted 50 mL | Gibco | A5256801 |
| TrypLE™ Express Enzyme (1X), phenol red | Gibco | 12605010 |
| RPMI 1640 Medium, GlutaMAX™ Supplement | Gibco | 61870010 |
| Protein A-Sepharose 4B Conjugate | ThermoFisher Scientific | # 10-1041 |
| n-Octyl-β-D-glucopyranoside | Thermo Scientific Chemicals | # J67390.03 |
| Sodium Deoxycholate Detergent | ThermoFisher Scientific | # 89904 |
| Halt™ Protease and Phosphatase Inhibitor Cocktail (100×) | ThermoFisher Scientific | # 78444 |
| EDTA (0.5 M), pH 8.0, RNase-free | ThermoFisher Scientific | # AM9260G |
| Iodoacetamide | Merck | # I1149 |
| Phenylmethanesulfonyl fluoride (PMSF) | Merck | #93482-50ML-F |
| Critical commercial assays | | |
| Visium Spatial Tissue Optimization Kit | 10X Genomics | Cat # PN-1000193 |
| Visium Accessory Kit | 10X Genomics | Cat #PN-1000194 |
| Dual Index Kit TT Set A | 10X Genomics | Cat #PN-1000215 |
| Visium Spatial Gene Expression Slide and Reagent Kit | 10X Genomics | Cat # PN-1000184 |
| FAST SYBR qPCR Master Mix | KAPA Biosystems | Cat # KK4600 |
| BCA Protein Assay Kit | Thermo Fisher | Cat#23227 |
| High-capacity RNA-to-cDNA kit | ThermoFisher | 4368814 |
| Plasmid Midi AX Kit | A&A Biotechnology | 092-10 |
| ScaleBio Single-cell Kit | ScaleBio | 2020002 |
| RNeasy Mini Kit | Qiagen | 74104 |
| ScaleBio Sample Fixation Kit | ScaleBio | 2020001 |
| EZ-Retriever Automated Antigen Retrieval | BioGenex | MW015-IR |
| Visium Spatial Gene Expression Kit | 10X Genomics | Cat#1000183, RRID: SCR_023571 |
| Agilent High Sensitivity DNA Kit (Bioanalyzer) | Agilent | 5067-4626 |
| QuantiFluor dsDNA System | Promega | E2670 |
| DuoSet Ancillary Reagent Kit 2 | R&D Systems | DY008B |
| NucleoSpin Tissue Kit | MACHEREY-NAGEL | 740952.10 |
| HSV 1&2 R-Gene assay | bioMérieux | 69-004 |
| SensiFAST Probe No-ROX Kit | Bioline/Meridian Bioscience | BIO-86005 |
| Human IFN-γ DuoSet ELISA | R&D Systems | DY285B-05 |
| LEGENDplex HU essential Immune Response Panel (13-plex) w/VbP | BioLegend | 740930 |
| Deposited data | | |
| scRNA-seq | EGA | EGAS50000001124 |
| MS | PRIDE | PXD054707 |
| Spatial transcriptomics Visium HD™ | GEO | GSE308834 |
| Experimental models: Cell lines | | |
| Patient-derived glioblastoma stem-like cells (GSCs) | Brigham and Women’s Hospital | DOI: 10.1158/0008-5472.CAN-15-3432  DOI: 10.1126/sciadv.aar2766 |
| Vero Cells | Brigham and Women’s Hospital ATCC | Cat#CCL-81, RRID:CVCL_0059 |
| HEK 293T | ATCC | Cat# CRL-3216, RRID:CVCL_0063 |
| Experimental models: Organisms/strains | | |
| NSG mice (NOD.Cg-Prkdc^scid Il2rg^tm1Wjl/SzJ) | Jackson Laboratory | Cat#005557, RRID: IMSR_JAX:005557 |
| Oligonucleotides | | |
| METfor | Genomed | TAGCCA ACCGAG AGACAA GC |
| METrev | Genomed | TGTGCT CCCACC ACTAAT AAAAG |
| SOX2for | Genomed | GAGAAGTTTGAGCCCCAGG |
| SOX2rev | Genomed | AGAGGCAAACTGGAATCAGG |
| CD133for | Genomed | ACTCCCATAAAGCTGGACCC |
| CD133rev | Genomed | TCAATTTTGGATTCATATGCCTT |
| CD44for | Genomed | AAGGTGGAGCAAACACAACC |
| CD44 rev | Genomed | CTTCTGCCCACACCTTCTTC |
| NESfor | Invitrogen | TCTGCAAACCCATCGGACTC |
| NESrev | Invitrogen | AGGCACCTTTTCTTCCTGGG |
| ICP4for | Genomed | CGACACGGATCCACGACCC |
| ICP4rev | Genomed | GATCCCCCTCCCGCGCTTCGTCCG |
| ICP8for | Genomed | AAGCTGGTTGCGTTGGAG |
| ICP8rev | Genomed | TTTCTGCTGAAGCAGTTCCA |
| ICP0for | Genomed | CACCACGGACGAGGATGAC |
| ICP0rev | Genomed | GGCGGGCGGTACGT |
| VP5for | Genomed | GACCGACGGGTGCGTTATT |
| VP5rev | Genomed | GAAGGAGTCGCCATTTAGCC |
| Recombinant DNA | | |
| Plasmid pCMV-Fluc | Addgene | Cat# 170575, RRID: Addgene_170575 |
| Plasmid psPAX2 | Addgene | RRID: Addgene_12260 |
| Plasmid pMD2.G | Addgene | RRID: Addgene_12259 |
| Software and algorithms | | |
| BioRender | BioRender | https://www.biorender.com/, (RRID: SCR_018361) |
| Spectronaut Software | Biognosys AG | v18.3 |
| Cytobank | Beckman Coulter | [https://www.cytobank.org](https://www.cytobank.org/), (RRID: SCR_014043) |
| SpectroFlo 3.2.1 | Cytek Biosciences | (RRID: SCR_025494) |
| Space Ranger v3.0 | 10X Genomics | https://www.10xgenomics.com/support/software/space-ranger/3.0,  (RRID: SCR_025848) |
| GeneSpring GX v12.1 software | Agilent Technologies Santa Clara | (RRID: SCR_010972) |
| Agilent Feature Extraction software (version 11.0.1.1) | Agilent Technologies Santa Clara | (RRID: SCR_014963) |
| Proteomaps | Proteomaps | https://proteomaps.net/ |
| Morpheus | Broad Institute | https://software.broadinstitute.org/morpheus,  (RRID: SCR_017386) |
| Protter | ETH Zürich | http://wlab.ethz.ch/protter/start/ |
| R v4.3.0 (R Projects for Statistical Computing) | R Project | [https://www.r-project.org](https://www.r-project.org/) ,(RRID: SCR_001905) |
| GraphPad Prism v9 | GraphPad Software | [https://www.graphpad.com](https://www.graphpad.com/), (RRID: SCR_002798) |
| Seurat v5.0.2 | Satija Lab | <https://satijalab.org/seurat/>, (RRID: SCR_016341) |
| MSigDB Gene Ontology BP/MF collections | Broad Institute | https://www.gsea-msigdb.org/gsea/msigdb, (RRID:SCR_016863) |
| presto R package | GitHub - immunogenomics/presto | https://github.com/immunogenomics/presto |
| fgsea R package | Bioconductor | https://bioconductor.org/packages/fgsea/,  (RRID: SCR_020938) |
| flowStats | Bioconductor | https://www.bioconductor.org/packages/release/bioc/html/flowStats.html,  (RRID: SCR_000399) |
| CATALYST R Library (version 1.26.1) | Bioconductor | https://bioconductor.org/packages/release/bioc/html/CATALYST.html,  (RRID: SCR_017127) |
| sva package (v3.58.0) | Bioconductor | http://www.bioconductor.org/packages/release/bioc/html/sva.html  (RRID: SCR_012836) |
| Lme4 | R package | https://cran.r-project.org/web/packages/lme4/index.html  (RRID: SCR_015654) |
| emmeans | R package | https://CRAN.R-project.org/package=emmeans  (RRID: SCR_018734) |
| diffcyt Software R package (version 1.23.1) | Bioconductor | https://bioconductor.org/packages/release/bioc/html/diffcyt.html,  (RRID: SCR_023006) |
| FlowSOM (version 2.1.0) | FlowSOM | https://github.com/SofieVG/FlowSOM, (RRID: SCR_016899) |
| UniProt | UniProt | https://www.uniprot.org/, (RRID:SCR_002380) |
| ggplot2 v3.3.6 | R Package | https://cran.r-project.org/web/packages/ggplot2/index.html  , (RRID: SCR_014601) |
| enrichplot v1.18.4 | YuLab-SMU, GitHub repository | https://github.com/YuLab-SMU/enrichplot,  (RRID: SCR_026996) |
| DESeq2 (v. 1.38.3) | Bioconductor | https://bioconductor.org/packages/release/bioc/html/DESeq2.html,  (RRID: SCR_015687) |
| clusterProfiler | Bioconductor | https://bioconductor.org/packages/release/bioc/html/clusterProfiler.html,  (RRID: SCR_016884) |
| ReactomePA | Bioconductor | https://bioconductor.org/packages/release/bioc/html/ReactomePA.html,  (RRID: SCR_019316) |
| scrna-recom | WXlab-NJMU, GitHub repository | https://github.com/WXlab-NJMU/scrna-recom?utm_source=chatgpt.com |
| FlowJo v10.10 | BD Bioscience | https://www.flowjo.com/, (RRID: SCR_008520) |
| LEGENDplex™ Analysis Software | BioLegend | Cat# 02-0022-00 |
| ImageJ | ImageJ | RRID:SCR_003070 |
| SRplot | NewCore Biotech | <https://bioinformatics.com.cn/srplot> PMID: 37943830 RRID:SCR_025904 |
| ZEISS ZEN Microscopy Software | Carl Zeiss | RRID:SCR_013672 |
| Zeiss: LSM 780 Confocal Laser Scanning Microscope | Carl Zeiss | RRID:SCR_020922 |
| Other | | |
| NanoSight Instrument | Malvern Panalytical | NS300 |
| RareCyte™ Orion Imaging System | RareCyte™ Inc. | RRID: SCR_026855 |
| NanoDrop 2000 Spectrophotometer | Thermo Scientific | RRID: SCR_018042 |
| Quantus Fluorometer | Promega | RRID: SCR_026279 |
| Agilent 2100 Bioanalyzer Instrument | Agilent | RRID: SCR_018043 |
| Illumina NovaSeq 6000 Sequencing System | Illumina | RRID: SCR_016387 |
| MicroAmp™ EnduraPlate™ Optical 96-Well Clear Reaction Plates | Applied Biosystems | A36924 |
| Corning® Falcon® Round Bottom Test Tubes | Merck | CLS352054 |
| Western Blotting Filter Papers, 0.83 mm thick, 8 x 10.5 cm | ThermoFisher Scientific | 88600 |
| Non-absorbable silk surgical sutures, size 5/0 | Peters surgical | 25143 |
| Precision dosing syringes, Injekt®-F Solo | B. Brown | 720-2561 |
| Sterican® disposable needles | B. Brown | 612-0158 |
| Cryomold® Biopsy 10 x 10 x 5 mm | Tissue-tek | AGG4581 |
| Cryomold® Intermediate 15 x 15 x 5 mm | Tissue-tek | AGG4582 |
| Eppendorf® DNA LoBind tubes 1.5ml | Merck | EP0030108051-250EA |
| Eppendorf® DNA LoBind Tubes (CONTAINING PRODUCT EP0030108035) | Merck | EP0030108310-200EA |
| Dumont #7 bent forceps, biological tips, Dumoxel, 11.5 cm | FST | 11272-30 |
| Dumont #7 fine bent forceps, biological tips, Inox, 11 cm | FST | 11274-20 |
| SuperFrost® Plus microscope slides | BioNovo | B-1211 |
| ArgoFluor Mounting Medium | RareCyte™ | Cat#AR101 |
| Peel-A-Way™ embedding molds | Sigma-Aldrich | E6032 |
| Tissue-Tek® O.C.T. Compound | Tissue-tek | 4583 |
| 10x Tris/Glycine/SDS | Bio-rad | #1610732 |
| T75 Nunclon Sphera EasYFlask | ThermoFisher Scientific | 174952 |
| Nunclon Sphera 24-Well Plate, case of 7 | ThermoFisher Scientific | 174930 |
| Nunclon Sphera 12-Well Plate, case of 7 | ThermoFisher Scientific | 174931 |
| Nunclon™ Sphera™ Dishes 6-Well Plate | ThermoFisher Scientific | 174932 |
| Vetflurane 1000mg/g | Virbac | Vnr 13 73 17 |
| Precision Dosing Syringes, Injekt®-F Solo | B. Brown | 720-2561 |
| Ketamine | Biowet Puławy | N/A |
| Domitor 1 mg/ml Solution for Injection 10ml | VETOQUINOL | N/A |
| Gilbert NaCl 0.9%, saline solution, 100 ampoules of 5 ml | LABORATOIRES GILBERT | N/A |
| Alzane | Syva | N/A |
| ENROCIN® 5% inj. | Vet Agro | N/A |
| Tolfine Inj.- 50 ml | VETOQUINOL | N/A |
| Revertor 5 mg/ml Solution for Injection for Cats and Dogs | Chanelle Pharma | N/A |
| DICOTUBER 1ml tuberculin syringes (ZARYS) with 0.45 x 13 needle (100 pcs.) | ZARYS | N/A |
| Disposable needles, Sterican® | B. Brown | 612-0158 |
| Heating mat with thermostat for terrarium 28x65 35W | ADELID | MG-35W |
| NON-ABSORBABLE SILK THREADS, SUTURES - SILK size 5/0 | Peters surgical | 25143 |
| SuperFrost® Plus Basic Microscope Slides | BioNovo | B-1211 |
| Zeiss Axio Vert.A1 FL | Zeiss | Model 431030-9040-000 |
| IVIS Spectrum | Revvity | 124262 |
| ChemiDoc | Biorad | 12003153 |
| BD LSRFortessa™ | BD Biosciences | 656385 |
| Cytek Aurora spectral flow cytometer | Cytek Biosciences | Aurora |
