# Supplementary material for: Oncolytic Virus‐Engineered Extracellular Vesicles Rewire Glioblastoma Immunity
Source: J Extracell Vesicles. 2026 Oct 21;15(10):e70389. doi: 10.1002/jev2.70389 (PMC13687664; doi:10.1002/jev2.70389)
Supplement: Supplementary file 3 [file JEV2-15-e70389-s003.docx]

**Figure S1. Profiling of systemic immune cells in the NSG model**

(A) Representative Western Blot images of markers with indicated antibodies in GSCs and the EV fraction.

(B-C) Scatter plots of NTA of modal size (B) and concentration (C) of EVs and EVOVs (n=9).

(D) Distribution plot of NTA of mean size and concentration of EVs and EVOVs (n=3).

(E) Gating strategy used to identify CD45⁺ cell clusters by MPFC.

(F) Gating strategy used to identify CD45⁺ cell clusters by MPFC.

(G) UMAP projections overlaid by acquisition batch (Day 1-5) for CD3⁺ (left) and CD3⁻ (right) cell clusters of PBMC (n=8) by MPFC.

(H) UMAP projections of CD3⁺ (left) and CD3⁻ (right) cell clusters of mock-primed (PBS) PBMC (n=8) by MPFC.

**Figure S2. Characterization of CD3^⁺^ subpopulations from primed PBMCs.**

(A) Bar plots of CD3⁺ cell clusters of mock- (PBS), EV-, or EVOV-primed (n=6) PBMC (n=8) by MPFC, mixed-effects model with Tukey correction, *p≤0.05, **p≤0.01, ***p≤0.001.

(B) Scatter plots of cytokine profiles in supernatants of PBMCs (n=3) primed with EV or EVOV (n=6) by MPFC, paired t-test *p≤0.05.

**Figure S3. Characterization of CD3^-^ subpopulations from primed PBMCs.**

(A) Bar plots of CD3^-^ cell clusters of mock- (PBS), EV-, or EVOV-primed (n=6) PBMC (n=8) by MPFC, mixed-effects model with Tukey correction, *p≤0.05, **p≤0.01, ***p≤0.001.

**Fig. S4. PBMC viability and immune composition after EV/EVOV dose escalation**

**(A) Bar plot of CD45⁺ PBMCs viability by MPFC after 72 h treatment with EV, EVOV, high-dose EV (EV_H), or high-dose EVOV (EVOV_H) (n=6). Mixed-effects model with Tukey correction.**

**(B) Heatmaps of differential abundance of CD3⁺ cell clusters of PBMC (n=4) primed with EV, EVOV, EV_H, or EVOV_H (n=6) by MPFC, mixed-effects model with Tukey correction.**

**(C) Stacked bar plot of the composition of CD3⁺ immune cell clusters among PBMCs (n=4) primed with PBS, EV, EVOV, EV_H, or EVOV_H and analyzed by MPFC. Proportions are shown as percentages within each condition.**

**(D) Heatmaps of differential abundance of CD3^-^ cell clusters of PBMC (n=4) primed with EV, EVOV, EV_H, or EVOV_H (n=6) by MPFC, mixed-effects model with Tukey correction.**

**(E) Stacked bar plot of the composition of CD3^-^ immune cell clusters among PBMCs (n=4) primed with PBS, EV, EVOV, EV_H, or EVOV_H and analyzed by MPFC. Proportions are shown as percentages within each condition.**

**Figure S5. Characterization of CD3⁺ subpopulations from organoids.**

(A) Bar plots of CD3^+^ cell clusters (n=4) derived from organoids with mock (PBS), EV, or EVOV-primed PBMCs (n=4) by MPFC, mixed-effects model with Tukey correction, *p≤0.05, **p≤0.01, ***p≤0.001.

(B) Representative immunofluorescence images with indicated antibodies of organoids with PBMC primed with EV or EVOV by MPI, scale bar=50μm.

**Figure S6. Characterization of CD3^-^ subpopulations from organoids.**

(A) Bar plots of CD3^-^ cell clusters (n=4) derived from organoids with mock(PBS), EV, or EVOV-primed PBMCs (n=4) by MPFC, mixed-effects model with Tukey correction, *p≤0.05, **p≤0.01, ***p≤0.001.

(B) Representative immunofluorescence images with indicated antibodies of organoids with PBMC primed with EV or EVOV by MPI, scale bar=50μm.

**Figure S7. Representative MPI characterization of GSC–PBMC organoids.**

**(A-B) Representative images with indicated antibodies of organoids with PBMC primed with EVOV by MPI. Scale bar = 200 μm.**

**Fig. S8. Single-cell analysis across organoids**

**(A) Dot plot of replicate-level expression of selected IFN/MHC-I-related genes across GSCs in organoids with PBMC primed with EV or EVOV (n=3) by scRNA-seq. Dot size indicates the percentage of cells expressing each gene, and color indicates scaled average expression.**

(B) Volcano plots of differentially expressed genes in basophils and macrophages/MO from organoids with PBMC primed with EV or EVOV (n=3) by scRNA-seq, aggregated expression, DESeq2, Wald test, FDR≤0.05.

(C) Bar plots of significantly enriched downregulated pathways in selected cell subpopulations from organoids with PBMC primed with EV or EVOV (n=3) by scRNA-seq, aggregated expression, hypergeometric test, FDR≤0.05.

**(D) Bar plot of Cohen’s d effect-size distributions for EVOV–EV transcriptional differences of selected IFN/MHC-I-related genes across GSC subpopulations in organoids with PBMC primed with EV or EVOV (n=3) by scRNA-seq. Effect sizes provide a standardized estimate of the magnitude and directionality of EVOV-associated changes across annotated tumor states.**

**(E) Heatmap of IFN/MHC-I-related gene expression across GSC subpopulations from organoids with PBMC primed with EV or EVOV (n=3) by scRNA-seq, shown as scaled expression.**

(F) Dot plot of IFN-response gene expression across GSC subpopulations from organoids with PBMC primed with EV or EVOV (n=3). Dot size indicates the percentage of cells expressing each gene, and color indicates scaled average expression.

(G) Dot plot of MHC-I antigen-processing and antigen-presentation gene expression across GSC subpopulations from organoids with PBMC primed with EV or EVOV (n=3). Dot size indicates the percentage of cells expressing each gene, and color indicates scaled average expression.

**Figure S9. Profiling of systemic immune cells in the NSG model**

(A) Bar plots of CD3⁺ cell clusters of PBMC from blood of mice treated with EV (n=7) or EVOV (n=5) by MPFC, mixed-effects model with Tukey correction, *p≤0.05, **p≤0.01, ***p≤0.001, ****p≤0.0001.

**Fig. S10. Spatial distribution and functional profiling of immune-cell compartments in the NSG model**

(A) Representative images of T cell subpopulation clusters in brain tissue from mice treated with EV or EVOV by VisiumHD™, scale bar=500μm.

(B) Representative images of CD8A and CD4 expression in brain tissue from mice treated with EV or EVOV by VisiumHD™, scale bar=100μm.

(C) Representative images of plasma cell subpopulation clusters in brain tissue from mice treated with EV or EVOV by VisiumHD™, scale bar=500μm.

(D) Representative spatial maps of the cytotoxicity score in brain tissue from mice treated with EV or EVOV by VisiumHD™, scale bar=500μm.

(E) Representative spatial maps of the proliferation score in brain tissue from mice treated with EV or EVOV by VisiumHD™, scale bar=500μm.

**(F) Stacked bar plot showing the within-sample proportion of annotated T-cell bins assigned to periphery, border, or core regions in each VisiumHD™ section.**

(G) Violin plots of cytotoxicity scores in periphery T cells, border T cells, and core T cells in brain tissue from mice treated with EV or EVOV by VisiumHD™.

(H) Stacked bar plots showing the proportion of exhausted and non-exhausted T-cell bins within periphery T cells, border T cells, and core T cells in EV and EVOV conditions.

**(I) EVOV–EV percentage-point contrast in within-sample proportions of annotated T-cell bins assigned to core, border, or periphery regions. Because the analyzed VisiumHD sections are not anatomically identical, absolute T-cell bin or cell counts were not compared between samples.**

(J) Bar plots of significantly enriched downregulated pathways (fgsea, padj≤0.1) among genes ranked by differential expression statistics (Presto Wilcoxon/AUC ranking) in annotated immune-cell subpopulations from brain tissue by VisiumHD™ in EV and EVOV (n=2).

**Fig. S11. Spatial distribution and functional profiling of tumor-cell compartments in the NSG model**

**(A) Representative images of tumor cell subpopulation clusters in brain tissue from mice treated with EV or EVOV by VisiumHD™, scale bar=500μm.**

**(B) Bar plots of significantly enriched downregulated pathways (fgsea, padj≤0.1) among genes ranked by differential expression statistics (Presto Wilcoxon/AUC ranking) in annotated tumor-cell subpopulations from brain tissue by VisiumHD™ in EV and EVOV (n=2).**

**(C) Stacked bar plot of the distribution of annotated tumor-cell bins across MES1 Border, MES1 Border-ANG, MES1 Border-IFN, MES2 Core, and MES2 Core Hypoxic states in individual Visium HD sections from EV- and EVOV-treated mice (n = 2 per group). Proportions are shown as percentages within each sample.**

**(D) Representative spatial maps of leukocyte migration, type II IFN production, and hypoxia scores in brain tissue from mice treated with EV or EVOV by VisiumHD™, scale bar=500μm.**

**Figure S12. Spatial architecture and marker expression of tumor and T-cell populations in the intracranial GSCs NSG model**

**(A) Representative spatial maps of annotated tumor-cell states and T-cell localization in brain tumor sections from mice treated with EV or EVOV by VisiumHD™. Tumor cells were classified as MES1 border, MES1 border-ANG, MES1 border-IFN, MES2 core, or MES2 core-hypoxic, whereas T cells were assigned to the tumor periphery, border, or core; scale bar 100 μm.**

**(B) UMAP projections of *CD8A, CD4, FOXP3, MKI67, CASP3*, and *GZMB* expression in T-cell and tumor cell populations in brain tissue from mice, with corresponding linear expression plots comparing EV and EVOV groups by VisiumHD™ (n=2).**

**Figure S13. Immunofluorescence analysis of tumor proliferation, apoptosis, and immune-cell infiltration markers in the intracranial GSC NSG model**

**(A) Representative immunofluorescence images of brain tumor sections (left) from mice treated with EV (n=4) or EVOV (n=3) stained with the indicated antibody combinations. Scatter plots (right) show the corresponding quantification of single- and double-positive objects; data are presented as individual values with mean ± SEM; scale bar 20 μm.**

**Fig. S14. Quantitative robustness of VisiumHD™ spatial transcriptomic analyses**

**(A) Bar graph of standardized effect-size estimates calculated using Hedges’ g for selected spatial module scores across annotated T-cell, tumor-cell, and plasma-cell groups by VisiumHD™ in EV and EVOV (n=2).**

**(B) Scatter plot of pseudobulk replicate-reproducibility for tumor cells (top), plasma cells (middle), and T cells (bottom) by VisiumHD™ in EV and EVOV (n=2). Raw spatial UMI counts were aggregated into compartment-level pseudobulk profiles, log-normalized, and compared between biological replicates within groups.**

**Figure S15. Immunopeptidome profiling of EVs and EVOVs**

(A) Chromatograms of total ion current across EV and EVOV (n=6) by DIA-MS.

(B) Workflow overview for immunopeptide analysis.

(C) Heatmap and hierarchical clustering of peptides by DIA-MS in EV and EVOV (n=6).

(D) Scatter plot of MHC class I protein intensity by DIA-MS in EV and EVOV (n=6), paired t-test *p≤0.05.

(E) Volcano plot of EV and EVOV (n=6) immunopeptides abundance by DIA-MS, paired t-test *p≤0.05.

(F) Heatmap and hierarchical clustering of immunopeptides by DIA-MS in EV and EVOV (n=6).

(G) Bar plot of distribution of peptide modifications by DIA-MS in EV and EVOV (n=6).

**Fig. S16. Global proteomic remodeling of EVOV cargo**

**(A) Scatter plot of PCA proteomes by DIA-MS in EV and EVOV (n=6).**

**(B-C) Bar plots of the number of identified protein groups (D) and peptide intensities (E) by DIA-MS in EV and EVOV (n=6).**

**(D) Violin plot of coefficient variation for protein quantification by DIA-MS in EV and EVOV (n=6).**

**(E) Heatmap and hierarchical clustering of significantly deregulated proteins (*p≤0.05, FC >1.5) by DIA-MS in EV and EVOV (n=6).**

**(F) Heatmap of the score estimation by DIA-MS from significantly deregulated proteins (*p≤0.05, FC >1.5) in EV and EVOV (n=6).**

**(G) Proteomaps depicting functional categories of significantly upregulated (left) and downregulated (right) proteins by DIA-MS in EVOVs vs. EV (n=6).**

**(H) Scatter plot of the immune deconvolution scores of immune cells based on significantly deregulated proteins (paired t-test, *p≤0.05; **p≤0.01; ***p≤0.001) by DIA-MS in EV and EVOV (n=6).**

**Figure S17. Proteomic profiling of EVs and EVOVs**

(A) Representative Western Blot images with indicated antibodies of GSC markers.

(B) Heatmap of GSCs subtype classification based on the proteome by DIA-MS in EV (n=6).

**(C) Representative phase-contrast images of PBMCs primed with PBS, EV, EVOV, or oHSV-1 (left), and scatter plot of qPCR quantification of IFN-g transcript (right, n=2), scale bar = 20 μm.**

**(D) Bar plots showing the viability of mock- or oHSV-1-pretreated GSCs (left, n=3 in duplicates), and corresponding PBMCs (right, n=2 in triplicate) by MPFC after 72 h of organoid co-culture; paired t-test,** ****p≤0.01, ***p≤0.001.**

**(E) Bar plots showing the viability of oHSV-1-pretreated GSCs (left, n=3 in duplicates) and PBMC (right, n=2 in triplicate) primed with EV or EVOV by MPFC after 72 h of co-culture in an organoid; paired t-test, **p≤0.01, ***p≤0.001.**

**(F) Representative phase-contrast images of organoids formed with GSCs pretreated with mock or oHSV-1 and PBMCs primed with EV or EVOV (left) and bar plot of corresponding quantification of GSCs viability (middle, n=3 in duplicates), and PBMCs viability (right, n=2 in triplicate) by MPFC after 72 h of co-culture; scale bar 200 μm; paired t-test, *p≤0.05, **p≤0.01, ***p≤0.001.**

(G) Sankey plots of cell subtype clusters in individual organoids with PBMC primed with EV or EVOV (n=3) by scRNA-seq.

(H) Heatmap of the MHC-I signaling pathway network from organoids by scRNA-seq in EV (n=3).

(I) Scatter plots of IFN-γ concentration in supernatants of PBMCs (n=3) primed with EV (top), EVOV (bottom), and after MHC-I blockage (n=3) by ELISA.

(J) Scatter plot of qPCR quantification of IFN-γ in PBMCs primed with EV, EVOV, and after MHC-I blockage (n=6).

(K) Bar plots of CD3⁺ and CD3^-^ cell clusters of PBMC (n=2) primed with EV, EVOV, EV MHC-I blocked, or EVOV MHC-I blocked (n=3) by MPFC, paired t-test, *p≤0.05; **p≤0.01; ***p≤0.001; ****p≤0.0001.

(L) ELDA plots for GSCs (G157, G816) from co-cultures with PBMC primed with PBS, EV, or EVOV, ELDA likelihood ratio test (χ²), Stem cell frequencies 1/x with 95% confidence intervals.

(M) Representative pictures of GSC co-culture with PBMC primed with PBS, EV, EV-blockage, EVOV, EVOV-blockage, scale bar = 50μm.

**Fig. S18. MHC-I IP validation and EVOV-associated remodeling of MHC-I peptide cargo**

**(A) Representative immunofluorescence images of GSC–PBMC organoids primed with EV or EVOV, showing immune-cell infiltration and antigen-presentation-related marker expression by MPI, scale bar = 50 μm.**

**(B) Representative Western blot of MHC-I immunoprecipitation of EV/EVOV samples.**

**(C) Heatmap of top 6 MHC-I IP peptides showing EVOV-upregulated, EVOV-downregulated, and stable peptide groups in EV and EVOV (n=6).**

**(D) Bar plots of significantly enriched functional annotation of source genes corresponding to EVOV-upregulated (top, FC>2, p<0.05, n=95), unchanged MHC-I IP peptides (middle, n=38), and EVOV-downregulated (bottom, FC<2, p<0.05, n=70) in EV and EVOV (n=6).**

**(E) Dot plot of EV and EVOV MHC-I peptide source-gene (n=6) response scores in corresponding GSC–PBMC organoids primed with EV or EVOV (n=3) by scRNA-seq. Dot color and dot size indicate the mean of EVOV over EV module-score difference; dot size indicates the percentage of cells expressing each gene.**

**(F) Dot plot of MHC-I IP peptide source genes (n=6) across PBMC and GSC subpopulations from organoids with PBMC primed with EV or EVOV (n=3) by scRNA-seq. Dot color indicates log2FC EVOV versus EV and dot size indicates mean expression.**

**(G) Venn diagram showing the overlap between MHC-I IP peptide source genes and DIA-MS-detected EV/EVOV proteome genes after gene-symbol normalization.**

**Fig. S19. Survival associations of EVOV-upregulated pathway signatures across GBM bulk RNA-seq cohorts and TCGA molecular subtypes**

(A) Forest plot of Cox proportional hazards models for the association between pathway ssGSEA signature scores and overall survival in the TCGA GBM cohort, HR shown on log10 scale, 95% CI, Benjamini–Hochberg, *FDR≤0.05.

(B) Forest plot of Cox proportional hazards models for the association between pathway ssGSEA signature scores and overall survival in the CGGA GBM cohort, HR shown on log10 scale, 95% CI, Benjamini–Hochberg, *FDR≤0.05.

**(C–F) Forest plots of Cox proportional hazards models for the association between pathway ssGSEA signature scores and overall survival across TCGA GBM molecular subtypes: neural (C), proneural (D), classical (E), and mesenchymal (F), HR shown on log10 scale, 95% CI, Benjamini–Hochberg, *FDR≤0.05; multivariate models in classical and mesenchymal subtypes were adjusted for age, gender, and MGMT status.**

**Fig. S20. Survival associations of EVOV-upregulated pathway signatures in CAN-3110–treated GBM patients**

(A) Forest plot of Cox proportional hazards models for association between pre-treatment pathway ssGSEA signature scores and post-CAN-3110 survival in IDH-WT Arm A/B patients, HR shown on log10 scale, 95% CI, Benjamini–Hochberg, *FDR≤0.05.

(B) Forest plots comparing pre-treatment (red) and post-treatment (blue) pathway ssGSEA signature associations with post-CAN-3110 survival in the subset of HSV1-seropositive IDH-WT patients; left: HR with reference line HR = 1, right: p values with reference line p = 0.05; Cox proportional hazards models.
